# Supplementary material for: Swordtail fish hybrids reveal that genome evolution is surprisingly predictable after initial hybridization
Source: PLoS Biol. 2024 Aug 26;22(8):e3002742. doi: 10.1371/journal.pbio.3002742 (PMC11379403; doi:10.1371/journal.pbio.3002742)
Supplement: S1 File — (DOCX) [file pbio.3002742.s001.docx]

*Text A. Description of the X. cortezi genome assembly*

In addition to annotating protein coding genes in the *X. cortezi* genome (see main text), we annotated repetitive elements. We used RepeatMasker with a custom library of poecilid transposable elements (see Methods), which resulted in the identification of 278 Mb of repetitive sequences, largely DNA/TcMar, DNA/hAT, and LINE/L2 elements (Table S1). The most differentiated transposable element classes between the *X. birchmanni* and *X. cortezi* assemblies were LTR/ERV-Foamy elements which were twice as common in the *X. birchmanni* assembly (but only make up 2.8 Mb of the 723 Mb assembly; Table S1).

We were also interested in rearrangements that might differentiate *X. birchmanni* and *X. cortezi.* Using approximate alignments generated with minimap2 [1], we evaluated synteny across the two assemblies, and identified inverted and translocated rearrangements between the two species that exceeded 100 kb in length. We found that the two genomes were largely syntenic, with evidence of only a dozen rearrangements of this size. We found evidence for three translocations, which we treat with caution in the absence of Hi-C data for *X. cortezi*, and nine inversions ranging in size from 218 kb to 6.7 Mb (Table S2). These inversions were concentrated on chromosomes 8 and 17 (six out of nine of the inversions). We note that with one sampled individual per species, we cannot distinguish between segregating and fixed structural rearrangements, but given low genetic diversity within species relative to divergence between species (π ~ 0.1% versus D_xy_ ~ 0.6%), we predict that the majority of these rearrangements will represent fixed differences.

*Text B. Available information on dispersal capabilities in Xiphophorus and barriers between the Santa Cruz and Chapulhuacanito populations*

*Xiphophorus* species are small freshwater fish with limited dispersal capabilities. Their average standard length is 3.3 cm [2] and high genetic differentiation between geographically connected populations on the same river systems suggest that dispersal is relatively low [3,4]. Mark-recapture experiments typically recapture individuals at the same site where they were marked and the maximum distance an individual has been recaptured at from such an experiment is 0.5 km, with typical distances for dispersing individuals of <0.3 km [4,5]. Moreover, in these previous studies, the furthest moving individuals dispersed downstream, with very few individuals dispersing upstream, even at more limited distances [4,5]. Migration between Chapulhaucanito and Santa Cruz would require approximately ~60 km of upstream migration regardless of the migratory source population.

Beyond the barrier of distance between the two populations, there are other relevant barriers. In terms of suitable habitat, there is no record of *Xiphophorus* in the main stem of the much larger Río Tempoal which connects these two drainages. Moreover, the ridge separating the headwaters where these two drainages are most proximate to one another (in the Coacuilco, Hidalgo municipality) rises 70 m above the closest streambed, making headwater capture very unlikely on the short ecological timescales since these hybrid populations formed.

*Text C. Simulations of sequence mismatch and comparisons to the empirical data*

In the main text, we calculate a “mismatch” statistic between pairs of individuals in the Santa Cruz and Chapulhuacanito populations. This statistic is similar to approaches that have been used to investigate likely source populations for analyses of archaic introgression in hominins [6]. For each pair of hybrid individuals in our high coverage dataset, we identified tracts where both individuals were homozygous for *X. cortezi* ancestry (these tracts greatly exceed the number homozygous for *X. birchmanni* ancestry given admixture proportions in the two populations). Within these tracts, we identified sites where the two individuals were homozygous for different alleles, and divided this value by the total number of sites in these regions that pass our quality thresholds (see Methods). We treated this ratio as our observed “mismatch” statistic.

We find that the rate of mismatch between the Santa Cruz and Chapulhuacanito populations greatly exceeds what we observe in within population comparisons (Fig. 1G). This result suggests that the Santa Cruz and Chapulhuacanito populations – or the source populations that formed them – have been separated, but does not indicate how long they have been separated. For example, if a single hybridization event occurred with vicariance following a period of shared history, one might also expect a higher mismatch statistic in the between versus within population comparisons, without the populations being truly independent. To investigate this possibility, we used simulations, implemented in SLiM [7].

In practice, population genetic statistics will be impacted not only by the recent history of the hybrid populations but the demographic history of the parental species that contribute to the formation of these populations. Since we focus the mismatch analysis on *X. cortezi* ancestry tracts for our empirical data, we focus on modeling *X. cortezi* demographic history in our simulations. We began simulations with a burn-in period of 10*N* generations, using the estimated historical population size of *X. cortezi* from Powell et al. 2021 [8] of 51,500 individuals. We set the simulated per-bp mutation rate to the empirical mutation rate in a closely related species [9], the simulated per-bp recombination rate to 1^-8^, the region size to 500 kb, and specified that fixed mutations should not be converted to substitutions. After modeling this burn-in period, we turned to simulating the demographic history of the hybrid populations.

We performed three distinct sets of simulations, modeling shared evolution in the hybrid populations for 10, 50, or 100 generations after initial admixture. To do so, for each simulation we first randomly sampled from the posterior distributions of demographic parameters from ABC for both the Santa Cruz and Chapulhuacanito populations (see main text, Methods). We determined the minimum population size across the two sampled sets of demographic parameters, reasoning that this would be most likely to generate higher mismatch statistics, and thus be conservative for the purposes of our analyses. We next modeled 10 (or 50 or 100) generations of evolution in a single population under that population size. Following this period, we implemented a population split, and continued the simulation for each population until it had reached the target number of generations. The target number of generations was determined for each population by the randomly sampled parameter set from the ABC posterior distribution for each population, as described above. At this timepoint, we used SLiM to output a vcf for three individuals from each population and calculated the mismatch statistic as we had for the real data. We repeated this procedure 100 times for each scenario of shared evolution after initial admixture.

We found that mismatch statistics within the simulated populations closely matched the observed statistics for the real data (Fig. S5), lending credibility to our approach in terms of parameter choice and simulation design. By contrast, when we compare across the simulated populations with shared evolutionary history (either 10, 50, or 100 generations), we observe mismatch statistics that are much lower than those we calculate for the real data (Fig. S5). This indicates that the shared population evolution implemented in our simulations is strongly discordant with the patterns observed in the real data. This result provides additional evidence that the Santa Cruz and Chapulhuacanito populations are demographically independent.

Given this finding, we were next interested in determining whether we could use the observed values for the mismatch statistics as a summary statistic to investigate the approximate number of generations of separation between the *X. cortezi* source populations that contributed to the hybrid populations at Santa Cruz and Chapulhuacanito. We note that we again focus on *X. cortezi* because the majority of the genome in both the Santa Cruz and Chapulhuacanito populations is derived from *X. cortezi*. To do so, we performed SLiM simulations as described above, except that we modeled separation between two populations N generations *before* the inferred time of admixture in ABC simulations of hybrid population history. We also allowed population size to vary independently in the two populations starting at the time of separation. We used average mismatch between individuals in the two simulated populations as our summary statistic and accepted simulations within 10% of the observed between population mismatch statistic (0.046%).

In initial simulations we explored a large range of split times and population sizes. However, in practice, we found that all accepted simulations had source population split times in the last ~5,000 generations, and smaller average population sizes (<5,000 individuals). We thus focused our simulations on these parameter values, sampling from a random uniform prior for population split time between 200-5,000 and post-split population size for the two populations between 100-5,000. We performed simulations until we had accepted 500 parameter sets. We recovered well-resolved posterior distributions for split time of the source *X. cortezi* populations, as well as population size for both source populations post-split (Fig. S27). Based on these simulations, the maximum a posteriori estimate of split time between the source *X. cortezi* populations was 1,315 (95% credible intervals: 329-3099). Both the MAP and the lower 2.5% quantile exceed the estimated age of either hybrid population (Fig. S1), again suggesting that these populations do not have a shared evolutionary history that is more recent than the onset of hybridization.

*Text D. Inferred IBD tracts within and between populations*

Using resequencing data from high coverage individuals from hybrid and parental populations (N=3 per genotype per population), we ran IBDseq [10] to infer potential IBD tracts in our dataset (see Methods). Across the 24 *Xiphophorus* chromosomes, we detected 27,237 putative IBD tracts with a LOD score ≥ 3 that were longer that 10 kb. The vast majority of these IBD tracts were identified in comparisons between individuals in the same geographical location (91%, N=24,717 tracts). The median length of tracts that were identified within populations was 109 kb (Fig. S7).

Fewer tracts were identified between populations (see below), and only a tiny fraction, 0.1%, of identified tracts were shared between the two hybrid populations (31 tracts; Fig. S7). The median length of these tracts was 21 kb (Fig. S7; covering <0.3% of the genome). Notably, this tract length is shorter than the typical *X. cortezi* or *X. birchmanni* ancestry tract length observed within either hybrid population, suggesting that if these are true IBD tracts, they may date to timepoints preceding the formation of the hybrid populations.

The remainder of the tracts shared between individuals from different geographical locations were largely shared between the *X. birchmanni* Coacuilco population and pure *X. birchmanni* subpopulations at Santa Cruz and Chapulhuacanito. Eighty-two percent of all IBD tracts identified between populations (N=2,067) are attributable to shared tracts between *X. birchmanni* Coacuilco and *X. birchmanni* Santa Cruz or *X. birchmanni* Coacuilco and *X. birchmanni* Chapulhuacanito*.* The median tract length identified in these comparisons was 74 kb in both cases (Table S4). Notably, pure *X. birchmanni* at Santa Cruz and Chapulhuacanito shared many fewer IBD tracts with each other than with the *X. birchmanni* Coacuilco population (N=274), again suggesting that the populations where hybrids occur are not well-connected. These patterns are also consistent with the results of our GCTA analysis (see next section). Fewer than 100 IBD tracts were identified for other population comparisons and are listed along with information on median tract length in Table S4.

Together, these results highlight some connectivity between geographically distinct populations, but very little evidence of IBD tracts shared between individuals found in the two hybrid populations at Santa Cruz and Chapulhuacanito. Consistent with other evidence (see Text C, E, and main text), this underscores that these populations are demographically independent. However, it is important to note that with only 6 high coverage individuals per population, we have much less power to detect rare IBD from, for example, rare migration events, than would be desirable. Future data collection focusing on more rigorously characterizing IBD tracts across populations is a possible direction for future work.

*Text E. Discussion of PCA and relatedness analyses of high-coverage hybrid individuals*

In the main text, we describe a series of analyses intended to evaluate genetic variation and relatedness between individuals in the two *X. birchmanni* x *X. cortezi* hybrid populations. In particular, we were interested in evaluating any signals in the Santa Cruz and Chapulhuacanito populations that might indicate that these hybridization events were not truly independent. Although the results of these analyses support the inference that the hybrid populations are independent (see Results), they point to some other unexpected signals that we discuss in more detail here.

We identified regions that were homozygous for *X. cortezi* ancestry in all six of our high coverage hybrid individuals (three from Santa Cruz and three from Chapulhuacanito), and extracted SNPs that fell in those ancestry tracts in hybrids and pure *X. cortezi* individuals (Fig. 1E). Separately, we identified regions that were homozygous for *X. birchmanni* ancestry in all six hybrid individuals and extracted SNPs that fell in those ancestry tracts in hybrids and pure *X. birchmanni* individuals. For this analysis, we included *X. birchmanni* from an allopatric source population (Coacuilco), and pure sympatric *X. birchmanni* individuals (Fig. 1F). Our expectation for these analyses was that ancestry tracts from the two hybrid populations would separate from each other, which we observe, but that they would cluster more closely with ancestry tracts from the *X. birchmanni* individuals with which they are sympatric, as these individuals presumably are closely related to the individuals that contributed *X. birchmanni* ancestry in the hybridization event. However, we instead see that the *X. birchmanni* individuals collected from Santa Cruz and Chapulhuacanito cluster closely with each other and with the allopatric *X. birchmanni* population (Fig. 1F). Notably, we observe a similar result in GCTA analysis of the genetic relatedness matrix, with some *X. birchmanni* individuals in different populations identified as more related than average (see Methods, Fig. S6).

We are unsure of the driving forces behind this pattern but discuss several possibilities here. First, it is possible that errors in inference of the *X. birchmanni* ancestry tracts (which are more difficult to accurately delineate since they are smaller, see Text I), introduce divergent SNPs into the PCA analysis of *X. birchmanni* ancestry tracts and result in more separation between hybrids and the source *X. birchmanni* populations than expected a priori. Second, a small number of *X. birchmanni* individuals could have contributed to the initial hybrid population, resulting in genetic drift between *X. birchmanni* tracts in hybrids and sympatric *X. birchmanni* individuals. Other explanations could include that the population history of *X. birchmanni* has driven higher than expected genetic similarity between individuals from geographically distinct populations. PSMC analyses suggest that *X. birchmanni* experienced a recent bottleneck within the last ~1,000 generations [11]. Finally, dispersal distances for *X. birchmanni* could greatly exceed what has been previously assumed for this small bodied species. These explanations are also consistent with positive values of genetic relatedness detected for *X. birchmanni* in some cross-population comparisons in GCTA analyses (Fig. S6).

*Text F. Comparisons of the X. birchmanni and X. cortezi recombination map*

Past work on swordtails and the larger group to which they belong (percomorph fish) has indicated that all species studied to date carry a truncated version of PRDM9, the regulator of recombination hotspots in mammals. This version lacks the KRAB and SSXRD protein domain and is thus expected to be inactive, since these domains are essential to its function in mammals [12,13]. Consistent with this inference, both linkage disequilibrium based maps (in *X. birchmanni*) and crossover based maps (in *X. birchmanni* x *X. malinche* hybrids) indicate that recombination events largely localize to promoter-like elements including transcriptional start sites, CpG islands, and H3K4me3 peaks detected in testis ChIP-seq data [11,12].

Given that species that lack PRMD9 and localize recombination events to promoter-like elements tend to have slowly evolving recombination maps, even over millions of generations [14,15], we expected that *X. birchmanni* and *X. cortezi* would likely have nearly-identical recombination maps. However, we chose to investigate this directly by building a recombination map for *X. cortezi*, to complement the linkage-disequilibrium based map we had previously generated for *X. birchmanni* [11]. We also updated the *X. birchmanni* map with our most recent genome assembly (see Methods), which is much more complete than previous assemblies (NCBI assembly id: GCA_036418095.1).

Given that we sequenced fewer individuals in *X. cortezi* and lack access to pedigree with which to estimate mendelian errors and improve phasing (see Methods), we expect this map to be lower resolution and to have a higher error rate than the *X. birchmanni* map. Thus, we compared the two maps qualitatively to confirm that patterns of local recombination rate variation are generally concordant. Since the maps were generated in different coordinate space, we lifted over the *X. cortezi* map to *X. birchmanni* coordinate space using haltools [16]. We then compared the average ρ/bp across species in windows of varying size and found that the *X. birchmanni* and *X. cortezi* maps were strongly correlated (Fig. S9).

In previous work, we performed simulations to ask about how accurately we might expect LD-based methods to reflect the true recombination rate [11]. In these simulations we found that, as expected, inferred LD-based maps were strongly but imperfectly correlated with the true recombination map [11], with a median Spearman’s correlation across simulations between the true and inferred maps of ~0.65 (range: 0.59-0.71 in an analysis of 50 kb windows). In our comparisons of *X. birchmanni* and *X. cortezi* over the same spatial scale, we find that the Spearman’s correlation between the two maps is 0.55 (Fig. S9), slightly lower than expected if the two maps were in fact identical. Given differences in population history (see next paragraph), expected differences in error rates between the maps, and differences in chromosome architecture (Fig. S19), we consider this evidence that the *X. birchmanni* and *X. cortezi* maps are extremely similar.

We note that the median ρ/bp in *X. birchmanni* and *X. cortezi* is inferred to be different from our analyses. In *X. birchmanni*, the median ρ/bp is 0.00076 whereas in *X. cortezi* it is 0.0026. Since ρ reflects the composite value of 4*Ner*, we asked whether differences in *Ne* of *X. birchmanni* and *X. cortezi* could in part explain this difference. We reanalyzed PSMC results for individuals from the Río Huichihuayán populations of *X. cortezi* and the Coacuilco population of *X. birchmanni* from previous work [8] and asked about the inferred *Ne* of *X. birchmanni* and *X. cortezi* over the past 100,000 generations. We assumed the same generation time and mutation rate for these two species (following [8]). Using this approach, we estimated that *X. birchmanni* has had a long-term effective population size of approximately 33k over the last 100,000 generations, whereas *X. cortezi* has had an *Ne* of approximately 66k over the same time period. This suggests that the differences in estimated ρ/bp in *X. cortezi* are in part attributable to differences in population size, although differences in error rates across the two maps may also contribute.

*Text G. Simulations to explore observed cross-population correlations*

We observe strikingly strong correlations in local ancestry across the Santa Cruz and Chapulhuacanito populations. Since these two populations do not have a shared demographic history, cross-population correlations are likely generated in part by shared sources of selection across the two populations. Another possible source of shared patterns of local ancestry could be shared sources of error in local ancestry inference (see Text G-I).

Under neutrality, cross-population correlations in local ancestry are unexpected (Fig. S24). To explore whether selection could in principle generate the patterns of cross-population ancestry correlations we observe, we performed simple simulations, without attempting to directly infer the architecture of selection on *X. birchmanni* x *X. cortezi* hybrids or to simulate the full range of possible scenarios of selection on hybrids [17]. Our previous work identified 81 minor parent ancestry deserts in the Santa Cruz population [18], pointing to a reasonable starting number for simulations of loci under selection in *X. birchmanni* x *X. cortezi* hybrids.

We used the admixem program [19] to simulate admixture and selection on hybrids. We modeled 24 chromosomes, equivalent in length to the *X. birchmanni* chromosomes, and used the empirical recombination map from *X. birchmanni* to specify crossover probabilities. For each simulation, we drew other parameters, such as generations since initial admixture and initial admixture proportion, from the posterior distributions of ABC demographic inference simulations for Santa Cruz and Chapulhuacanito respectively. To model selection, we simulated recessive Dobzhansky-Muller hybrid incompatibilities. We simulated 40 incompatibilities (involving 80 loci). For each simulation, we randomly drew the chromosome and position of each locus in an incompatibility. Based on the results of initial simulations, we drew the selection coefficient from a random exponential distribution with a mean of 0.6. This distribution was truncated such that values could not exceed 1.

Because the ABC approach that we used to infer demographic history lacked selection, we found that using the inferred initial admixture proportions from ABC in simulations that included selection resulted in nearly complete purging of simulated *X. birchmanni* ancestry. This observation is in line with what other researchers have reported based on both empirical and theoretical results [20–22]. As a result, we modified initial admixture proportions in the simulations by 20% and found that in practice this results in final admixture proportions that overlapped with those observed in the empirical dataset for both simulated populations (Fig. S23 compared to Fig. 1).

We compared 50 replicate simulations of two hybrid populations. These populations differed in the simulated demographic history (which was based on ABC results for Santa Cruz and Chapulhuacanito, respectively), but experienced selection on the same pairs of hybrid incompatibility loci. We summarized the results of these simulations by calculating the average ancestry in 250 kb windows across the genome. We examined correlations in local ancestry across the two replicate populations for each simulation as we had for the real data. We found that correlations in local ancestry in simulated data overlapped those observed in the real data under this scenario of extremely strong selection (Fig. S24).

We repeated these simulations modeling partial dominance of incompatibility loci, by drawing the *h* parameter from a random uniform distribution from 0-0.5. Given that setting *h* >0 will decrease the average fitness of hybrids, we changed the average selection coefficient for this set of simulations to 0.4, but otherwise performed the simulations as described above. We again found that these simulations under this scenario resulted in correlations in ancestry that overlapped those observed in the real data (Fig. S24).

Together, these results indicate that in principle very strong selection on hybrids can drive the level of cross-population correlations in ancestry that we observe in comparisons between the Santa Cruz and Chapulhuacanito populations.

*Text H. Simulations of expected accuracy in local ancestry inference*

To evaluate the expected accuracy of our local ancestry inference approach, we used the program *mixnmatch. mixnmatch* is a pipeline designed by our lab [23] that simulates divergence between the parental lineages and admixture in hybrid populations, performs local ancestry inference, and evaluates the accuracy of that inference relative to the true simulated ancestry. We performed these simulations matching the inferred demographic history of the Santa Cruz and Chapulhuacanito populations from ABCreg analysis. To simplify these simulations, we used the MAP estimates for each demographic parameter (Fig. S1) and set the hybrid population size to 5,000. We used *mixnmatch* to simulate haplotypes for 50 diploid admixed individuals from each population and generate reads from each individual. We then ran *ancestryinfer* as we had on the real data and compared inferred ancestry at a posterior probability threshold of 0.9 to true ancestry in each simulated individual. As expected based on previous results [8,23], we found that error rates in local ancestry inference are expected to be very low (Fig. S15).

We also evaluated how shared clusters of errors might impact the correlations in ancestry inferred across populations. To do so, we performed admix’em simulations similar to those described above (Text F), except that we did not implement selection. Briefly, we simulated 24 chromosomes matching the length of the *Xiphophorus* chromosomes, tracked ancestry at 1,000 markers per chromosome, and drew simulation parameters for each population from the posterior distributions of demographic parameters produced by ABCreg. We performed 20 replicate simulations.

After the simulations had finished running, we then artificially added clusters of errors to the data. Our estimated error rate from pure parentals and artificial hybrids is ~0.1% (see Methods). However, to be conservative, we chose to simulate a high error rate of 5%. We reasoned that errors that occurred in clusters would have a larger impact on cross-population correlations than sporadic errors. As such, we randomly sampled 400 markers, and generated error tracts that continued for 3 markers (approximately 75 kb in our simulations). In both simulated populations, we converted the ancestry calls within these tracts to homozygous *X. birchmanni* ancestry in all individuals (i.e. minor parent ancestry). We then calculated average ancestry in 250 kb windows as we had in the real data, and calculated the Spearman’s correlation in ancestry across the paired simulations. We repeated this procedure 20 times.

Based on the results of these high-error simulations, we found that cross-population ancestry correlations did not approach those observed in the real data (median Spearman’s ρ = 0.32; range 0.21-0.47). Even in this conservative scenario of very high error rates and identical error profiles across all individuals, we are not able to replicate the patterns seen in our data with error alone. Together these results make us confident that the strong cross-correlations in ancestry we observe across *X. birchmanni* x *X. cortezi* populations are at least in part attributable to selection rather than errors in ancestry inference.

*Text I. Evaluating other factors that could impact correlations in ancestry across populations*

Given the extraordinarily high correlations in local ancestry that we observe between the Santa Cruz and Chapulhuacanito populations, we wanted to rigorously evaluate potential technical factors that could contribute to this pattern and be mistaken as biological signal. In addition to the analyses described in the main text (see Methods) and simulation-based methods described above, we pursued a number of additional analyses where we filtered our data using different criteria and re-evaluated correlations in local ancestry across populations.

While they are rare, we do detect errors in local ancestry inference in our analyses of known crosses (see Methods). To further investigate potential effects of error rates in the real data, we identified all likely errors genome-wide in early generation hybrids. We defined errors as instances where we observe a switch between ancestry states that reverts to the original ancestry state within 100 kb (Fig. S28). We then excluded these regions from our data from Santa Cruz and Chapulhuacanito and asked about the observed cross-correlations in ancestry in 100 kb windows. Because excluding windows with errors in any early generation hybrid results in a reduced number of windows for analysis, we generated a comparison dataset of 1,000 size-matched datasets where a matched number of windows was randomly sampled from the non-filtered data. We next compared the Spearman’s correlation in the filtered and 1,000 size-matched unfiltered datasets. The observed correlation in ancestry between the two hybrid populations in the filtered dataset was not significantly lower than that observed in size-matched unfiltered datasets (filtered = 0.76, versus average size matched = 0.78; p-value by simulation = 0.16).

We next explored the effects of removing regions of the genome where errors might be expected to be more common, but where we lacked direct evidence that errors had occurred. First, we removed all ancestry informative markers that overlapped with annotated repeats, where errors may be more common, and found that the correlations in local ancestry were unchanged (Spearman’s ρ in 250 kb windows - 0.82). We also removed windows within 1 Mb of the end of the assembled chromosome, given that error rates may be higher in these regions since some chromosomes contain telomeric sequences (Spearman’s ρ in 250 kb windows - 0.84; Fig. S19). In addition to the thinning approaches we used to evaluate the impacts of power in the main text (see Methods), we further removed all windows where the number of ancestry informative sites in the window fell in the lowest 5% or 10% genome-wide and re-evaluated correlations between populations (Spearman’s ρ 0.82-0.83 in 250 kb windows across comparisons). We performed similar analyses excluding windows where we have the highest power to infer ancestry (upper 5 or 10% of ancestry informative site density) and again saw that correlations in ancestry between the two populations remained strong (Spearman’s ρ 0.79-0.81 in 250 kb windows across comparisons). We also evaluated combinations of these analyses (i.e. removing repetitive windows, windows within 1 Mb of chromosome edges, and low-power windows). In no case did these modifications substantially change the observed cross-population correlations.

In summary, our analyses in the main text and those described above indicated that errors and variation in power to infer ancestry were very unlikely to generate the cross-population correlations we observe in the real data. However, we have a clear expectation that ancestry correlations across distinct hybrid populations should be substantially lower than those observed when analyzing ancestry covariance within the same population. Thus, we wanted to evaluate the range of correlations expected from subsampling individuals from the same population (in addition to the cross-year and within-drainage comparisons discussed in the main text) and to compare these with our cross-population results. We generated replicated datasets where we randomly subsampled 20 individuals from the same population and collection year and calculated average ancestry along the genome. Across these subsampled replicates, we consistently observed correlations in local ancestry in 250 kb windows of ≥0.9 in subsampled replicates from both Santa Cruz and from Chapulhuacanito. Reassuringly, these correlations from subsampling the same population greatly exceed the correlations observed when comparing ancestry across the two populations.

Overall, our results are not consistent with technical factors driving cross-population correlations and instead point to biological factors, such as shared sources of selection, driving the genome-wide correlations in ancestry that we observe in *X. birchmanni* x *X. cortezi* hybrids.

*Text J. Additional analyses and discussion of spatial patterns from the Discrete Wavelet Transform analysis*

We performed wavelet analyses of correlations using two different interpolation resolutions of the ancestry estimate. The ancestry statistic is an estimate of the minor parent allele frequency within a diploid individual, calculated as a weighted average of marginal posterior probabilities of two genotypes:

f_A_ = P(AA) + ½ P(Aa)

We subsequently average these interpolated measures across individuals to give an estimate of the population admixture proportion. In one case, we interpolate ancestry to a resolution of 32 kb. This corresponds roughly to the average tract length of minor parent ancestry in the Santa Cruz population. We separately interpolate to a 1 kb grid, corresponding roughly to the density of ancestry informative markers.

Our analyses of cross-population ancestry correlations revealed strong correlations persisting at fine genomic scales (Fig. S17). We interpret these correlations with caution, as they could be in part driven by correlated fine-scale error in the ancestry inference procedure (see details below). Moreover, we also expect that since the majority of ancestry ‘tracts,’ defined on the basis of high-confidence transitions between ancestry states, are greater than 32 kb in both *X. birchmanni* x *X. cortezi* hybrid populations, local ancestry inference will be less reliable at these fine scales (see also below). That said, some of these fine scale correlations may indeed be capturing real biological signals. This is because the wavelet analysis directly uses the posterior probability of each ancestry state. As a result, even short ancestry tracts that are not called with high confidence could generate spatial variation in marginal posterior probabilities that correlates with true ancestry states. Furthermore, we might expect that a bias towards detection of minor parent ancestry would be stronger in lower recombination regions. However, if this were true, we would expect to see negative correlations between minor parent ancestry and recombination at these scales, which we do not (Fig. S16). Finally, we note that the pattern we observe is consistent with expectations that hybrid populations formed between more deeply divergent species will have a higher density of selected sites. Fine-scale correlations in the analysis of *X. birchmanni* x *X. cortezi* populations are substantially elevated compared to correlations at fine-scales in analysis of *X. birchmanni* x *X. malinche* populations (Fig. S17). This pattern suggests that the repeatability we observe at small spatial scales in *X. birchmanni* x *X. cortezi* populations may be driven by stronger selection on hybrids in this more divergent cross.

While we expect our local ancestry inference approach to be highly accurate overall, there are several reasons to be cautious about patterns detected at the finest spatial scales. Past simulation studies have indicated that *ancestryinfer* has a much higher error rate in shorter ancestry tracts [23]. Shorter ancestry tracts will contain fewer ancestry informative sites with which to infer local ancestry. Moreover, because of concerns about inducing correlations between recombination rate and minor parent ancestry [23], *ancestryinfer* uses a uniform recombination prior, which may further increase the difficulty of detecting short ancestry tracts.

To evaluate expected error rate in short ancestry tracts directly, we used *mixnmatch* simulations matching the demographic history of the Santa Cruz population. We expect that simulations of Santa Cruz will result in higher estimates of error because minor parent ancestry tracts are shorter on average in Santa Cruz than in Chapulhuacanito. This is because Santa Cruz has an older estimated admixture time (Fig. S1) and a more skewed admixture proportion towards *X. cortezi*, which will both contribute to shorter minor parent ancestry tracts [24]. Using *mixnmatch* simulations, we evaluated accuracy in simulations of 50 individuals as a function of ancestry tract length. While genome-wide error rates per ancestry informative site are estimated to be <0.4% (see Text G), in ancestry tracts ≤20 kb in length, the error rate approaches 2%, and in tracts ≤10 kb in length, the error rates jump to nearly 5%. A closer examination of these errors indicates that they are almost exclusively caused by ancestry switches to homozygous major parent ancestry within heterozygous ancestry tracts (94% of errors). As a result, we interpret results of wavelet analyses at small spatial scales with caution and refrain from discussing them in the main text.

*Text K. Results of power simulations evaluating our ability to identify shared regions under selection*

Given that we identify ~40 shared minor parent ancestry deserts between the two *X. birchmanni* x *X. cortezi* populations in the real data, we were interested in exploring our power to identify shared ancestry deserts at a range of selection coefficients. We used admix’em simulations as described above (Text G) to simulate selection against minor parent ancestry in two populations at a range of strengths (*s*=0.01 – 0.1, with *h*=0.5). As before, for each replicate simulation, we drew from the posterior distribution of ABCreg analysis to set demographic parameters for paired simulations modeling admixture in Santa Cruz and Chapulhuacanito. We randomly determined the chromosome and position of the selected site in each simulation. We performed 100 replicate simulations per selection coefficient.

For each simulation, we asked whether an ancestry desert was detected in the simulated Santa Cruz population, the Chapulhuacanito population, or both, as we had for the real data (see Methods). If this ancestry desert overlapped with the true site under selection, we treated it as a true positive, and if it occurred on a chromosome where selection was not occurring, we treated it as a false positive. We used the proportion of time the desert overlapped with the true site under selection as an estimate of our power to detect deserts at a given selection coefficient.

Overall, we found that we had excellent power to detect stronger selection (s=0.1) in both Santa Cruz and Chapulhuacanito, and good power to detect more moderate selection (Fig. S29; >50% of selected sites with s=0.025 and ~70% of selected sites with s=0.05). We also found that the false positive rate was low (with an average of 2-4 false positives detected per simulation).

These simulation results suggest that we have excellent power to identify shared ancestry deserts in Santa Cruz and Chapulhuacanito, even when selection is modest. Moreover, the expected false positive rate is quite low. This indicates that the shared ancestry deserts identified in our empirical data are likely true sites under selection in *X. birchmanni* x *X. cortezi* hybrids, providing exciting candidates for further work exploring hybrid incompatibilities and the architecture of reproductive isolation between these two species.

*Text L. Enrichment analysis of genes occurring in shared islands*

Since regions of high minor parent ancestry in both hybrid populations may reflect regions that have adaptively introgressed from *X. birchmanni*, we were interested in exploring whether any functional classes might be over-represented in shared minor parent islands using a gene ontology based approach. We lifted over the coordinates of shared minor parent islands identified in both Santa Cruz and Chapulhuacanito to the *X. maculatus* 5.0 (GCA_002775205.2) assembly using haltools. We then used bedtools to identify the Ensembl gene ids of the genes that fell within these islands.

*X. maculatus* Ensembl IDs were matched with GO term IDs using the R packages ‘biomaRt’ [25] and ‘GOstats’ [26]. This set of genes was used as the reference gene set. To identify GO terms with overrepresentation within the high *X. birchmanni* ancestry genes in the minor parent islands, we used a hypergeometric test implemented through the R package ‘GSEABase’ [27] to statistically compare observed gene counts with expected gene counts per GO term, given the reference gene set. We ran this analysis for the biological pathway, molecular function, and cellular component categories, and used a hypergeometric test p-value cutoff of 0.05 to pull significantly enriched GO terms.

Because genes with similar function sometimes colocalize in the genome, we wanted to build null expectations for gene otology enrichment that might be expected by chance. To do so, we generated null islands. For each island, we simulated a “null island” by randomly selected a start position in the genome and setting the stop position to the start position plus the length of the islands. We repeated this until we had a complete dataset of null islands and generated a total of 10 null datasets. Next, we repeated the gene otology enrichment analysis we had applied to the real data and evaluated how many and which categories were enriched in our null datasets.

Although we identified significantly enriched gene otology categories (Table S15), we avoid interpreting these results based on the results of the null datasets discussed above. Specifically, we find that neither the number of gene otology categories identified in the real analysis, nor the specific categories identified are unusual when compared to the null datasets.

*Text M. Other drivers of variation in minor parent ancestry*

We were interested in annotating genes involved in protein complexes since past work in swordtails has highlighted that mismatch in ancestry in protein complexes can have a substantial impact on hybrid survival [28]. We used a large database of curated protein complexes annotated in humans [29] and identified reciprocal best blast hits to this database. Although swordtails are distantly related to humans, this represents the most complete dataset of protein complexes available for any species.

We used the program orthologr and the command blast_rec [30] to identify the reciprocal best blast hit for all protein coding genes in the human genome. We used the hg38 assembly (Homo_sapiens.GRCh38.cds.all.fa release-110) from Ensembl and the predicted cDNA sequences from the genome assembly for *X. birchmanni*. Since all teleost fish have undergone an ancient whole genome duplication, there were a substantial number of genes that returned multiple hits from the blast_rec command. We assigned an ortholog as a reciprocal best blast hit if there was a single ortholog, or if there was an ortholog with a lower e-value than other hits. We note that the total number of 1:1 orthogs that we are able to identify is likely impacted by the teleost whole genome duplication.

We downloaded the HuMAP2_IDs of genes that are involved in protein complexes in humans from <http://humap2.proteincomplexes.org>. We converted these to ensembl id using the MANE database (<https://www.ncbi.nlm.nih.gov/refseq/MANE/>). The HuMAP2_IDs were then matched to *X. birchmanni* orthologs of these genes and their coordinates in the *X. birchmanni* reference genome. This resulted in a dataset of 5,118 *X. birchmanni* genes where the human ortholog is involved in a protein complex.

With these coordinates in hand, we next evaluated average ancestry in regions containing genes involved in protein complexes compared to expectations by chance. We compared average ancestry of protein complex genes in Chapulhuacanito and Santa Cruz to null datasets. To generate null datasets, we randomly selected 5,118 genes not annotated as being involved in protein complexes, calculated average ancestry, and repeated this process 1,000 times. We repeated this analysis using any gene with a 1:1 ortholog between *X. birchmanni* and humans as the focal set, and again compared ancestry in these regions to 1,000 null datasets, each with 5,118 genes (sampled out of a total of 11,289 genes with 1:1 orthologs).

While we found that genes involved in protein complexes had lower average minor parent ancestry in *X. birchmanni* x *X. cortezi* hybrid populations than average minor parent ancestry across all protein-coding regions, the depletion of minor parent ancestry was not significantly lower than observed for other genes with 1:1 orthologs (Fig. S21). This suggests that the signal we detect is simply a consequence of such genes being under greater evolutionary constraint. However, this does not rule out the possibility that certain protein complexes are especially depleted in minor parent ancestry [28].

*Text N. Estimating the length of X. birchmanni chromosomes in centimorgans*

For several of the analyses in the paper, it is useful to consider the length of each chromosome in centimorgans (cM). To generate these estimates, we took advantage of artificial crosses between *X. birchmanni* and *X. malinche* generated in the lab between F_1_ parents, which yielded a large dataset of 1253 second generation hybrids. Because we expect recombination maps to be conserved across *Xiphophorus* species (see Text F), crossover maps give us a more direct (albeit coarser) estimate of the recombination rate, that can be used to convert physical to genetic distance.

Because F_1_ individuals are heterozygous for ancestry at every location in their genome, any ancestry transitions observed in F_2_ hybrids (apart from those attributable to error), reflect crossover events that occurred during meiosis in the F_1_ parents. We were thus interested in identifying the number of ancestry transition events per individual on each chromosome to estimate the genetic length of the chromosomes. We also wanted to exclude switch errors where possible, which will have the effect of inflating our estimates of genetic length. We identified and excluded ancestry switches that occurred within 250 kb of each other, as we expect only one crossover per chromosome per meiosis in F_2_ hybrids and such excess crossovers likely represent errors. We excluded individuals with an extremely high numbers of crossovers across the genome (>75; 12 individuals).

Following this filtering, we recorded the number of crossovers (i.e. ancestry transitions) per individual per chromosome. For each chromosome, we calculated the average number of crossovers between the first and last basepair on the assembled chromosome. Since each F_2_ individual is the product of two meiosis events, we divided this number by two. We treated this value as the average frequency of recombination events across the length of this chromosome during meiosis. By multiplying this number by 100, we arrived at an estimate of the probability of recombination over the chromosome per meiosis, or the length of each chromosome in centimorgans. We used these estimates of the length of the chromosome in centimorgans, combined with our linkage-disequilibrium maps to roughly convert estimates of ρ/bp to genetic distance, and determine intervals of a given genetic distance across the 24 chromosome (e.g. to generate 0.1 cM windows).

**S1 File References**

1. Li H. Minimap2: pairwise alignment for nucleotide sequences. Bioinformatics. 2018;34: 3094–3100. doi:10.1093/bioinformatics/bty191

2. Preising GA, Gunn T, Baczenas JJ, Pollock A, Powell DL, Dodge TO, et al. Recurrent evolution of small body size and loss of the sword ornament in Northern Swordtail fish. bioRxiv; 2022. p. 2022.12.24.521833. doi:10.1101/2022.12.24.521833

3. Gutiérrez-Rodríguez C, Morris MR, Dubois NS, Queiroz KD. Genetic variation and phylogeography of the swordtail fish Xiphophorus cortezi (Cyprinodontiformes, Poeciliidae). Molecular Phylogenetics and Evolution. 2007;43: 111–123. doi:10.1016/j.ympev.2006.10.022

4. Tatarenkov A, Healey CIM, Avise JC. Microgeographic population structure of green swordail fish: genetic differentiation despite abundant migration. Molecular Ecology. 2010;19: 257–268. doi:10.1111/j.1365-294X.2009.04464.x

5. Culumber ZW, Ochoa OM, Rosenthal GG. Assortative Mating and the Maintenance of Population Structure in a Natural Hybrid Zone. The American Naturalist. 2014;184: 225–232. doi:10.1086/677033

6. Browning SR, Browning BL, Zhou Y, Tucci S, Akey JM. Analysis of Human Sequence Data Reveals Two Pulses of Archaic Denisovan Admixture. Cell. 2018;173: 53-61.e9. doi:10.1016/j.cell.2018.02.031

7. Haller BC, Messer PW. SLiM 3: Forward Genetic Simulations Beyond the Wright–Fisher Model. Hernandez R, editor. Molecular Biology and Evolution. 2019;36: 632–637. doi:10.1093/molbev/msy228

8. Powell DL, Moran BM, Kim BY, Banerjee SM, Aguillon SM, Fascinetto-Zago P, et al. Two new hybrid populations expand the swordtail hybridization model system. Evolution. 2021;75: 2524–2539. doi:10.1111/evo.14337

9. Burda K, Konczal M. Validation of machine learning approach for direct mutation rate estimation. Molecular Ecology Resources. 2023;23: 1757–1771. doi:10.1111/1755-0998.13841

10. Browning BL, Browning SR. Detecting identity by descent and estimating genotype error rates in sequence data. Am J Hum Genet. 2013;93: 840–851. doi:10.1016/j.ajhg.2013.09.014

11. Schumer M, Xu C, Powell DL, Durvasula A, Skov L, Holland C, et al. Natural selection interacts with recombination to shape the evolution of hybrid genomes. Science. 2018;360: 656. doi:10.1126/science.aar3684

12. Baker Z, Schumer M, Haba Y, Bashkirova L, Holland C, Rosenthal GG, et al. Repeated losses of PRDM9-directed recombination despite the conservation of PRDM9 across vertebrates. In: eLife [Internet]. 6 Jun 2017 [cited 23 Jul 2019]. doi:10.7554/eLife.24133

13. Cavassim MIA, Baker Z, Hoge C, Schierup MH, Schumer M, Przeworski M. PRDM9 losses in vertebrates are coupled to those of paralogs ZCWPW1 and ZCWPW2. bioRxiv; 2021. p. 2021.06.08.447603. doi:10.1101/2021.06.08.447603

14. Singhal S, Leffler EM, Sannareddy K, Turner I, Venn O, Hooper DM, et al. Stable recombination hotspots in birds. Science. 2015;350: 928–932. doi:10.1126/science.aad0843

15. Lam I, Keeney S. Nonparadoxical evolutionary stability of the recombination initiation landscape in yeast. Science. 2015;350: 932–937. doi:10.1126/science.aad0814

16. Armstrong J, Hickey G, Diekhans M, Deran A, Fang Q, Xie D, et al. Progressive alignment with Cactus: a multiple-genome aligner for the thousand-genome era. bioRxiv. 2019; 730531. doi:10.1101/730531

17. Moran BM, Payne C, Langdon Q, Powell DL, Brandvain Y, Schumer M. The genomic consequences of hybridization. Wittkopp PJ, editor. eLife. 2021;10: e69016. doi:10.7554/eLife.69016

18. Langdon QK, Powell DL, Kim B, Banerjee SM, Payne C, Dodge TO, et al. Predictability and parallelism in the contemporary evolution of hybrid genomes. PLOS Genetics. 2022;18: e1009914. doi:10.1371/journal.pgen.1009914

19. Cui R, Schumer M, Rosenthal GG. Admix’em: a flexible framework for forward-time simulations of hybrid populations with selection and mate choice. Bioinformatics. 2016;32: 1103–1105. doi:10.1093/bioinformatics/btv700

20. Harris K, Nielsen R. The Genetic Cost of Neanderthal Introgression. Genetics. 2016;203: 881–891. doi:10.1534/genetics.116.186890

21. Matute DR, Comeault AA, Earley E, Serrato-Capuchina A, Peede D, Monroy-Eklund A, et al. Rapid and Predictable Evolution of Admixed Populations Between Two Drosophila Species Pairs. Genetics. 2019 [cited 20 Apr 2020]. doi:10.1534/genetics.119.302685

22. Veller C, Edelman NB, Muralidhar P, Nowak MA. Recombination and Selection Against Introgressed DNA. Evolution. 2023; qpad021. doi:10.1093/evolut/qpad021

23. Schumer M, Powell DL, Corbett-Detig R. Versatile simulations of admixture and accurate local ancestry inference with mixnmatch and ancestryinfer. Mol Ecol Resour. 2020;20: 1141–1151. doi:10.1111/1755-0998.13175

24. Gravel S. Population Genetics Models of Local Ancestry. Genetics. 2012;191: 607. doi:10.1534/genetics.112.139808

25. Durinck S, Spellman PT, Birney E, Huber W. Mapping identifiers for the integration of genomic datasets with the R/Bioconductor package biomaRt. Nat Protoc. 2009;4: 1184–1191. doi:10.1038/nprot.2009.97

26. Falcon S, Gentleman R. Using GOstats to test gene lists for GO term association. Bioinformatics. 2007;23: 257–258. doi:10.1093/bioinformatics/btl567

27. Morgan, Martin, Falcon S, Gentleman R. GSEABase: Gene set enrichment data structures and methods version 1.52.1 from Bioconductor. [cited 5 Aug 2021]. Available: https://rdrr.io/bioc/GSEABase/

28. Moran BM, Payne CY, Powell DL, Iverson ENK, Banerjee SM, Langdon QK, et al. A Lethal Genetic Incompatibility between Naturally Hybridizing Species in Mitochondrial Complex I. 2021 Jul p. 2021.07.13.452279. doi:10.1101/2021.07.13.452279

29. Drew K, Lee C, Huizar RL, Tu F, Borgeson B, McWhite CD, et al. Integration of over 9,000 mass spectrometry experiments builds a global map of human protein complexes. Molecular Systems Biology. 2017;13: 932. doi:10.15252/msb.20167490

30. Drost H-G, Gabel A, Grosse I, Quint M. Evidence for Active Maintenance of Phylotranscriptomic Hourglass Patterns in Animal and Plant Embryogenesis. Molecular Biology and Evolution. 2015;32: 1221–1231. doi:10.1093/molbev/msv012

31. Groh JS, Coop G. The temporal and genomic scale of selection following hybridization. bioRxiv; 2023. p. 2023.05.25.542345. doi:10.1101/2023.05.25.542345

32. Aguillon SM, Cox SKH, Langdon QK, Gunn TR, Baczenas JJ, Banerjee SM, et al. Pervasive gene flow despite strong and varied reproductive barriers in swordtails. bioRxiv; 2024. p. 2024.04.16.589374. doi:10.1101/2024.04.16.589374
