# Supplementary material for: Swordtail fish hybrids reveal that genome evolution is surprisingly predictable after initial hybridization
Source: PLoS Biol. 2024 Aug 26;22(8):e3002742. doi: 10.1371/journal.pbio.3002742 (PMC11379403; doi:10.1371/journal.pbio.3002742)
Supplement: S1 Table — birchmanni and X. cortezi with more than a 10% copy number difference in the PacBio HiFi genome assemblies of the 2 species. (DOCX) [file pbio.3002742.s002.docx]

**Table S1.** Transposable element classes annotated in *X. birchmanni* and *X. cortezi* with more than a 10% copy number difference in the PacBio HiFi genome assemblies of the two species.

| **Element annotation** | ***X. birchmanni* copy number** | ***X. cortezi* copy number** |
| --- | --- | --- |
| DNA:CMC-EnSpm | 4633756 | 4175251 |
| DNA:Crypton-S | 81900 | 64962 |
| DNA:Dada | 24369 | 21879 |
| DNA:Kolobok-Hydra | 26334 | 22327 |
| DNA:P | 502814 | 440376 |
| DNA:PiggyBac | 542091 | 479037 |
| DNA:RC | 8159880 | 7159251 |
| DNA:Sola-3 | 196508 | 110878 |
| DNA:Zisupton | 351011 | 317145 |
| LINE:I | 2377386 | 2142649 |
| LINE:L1-Tx1 | 1853402 | 1579130 |
| LINE:Proto2 | 268094 | 233548 |
| SINE:tRNA-L1 | 1347298 | 1184614 |
| LTR:ERV-Foamy | 2814375 | 1289681 |
| LTR:ERVK | 388961 | 318244 |
| LTR:ERVL | 9367 | 7272 |
| LTR:Gypsy | 4993068 | 4287995 |
| LINE:RTE-X | 31866 | 27078 |
| DNA:Crypton | 10491 | 8429 |
| DNA:TcMar-Ant1 | 1560 | 1218 |
| DNA:CMC-Transib | 384060 | 439064 |
| DNA:Ginger-2 | 513 | 2570 |
| DNA:Novosib | 16452 | 19915 |
| DNA:PIF-Spy | 3542 | 4456 |
| DNA:TcMar-Mariner | 132570 | 192627 |
| LINE:I-Jockey | 164819 | 258738 |
| LINE:Penelope | 486258 | 601090 |
| LINE:R2-NeSL | 70333 | 77645 |
| LINE:RTE-BovB | 4146214 | 4593954 |
| SINE:tRNA-V-CR1 | 30157 | 34081 |
| LTR:Copia | 644541 | 721754 |
| LINE:Tad1 | 42067 | 46962 |
