# Supplementary material for: Swordtail fish hybrids reveal that genome evolution is surprisingly predictable after initial hybridization
Source: PLoS Biol. 2024 Aug 26;22(8):e3002742. doi: 10.1371/journal.pbio.3002742 (PMC11379403; doi:10.1371/journal.pbio.3002742)
Supplement: S2 Table — Genome assemblies were aligned with minimap2 and all inversions greater than 100 kb were annotated. (DOCX) [file pbio.3002742.s003.docx]

**Table S2.** Inversions that differentiate *X. birchmanni* and *X. cortezi* based on our PacBio HiFi assemblies. Genome assemblies were aligned with minimap2 and all inversions greater than 100 kb were annotated.

| **Chromosome** | **Start coordinate (*X. birchmanni*)** | **End coordinate (*X. birchmanni*)** |
| --- | --- | --- |
| chr-07 | 6637497 | 7241732 |
| chr-08 | 6542350 | 12967418 |
| chr-08 | 28401987 | 29237938 |
| chr-08 | 13072933 | 13389204 |
| chr-17 | 4580825 | 7858932 |
| chr-17 | 13620980 | 16130397 |
| chr-17 | 7898896 | 9230698 |
| chr-18 | 34136089 | 34325628 |
| chr-24 | 5850012 | 7595198 |
