# Supplementary material for: Swordtail fish hybrids reveal that genome evolution is surprisingly predictable after initial hybridization
Source: PLoS Biol. 2024 Aug 26;22(8):e3002742. doi: 10.1371/journal.pbio.3002742 (PMC11379403; doi:10.1371/journal.pbio.3002742)
Supplement: S3 Table — (DOCX) [file pbio.3002742.s004.docx]

**Table S3.** MAP estimate and 95% percent confidence intervals for posterior distributions generated by ABCreg for each demographic parameter of interest.

| **Parameter (prior)** | **Chapulhuacanito 2.5% quantile** | **Chapulhuacanito MAP estimate** | **Chapulhuacanito 97.5% quantile** | **Santa Cruz 2.5% quantile** | **Santa Cruz MAP estimate** | **Santa Cruz 97.5% quantile** |
| --- | --- | --- | --- | --- | --- | --- |
| Population size  (2-10,000) | 734 | 3113 | 9760 | 941 | 5652 | 9651 |
| Generations since admixture (10-400) | 104 | 137 | 317 | 218 | 263 | 383 |
| Migration – species 1 (0-3%) | 0 | 0.017% | 2.4% | 0 | 0.028% | 2.3% |
| Migration – species 2 (0-3%) | 0 | 0.004% | 0.11% | 0 | 0.004% | 0.05% |
| Initial admixture proportion (0.5-1) | 0.76 | 0.86 | 0.96 | 0.81 | 0.86 | 0.97 |
