# Supplementary material for: Swordtail fish hybrids reveal that genome evolution is surprisingly predictable after initial hybridization
Source: PLoS Biol. 2024 Aug 26;22(8):e3002742. doi: 10.1371/journal.pbio.3002742 (PMC11379403; doi:10.1371/journal.pbio.3002742)
Supplement: S4 Table — Group 1 indicates the genotype and population for focal group 1, and Group 2 indicates the genotype and population for focal group 2. CHPL, Chapulhuacanito; STAC, Santa Cruz; COAC, Coacuilco; HUIC, Huichihuayán; PTHC, Puente de Huichihuayán. Comparison type indicates whether individuals were sampled from the same or different geographical locations. Number of tracts is the number of IBD segments identified by IBDseq. The median length of these tracts in basepairs and the upper 95% quantile is also reported for cases with a sufficient number of tracts. (DOCX) [file pbio.3002742.s005.docx]

**Table S4.** Results of IBDseq analysis of high coverage data for three individuals per genotype per population. Group 1 indicates the genotype and population for focal group 1, and Group 2 indicates the genotype and population for focal group 2. Abbreviations: CHPL – Chapulhuacanito, STAC – Santa Cruz, COAC- Coacuilco, HUIC – Huichihuayán, PTHC – Puente de Huichihuayán. Comparison type indicates whether individuals were sampled from the same or different geographical locations. Number of tracts is the number of IBD segments identified by IBDseq. The median length of these tracts in basepairs and the upper 95% quantile is also reported for cases with a sufficient number of tracts.

| **Group 1** | **Group 2** | **Comparison type** | **Number of tracts** | **50% length quantile (bp)** | **95% length quantile (bp)** |
| --- | --- | --- | --- | --- | --- |
| Hybrid - CHPL | Hybrid - CHPL | Within population | 2659 | 96116 | 543077 |
| Hybrid - STAC | Hybrid - STAC | Within population | 4368 | 100137 | 583950 |
| X. birchmanni - CHPL | X. birchmanni - CHPL | Within population | 2690 | 97925 | 426577 |
| X. birchmanni - STAC | X. birchmanni - STAC | Within population | 2030 | 144104 | 666619 |
| X. birchmanni - COAC | X. birchmanni - COAC | Within population | 7045 | 133412 | 521598 |
| X. cortezi - HUIC | X. cortezi - HUIC | Within population | 1092 | 77594 | 1577780 |
| X. cortezi - PTHC | X. cortezi - PTHC | Within population | 1198 | 67882 | 489418 |
| X. birchmanni - STAC | Hybrid - STAC | Within population | 245 | 71396 | 329143 |
| X. birchmanni - CHPL | Hybrid - CHPL | Within population | 393 | 52859 | 206439 |
| X. birchmanni - CHPL | X. birchmanni - COAC | Between population | 1129 | 74305 | 264057 |
| X. cortezi - HUIC | X. cortezi - PTHC | Between population | 1103 | 77066 | 1536298 |
| X. birchmanni - COAC | X. birchmanni - STAC | Between population | 938 | 74253 | 281609 |
| X. birchmanni - CHPL | X. birchmanni - STAC | Between population | 274 | 59892 | 197262 |
| X. birchmanni - COAC | Hybrid - CHPL | Between population | 75 | 43239 | 147618 |
| X. birchmanni - COAC | Hybrid - STAC | Between population | 26 | 24866 | 67456 |
| Hybrid - CHPL | Hybrid - STAC | Between population | 25 | 27491 | 233188 |
| Hybrid - CHPL | X. cortezi - PTHC | Between population | 8 | 46598 | 243972 |
| X. birchmanni - STAC | Hybrid - CHPL | Between population | 19 | 34289 | 205881 |
| Hybrid - CHPL | X. cortezi - HUIC | Between population | 4 | - | - |
| X. birchmanni - CHPL | Hybrid - STAC | Between population | 18 | 19760 | 122654 |
| X. birchmanni - COAC | X. cortezi - HUIC | Between population | 1 | - | - |
| X. birchmanni - COAC | X. cortezi - PTHC | Between population | 1 | - | - |
| Hybrid - STAC | X. cortezi - HUIC | Between population | 2 | - | - |
