# Supplementary material for: Swordtail fish hybrids reveal that genome evolution is surprisingly predictable after initial hybridization
Source: PLoS Biol. 2024 Aug 26;22(8):e3002742. doi: 10.1371/journal.pbio.3002742 (PMC11379403; doi:10.1371/journal.pbio.3002742)
Supplement: S5 Table — Windows of 100–500 kb are reported here. Since both ancestry and recombination rate are spatially correlated along the genome, for each analysis, we thinned the data to include 1 window per Mb, such that all analyses presented below include the same number of windows. Population names in italics indicate the focal samples primarily discussed in the main text. (DOCX) [file pbio.3002742.s006.docx]

**Table S5.** Results of the analysis of the correlation between local minor parent ancestry in windows of varying size versus the average recombination rate in that window. Windows of 100 – 500 kb are reported here. Since both ancestry and recombination rate are spatially correlated along the genome, for each analysis, we thinned the data to include one window per Mb, such that all analyses presented below include the same number of windows. Population names in italics indicate the focal samples primarily discussed in the main text.

| **Population** | **Window size** | **Spearman’s ρ** | **Thinned p-value** |
| --- | --- | --- | --- |
| *Santa Cruz 2020* | 100 kb | 0.50 | <10^-100^ |
| *Chapulhuacanito 2021* | 100 kb | 0.55 | <10^-56^ |
| Chapulhuacanito 2003 | 100 kb | 0.50 | <10^-43^ |
| Chapulhuacanito 2006 | 100 kb | 0.51 | <10^-47^ |
| Chapulhuacanito 2017 | 100 kb | 0.55 | <10^-55^ |
| Huextetitla 2003 | 100 kb | 0.50 | <10^-44^ |
| Huextetitla 2019 | 100 kb | 0.49 | <10^-43^ |
| *Santa Cruz 2020* | 250 kb | 0.58 | <10^-66^ |
| *Chapulhuacanito 2021* | 250 kb | 0.58 | <10^-65^ |
| Chapulhuacanito 2003 | 250 kb | 0.57 | <10^-61^ |
| Chapulhuacanito 2006 | 250 kb | 0.59 | <10^-66^ |
| Chapulhuacanito 2017 | 250 kb | 0.60 | <10^-70^ |
| Huextetitla 2003 | 250 kb | 0.57 | <10^-61^ |
| Huextetitla 2019 | 250 kb | 0.57 | <10^-61^ |
| *Santa Cruz 2020* | 500 kb | 0.63 | <10^-81^ |
| *Chapulhuacanito 2021* | 500 kb | 0.66 | <10^-88^ |
| Chapulhuacanito 2003 | 500 kb | 0.63 | <10^-78^ |
| Chapulhuacanito 2006 | 500 kb | 0.65 | <10^-85^ |
| Chapulhuacanito 2017 | 500 kb | 0.64 | <10^-84^ |
| Huextetitla 2003 | 500 kb | 0.64 | <10^-83^ |
| Huextetitla 2019 | 500 kb | 0.62 | <10^-75^ |
