# Supplementary material for: Swordtail fish hybrids reveal that genome evolution is surprisingly predictable after initial hybridization
Source: PLoS Biol. 2024 Aug 26;22(8):e3002742. doi: 10.1371/journal.pbio.3002742 (PMC11379403; doi:10.1371/journal.pbio.3002742)
Supplement: S6 Table — Since recombination rate has a strong impact on local ancestry (S5 Table), this analysis attempts to control for this effect by accounting for local recombination rate. We repeated this analysis in windows of a particular genetic length (S7 Table). As before, we thinned windows to include only 1 per Mb. Population names in italics indicate the focal samples primarily discussed in the main text. (DOCX) [file pbio.3002742.s007.docx]

**Table S6.** Results of the analysis of the partial correlation between local minor parent ancestry and the number of linked coding and conserved basepairs. Since recombination rate has a strong impact on local ancestry (Table S5), this analysis attempts to control for this effect by accounting for local recombination rate. We repeated this analysis in windows of a particular genetic length (Table S7). As before, we thinned windows to include only one per Mb. Population names in italics indicate the focal samples primarily discussed in the main text.

| **Population** | **Window size** | **Partial correlation recombination rate (p-value)** | **Partial correlation coding (p-value)** | **Partial correlation conserved (p-value)** |
| --- | --- | --- | --- | --- |
| *Santa Cruz 2020* | 100 kb | 0.48 (<10^-41^) | 0.08 (0.039) | -0.03 (0.40) |
| *Chapulhuacanito 2021* | 100 kb | 0.54 (<10^-54^) | 0.02 (0.58) | -0.02 (0.61) |
| Chapulhuacanito 2003 | 100 kb | 0.49 (<10^-42^) | -0.018 (0.64) | -0.009 (0.81) |
| Chapulhuacanito 2006 | 100 kb | 0.50 (<10^-45^) | 0.035 (0.35) | -0.008 (0.83) |
| Chapulhuacanito 2017 | 100 kb | 0.55 (<10^-54^) | -0.028 (0.45) | 0.002 (0.95) |
| Huextetitla 2003 | 100 kb | 0.49 (<10^-41^) | 0.045 (0.19) | -0.017 (0.65) |
| Huextetitla 2019 | 100 kb | 0.48 (<10^-41^) | 0.02 (0.56) | -0.001 (0.96) |
| *Santa Cruz 2020* | 250 kb | 0.57 (<10^-62^) | 0.11 (0.005) | -0.088 (0.019) |
| *Chapulhuacanito 2021* | 250 kb | 0.58 (<10^-63^) | 0.055 (0.14) | -0.078 (0.035) |
| Chapulhuacanito 2003 | 250 kb | 0.56 (<10^-59^) | 0.034 (0.37) | -0.061 (0.11) |
| Chapulhuacanito 2006 | 250 kb | 0.58 (<10^-63^) | 0.056 (0.14) | -0.046 (0.22) |
| Chapulhuacanito 2017 | 250 kb | 0.60 (<10^-69^) | 0.017 (0.66) | -0.064 (0.09) |
| Huextetitla 2003 | 250 kb | 0.55 (<10^-57^) | 0.089 (0.017) | -0.062 (0.09) |
| Huextetitla 2019 | 250 kb | 0.56 (<10^-57^) | 0.068 (0.07) | -0.024 (0.51) |
| *Santa Cruz 2020* | 500 kb | 0.62 (<10^-77^) | 0.04 (0.29) | -0.022 (0.56) |
| *Chapulhuacanito 2021* | 500 kb | 0.65 (<10^-85^) | 0.00002 (0.99) | -0.008 (0.84) |
| Chapulhuacanito 2003 | 500 kb | 0.62 (<10^-76^) | -0.022 (0.56) | -0.022 (0.55) |
| Chapulhuacanito 2006 | 500 kb | 0.64 (<10^-82^) | 0.017 (0.64) | 0.027 (0.47) |
| Chapulhuacanito 2017 | 500 kb | 0.64 (<10^-82^) | -0.04 (0.28) | 0.005 (0.89) |
| Huextetitla 2003 | 500 kb | 0.63 (<10^-79^) | 0.044 (0.24) | -0.011 (0.24) |
| Huextetitla 2019 | 500 kb | 0.61 (10^-73^) | 0.011 (0.77) | -0.011 (0.77) |
