# Supplementary material for: Swordtail fish hybrids reveal that genome evolution is surprisingly predictable after initial hybridization
Source: PLoS Biol. 2024 Aug 26;22(8):e3002742. doi: 10.1371/journal.pbio.3002742 (PMC11379403; doi:10.1371/journal.pbio.3002742)
Supplement: S7 Table — Since recombination rate has a strong effect on minor parent ancestry, and recombination rate is strongly correlated with functional elements in swordtails (see Text F in S1 File), analyses in genetic windows allows us to disentangle these effects. Windows were thinned so that on average on window per Mb was retained, and the same number of windows was retained for each analysis. Population names in italics indicate the focal samples primarily discussed in the main text. (DOCX) [file pbio.3002742.s008.docx]

**Table S7.** Analysis of correlations between minor parent ancestry and the number of coding or conserved basepairs in a window of a given genetic size. Since recombination rate has a strong effect on minor parent ancestry, and recombination rate is strongly correlated with functional elements in swordtails (see Text F in S1 File), analyses in genetic windows allows us to disentangle these effects. Windows were thinned so that on average on window per Mb was retained, and the same number of windows was retained for each analysis. Population names in italics indicate the focal samples primarily discussed in the main text.

| **Population** | **Window size** | **Correlation coding (p-value)** | **Correlation conserved (p-value)** |
| --- | --- | --- | --- |
| *Santa Cruz 2020* | 0.1 cM | -0.15 (0.0002) | -0.18 (<10^-8^) |
| *Chapulhuacanito 2021* | 0.1 cM | -0.21 (<10^-7^) | -0.27 (<10^-10^) |
| Chapulhuacanito 2003 | 0.1 cM | -0.19 (10^-5^) | -0.25 (<10^-8^) |
| Chapulhuacanito 2006 | 0.1 cM | -0.21 (<10^-6^) | -0.27 (10^-11^) |
| Chapulhuacanito 2017 | 0.1 cM | -0.22 (<10^-7^) | -0.29 (<10^-11^) |
| Huextetitla 2003 | 0.1 cM | -0.14 (0.0005) | -0.23 (<10^-8^) |
| Huextetitla 2019 | 0.1 cM | -0.11 (0.006) | -0.19 (<10^-5^) |
| *Santa Cruz 2020* | 0.25 cM | -0.23 (10^-7^) | -0.29 (<10^-10^) |
| *Chapulhuacanito 2021* | 0.25 cM | -0.26 (<10^-8^) | -0.29 (<10^-10^) |
| Chapulhuacanito 2003 | 0.25 cM | -0.26 (<10^-8^) | -0.28 (<10^-9^) |
| Chapulhuacanito 2006 | 0.25 cM | -0.26 (<10^-8^) | -0.30 (<10^-11^) |
| Chapulhuacanito 2017 | 0.25 cM | -0.27 (<10^-9^) | -0.28 (<10^-9^) |
| Huextetitla 2003 | 0.25 cM | -0.22 (<10^-6^) | -0.29 (<10^-10^) |
| Huextetitla 2019 | 0.25 cM | -0.19 (0.00002) | -0.25 (<10^-7^) |
| *Santa Cruz 2020* | 0.5 cM | -0.34 (<10^-19^) | -0.47 (<10^-29^) |
| *Chapulhuacanito 2021* | 0.5 cM | -0.38 (<10^-23^) | -0.42 (<10^-29^) |
| Chapulhuacanito 2003 | 0.5 cM | -0.38 (<10^-22^) | -0.42 (<10^-27^) |
| Chapulhuacanito 2006 | 0.5 cM | -0.38 (<10^-24^) | -0.41 (<10^-27^) |
| Chapulhuacanito 2017 | 0.5 cM | -0.39 (<10^-25^) | -0.42 (<10^-28^) |
| Huextetitla 2003 | 0.5 cM | -0.33 (<10^-18^) | -0.41(<10^-27^) |
| Huextetitla 2019 | 0.5 cM | -0.32 (<10^-16^) | -0.38 (<10^-23^) |
