# Supplementary material for: Swordtail fish hybrids reveal that genome evolution is surprisingly predictable after initial hybridization
Source: PLoS Biol. 2024 Aug 26;22(8):e3002742. doi: 10.1371/journal.pbio.3002742 (PMC11379403; doi:10.1371/journal.pbio.3002742)
Supplement: S8 Table — Analysis was conducted on thinned data, so that for each data set 1 window per Mb was retained, resulting in the same number of windows across comparisons. Both X. birchmanni × X. cortezi (Santa Cruz, Chapulhuacanito, Huextetitla) and X. birchmanni × X. malinche populations (Acuapa, Aguazarca, Tlatemaco) are included in this analysis. Note that the X. birchmanni × X. cortezi populations at Santa Cruz and Huextetitla occur in the same river system. All X. birchmanni × X. malinche populations occur in different river systems. (DOCX) [file pbio.3002742.s009.docx]

**Table S8.** Summary of local ancestry correlations across populations in windows of different sizes. Analysis was conducted on thinned data, so that for each data set one window per Mb was retained, resulting in the same number of windows across comparisons. Both *X. birchmanni* x *X. cortezi* (Santa Cruz, Chapulhuacanito, Huextetitla) and *X. birchmanni* x *X. malinche* populations (Acuapa, Aguazarca, Tlatemaco) are included in this analysis. Note that the *X. birchmanni* x *X. cortezi* populations at Santa Cruz and Huextetitla occur in the same river system. All *X. birchmanni* x *X. malinche* populations occur in different river systems.

| **Population 1** | **Population 2** | **Window size** | **Correlation in minor parent ancestry (Spearman’s ρ)** | **P-value** |
| --- | --- | --- | --- | --- |
| Santa Cruz 2020 | Chapulhuacanito 2021 | 500 kb | 0.86 | <10^-100^ |
| Santa Cruz 2020 | Huextetitla 2019 | 500 kb | 0.92 | <10^-100^ |
| Chapulhuacanito 2021 | Chapulhuacanito 2017 | 500 kb | 0.94 | <10^-100^ |
| Chapulhuacanito 2021 | Acuapa 2018 | 500 kb | 0.20 | <10^-7^ |
| Chapulhuacanito 2021 | Tlatemaco 2017 | 500 kb | 0.13 | 0.0007 |
| Chapulhuacanito 2021 | Aguazarca 2016 | 500 kb | 0.16 | <10^-4^ |
| Santa Cruz 2020 | Chapulhuacanito 2021 | 250 kb | 0.82 | <10^-100^ |
| Santa Cruz 2020 | Huextetitla 2019 | 250 kb | 0.90 | <10^-100^ |
| Chapulhuacanito 2021 | Chapulhuacanito 2017 | 250 kb | 0.92 | <10^-100^ |
| Chapulhuacanito 2021 | Acuapa 2018 | 250 kb | 0.19 | <10^-6^ |
| Chapulhuacanito 2021 | Tlatemaco 2017 | 250 kb | 0.10 | 0.012 |
| Chapulhuacanito 2021 | Aguazarca 2016 | 250 kb | 0.14 | 0.0003 |
| Santa Cruz 2020 | Chapulhuacanito 2021 | 100 kb | 0.79 | <10^-100^ |
| Santa Cruz 2020 | Huextetitla 2019 | 100 kb | 0.87 | <10^-100^ |
| Chapulhuacanito 2021 | Chapulhuacanito 2017 | 100 kb | 0.91 | <10^-100^ |
| Chapulhuacanito 2021 | Acuapa 2018 | 100 kb | 0.19 | <10^-5^ |
| Chapulhuacanito 2021 | Tlatemaco 2017 | 100 kb | 0.05 | 0.20 |
| Chapulhuacanito 2021 | Aguazarca 2016 | 100 kb | 0.14 | 0.0002 |
