# Supplementary material for: Swordtail fish hybrids reveal that genome evolution is surprisingly predictable after initial hybridization
Source: PLoS Biol. 2024 Aug 26;22(8):e3002742. doi: 10.1371/journal.pbio.3002742 (PMC11379403; doi:10.1371/journal.pbio.3002742)
Supplement: S9 Table — Binning into windows of varying genetic size allows us to partially control for the strong relationship between minor parent ancestry and recombination rate. We performed 2 analyses using a partial correlation approach, one comparing ancestry across populations while accounting for covariance due to the number of coding basepairs in a window, and a separate analysis comparing ancestry across populations while accounting for covariance due to the number of conserved basepairs in a window. Both X. birchmanni × X. cortezi (Santa Cruz, Chapulhuacanito, Huextetitla) and X. birchmanni × X. malinche populations (Acuapa, Aguazarca, Tlatemaco) are included in this analysis. Note that the X. birchmanni × X. cortezi populations at Santa Cruz and Huextetitla occur in the same river system. All X. birchmanni × X. malinche populations occur in different river systems. Analysis was conducted on thinned data, so that for each data set 1 window per 1.5 cMs was retained, resulting in the same number of windows analyzed across comparisons. (DOCX) [file pbio.3002742.s010.docx]

**Table S9.** Summary of local ancestry correlations across populations in windows of different genetic size. Binning into windows of varying genetic size allows us to partially control for the strong relationship between minor parent ancestry and recombination rate. We performed two analyses using a partial correlation approach, one comparing ancestry across populations while accounting for covariance due to the number of coding basepairs in a window, and a separate analysis comparing ancestry across populations while accounting for covariance due to the number of conserved basepairs in a window. Both *X. birchmanni* x *X. cortezi* (Santa Cruz, Chapulhuacanito, Huextetitla) and *X. birchmanni* x *X. malinche* populations (Acuapa, Aguazarca, Tlatemaco) are included in this analysis. Note that the *X. birchmanni* x *X. cortezi* populations at Santa Cruz and Huextetitla occur in the same river system. All *X. birchmanni* x *X. malinche* populations occur in different river systems. Analysis was conducted on thinned data, so that for each data set one window per 1.5 cMs was retained, resulting in the same number of windows analyzed across comparisons.

| **Population 1** | **Population 2** | **Window size (cM)** | **Partial correlation in minor parent ancestry (p-value)** | **Partial correlation with conserved basepair density (p-value)** |
| --- | --- | --- | --- | --- |
| Santa Cruz 2020 | Chapulhuacanito 2021 | 0.5 | 0.81  (<10^-100^) | -0.13  (<0.001) |
| Santa Cruz 2020 | Huextetitla 2019 | 0.5 | 0.93  (<10^-100^) | -0.17  (10^-5^) |
| Chapulhuacanito 2021 | Chapulhuacanito 2017 | 0.5 | 0.95  (<10^-100^) | -0.08  (0.036) |
| Chapulhuacanito 2021 | Acuapa 2018 | 0.5 | 0.22  (<10^-8^) | -0.42  (<10^-28^) |
| Chapulhuacanito 2021 | Tlatemaco 2017 | 0.5 | -0.05  (0.21) | -0.44  (<10^-31^) |
| Chapulhuacanito 2021 | Aguazarca 2016 | 0.5 | 0.10  (0.01) | -0.43  (<10^-30^) |
| Santa Cruz 2020 | Chapulhuacanito 2021 | 0.25 | 0.78  (<10^-100^) | -0.08  (0.03) |
| Santa Cruz 2020 | Huextetitla 2019 | 0.25 | 0.93  (<10^-100^) | -0.15  (0.0001) |
| Chapulhuacanito 2021 | Chapulhuacanito 2017 | 0.25 | 0.95  (<10^-100^) | -0.10  (0.016) |
| Chapulhuacanito 2021 | Acuapa 2018 | 0.25 | 0.18  (<10^-5^) | -0.37  (<10^-21^) |
| Chapulhuacanito 2021 | Tlatemaco 2017 | 0.25 | -0.02  (0.44) | -0.39  (<10^-23^) |
| Chapulhuacanito 2021 | Aguazarca 2016 | 0.25 | 0.06  (0.12) | -0.39  (<10^-22^) |
| Santa Cruz 2020 | Chapulhuacanito 2021 | 0.1 | 0.79  (<10^-100^) | -0.02  (0.58) |
| Santa Cruz 2020 | Huextetitla 2019 | 0.1 | 0.91  (<10^-100^) | -0.21  (<10^-7^) |
| Chapulhuacanito 2021 | Chapulhuacanito 2017 | 0.1 | 0.94  (<10^-100^) | -0.014  (0.71) |
| Chapulhuacanito 2021 | Acuapa 2018 | 0.1 | 0.18  (<10^-5^) | -0.31  (<10^-15^) |
| Chapulhuacanito 2021 | Tlatemaco 2017 | 0.1 | 0.038  (0.34) | -0.32  (<10^-16^) |
| Chapulhuacanito 2021 | Aguazarca 2016 | 0.1 | 0.13  (0.0008) | -0.32  (<10^-16^) |
