# Supplementary material for: Swordtail fish hybrids reveal that genome evolution is surprisingly predictable after initial hybridization
Source: PLoS Biol. 2024 Aug 26;22(8):e3002742. doi: 10.1371/journal.pbio.3002742 (PMC11379403; doi:10.1371/journal.pbio.3002742)
Supplement: S10 Table — Given the high correlations in minor parent ancestry observed between independently formed hybrid populations at Santa Cruz and Chapulhuacanito, we performed a series of analyses to exclude the role of potential artifacts in generating the signal we observe (see Methods). Thinned ancestry informative sites—ancestry informative sites were thinned before the HMM was run to better control for variation in power to infer ancestry across different regions of the genome. Intervals where ancestry informative sites occurred at higher than the median density were thinned to the median density and the HMM was re-run. Low power windows removed—windows with the fewest ancestry informative sites (lowest 5% quantile) were removed before analysis. Chromosome ends removed—windows within the last Mb of chromosomes, some of which overlapped with telomeric sequences, were removed. Repeats excluded—ancestry informative sites that overlapped with annotated repeats were excluded before analysis. All analyses were conducted on spatially thinned windows, so that for each data set 1 window per Mb was retained, resulting in the same number of windows across comparisons. (DOCX) [file pbio.3002742.s011.docx]

**Table S10.** Results of analyses controlling for potential artifacts. Given the high correlations in minor parent ancestry observed between independently formed hybrid populations at Santa Cruz and Chapulhuacanito, we performed a series of analyses to exclude the role of potential artifacts in generating the signal we observe (see Methods). Thinned ancestry informative sites – ancestry informative sites were thinned before the HMM was run to better control for variation in power to infer ancestry across different regions of the genome. Intervals where ancestry informative sites occurred at higher than the median density were thinned to the median density and the HMM was re-run. Low power windows removed – windows with the fewest ancestry informative sites (lowest 5% quantile) were removed before analysis. Chromosome ends removed – windows within the last Mb of chromosomes, some of which overlapped with telomeric sequences, were removed. Repeats excluded – ancestry informative sites that overlapped with annotated repeats were excluded before analysis. All analyses were conducted on spatially thinned windows, so that for each data set one window per Mb was retained, resulting in the same number of windows across comparisons.

| **Population 1** | **Population 2** | **Method** | **Window size** | **Spearman’s ρ (p-value)** |
| --- | --- | --- | --- | --- |
| Santa Cruz 2020 | Chapulhuacanito 2021 | Thinned ancestry informative sites | 500 kb | 0.86 (<10^-100^) |
| Santa Cruz 2020 | Chapulhuacanito 2021 | Thinned ancestry informative sites | 250 kb | 0.82 (<10^-100^) |
| Santa Cruz 2020 | Chapulhuacanito 2021 | Thinned ancestry informative sites | 100 kb | 0.79 (<10^-100^) |
| Santa Cruz 2020 | Chapulhuacanito 2021 | Population specific priors | 500 kb | 0.87 (<10^-100^) |
| Santa Cruz 2020 | Chapulhuacanito 2021 | Population specific priors | 250 kb | 0.84 (<10^-100^) |
| Santa Cruz 2020 | Chapulhuacanito 2021 | Population specific priors | 100 kb | 0.80 (<10^-100^) |
| Santa Cruz 2020 | Chapulhuacanito 2021 | Low power windows removed | 500 kb | 0.85 (<10^-100^) |
| Santa Cruz 2020 | Chapulhuacanito 2021 | Low power windows removed | 250 kb | 0.82 (<10^-100^) |
| Santa Cruz 2020 | Chapulhuacanito 2021 | Low power windows removed | 100 kb | 0.79 (<10^-100^) |
| Santa Cruz 2020 | Chapulhuacanito 2021 | Chromosome ends removed | 500 kb | 0.87 (<10^-100^) |
| Santa Cruz 2020 | Chapulhuacanito 2021 | Chromosome ends removed | 250 kb | 0.84 (<10^-100^) |
| Santa Cruz 2020 | Chapulhuacanito 2021 | Chromosome ends removed | 100 kb | 0.80 (<10^-100^) |
| Santa Cruz 2020 | Chapulhuacanito 2021 | Repeats excluded | 500 kb | 0.86 (<10^-100^) |
| Santa Cruz 2020 | Chapulhuacanito 2021 | Repeats excluded | 250 kb | 0.83 (<10^-100^) |
| Santa Cruz 2020 | Chapulhuacanito 2021 | Repeats excluded | 100 kb | 0.79 (<10^-20^) |
| Santa Cruz 2020 | Chapulhuacanito 2021 | Low power removed and chromosome ends removed | 500 kb | 0.87 (<10^-100^) |
| Santa Cruz 2020 | Chapulhuacanito 2021 | Low power removed and chromosome ends removed | 250 kb | 0.84 (<10^-100^) |
| Santa Cruz 2020 | Chapulhuacanito 2021 | Low power removed and chromosome ends removed | 100 kb | 0.80 (<10^-100^) |
