# Supplementary material for: Swordtail fish hybrids reveal that genome evolution is surprisingly predictable after initial hybridization
Source: PLoS Biol. 2024 Aug 26;22(8):e3002742. doi: 10.1371/journal.pbio.3002742 (PMC11379403; doi:10.1371/journal.pbio.3002742)
Supplement: S12 Table — cortezi populations and in time series data from Chapulhuacanito. (DOCX) [file pbio.3002742.s013.docx]

**Table S12.** Identity of mitochondrial haplotypes found in both *X. birchmanni* x *X. cortezi* populations and in time series data from Chapulhuacanito.

| **Population** | **Number of *X. cortezi* mitochondrial haplotypes** | **Number of *X. birchmanni* mitochondrial haplotypes** | **Frequency *X. cortezi* haplotype** |
| --- | --- | --- | --- |
| Santa Cruz 2020 | 242 | 0 | 100% |
| Chapulhuacanito 2003 | 7 | 0 | 100% |
| Chapulhuacanito 2006 | 19 | 0 | 100% |
| Chapulhuacanito 2017 | 18 | 1 | 95% |
| Chapulhuacanito 2021 | 69 | 0 | 100% |
