# Supplementary material for: Swordtail fish hybrids reveal that genome evolution is surprisingly predictable after initial hybridization
Source: PLoS Biol. 2024 Aug 26;22(8):e3002742. doi: 10.1371/journal.pbio.3002742 (PMC11379403; doi:10.1371/journal.pbio.3002742)
Supplement: S13 Table — (DOCX) [file pbio.3002742.s014.docx]

**Table S13.** Data from *Xiphophorus* species that was used to infer ancestral sequence states for LDhelmet.

| **Species** | **Data source** | **SRA accession** |
| --- | --- | --- |
| *X. malinche* | Schumer et al. 2018 | SRX3626998-SRX3626999 |
| *X. birchmanni* | Schumer et al. 2018 | SRX2486382-SRX3648154 |
| *X. cortezi* | Powell et al. 2020 | SRX7860174-SRX7860180 |
| *X. montezumae* | Schumer et al. 2016 | SRR3086791 |
| *X. continens* | Preising et al. 2022 | NA |
| *X. nezahualcoyotl* | Schumer et al. 2016 | SRR3086878; SRR3086631 |
| *X. nigrensis* | Preising et al. 2022 | NA |
| *X. multilineatus* | Preising et al. 2022 | NA |
| *X. variatus* | Powell et al. 2020 | SRR13982900 |
| *X. maculatus* | Schartl et al. 2013 | SRR7525605 |
| *X. hellerii* | Shen et al. 2016 | SRR7532852 |
