# Supplementary material for: Swordtail fish hybrids reveal that genome evolution is surprisingly predictable after initial hybridization
Source: PLoS Biol. 2024 Aug 26;22(8):e3002742. doi: 10.1371/journal.pbio.3002742 (PMC11379403; doi:10.1371/journal.pbio.3002742)
Supplement: S14 Table — (DOCX) [file pbio.3002742.s015.docx]

**Table S14.** Summary statistics input for ABCreg demographic inference analysis of Chapulhuacanito and Santa Cruz populations.

| **Whole genome** | | | | |
| --- | --- | --- | --- | --- |
| *Population* | *Median minor parent tract length* | *Average ancestry (proportion X. cortezi*) | *Standard deviation in genome-wide ancestry* | *Coefficient of variation in ancestry* |
| Chapulhuacanito 2021 | 69.6 kb | 0.76 | 0.018 | 0.024 |
| Santa Cruz 2020 | 33.9 kb | 0.85 | 0.031 | 0.036 |
| **Chromosome 2** | | | | |
| Chapulhuacanito 2021 | 63.6 kb | 0.80 | 0.053 | 0.067 |
| Santa Cruz 2020 | 30.0 kb | 0.86 | 0.067 | 0.078 |
