# Supplementary material for: Swordtail fish hybrids reveal that genome evolution is surprisingly predictable after initial hybridization
Source: PLoS Biol. 2024 Aug 26;22(8):e3002742. doi: 10.1371/journal.pbio.3002742 (PMC11379403; doi:10.1371/journal.pbio.3002742)
Supplement: S1 Fig — We obtain well-resolved posterior distributions for most parameters, including migration rate from the X. cortezi parent (A), migration rate from the X. birchmanni parent (B), initial admixture proportion (C), and generations since initial hybridization (D). We did not recover well-resolved posterior distributions for hybrid population size where we essentially recover the prior distribution (a uniform distribution from 2–10,000; not plotted—see Dryad repository doi:10.5061/dryad.qnk98sfq1). The dotted lines indicated the maximum a posteriori estimate for that parameter. The data underlying this figure can be found in Dryad repository doi:10.5061/dryad.qnk98sfq1. (PDF) [file pbio.3002742.s017.pdf]

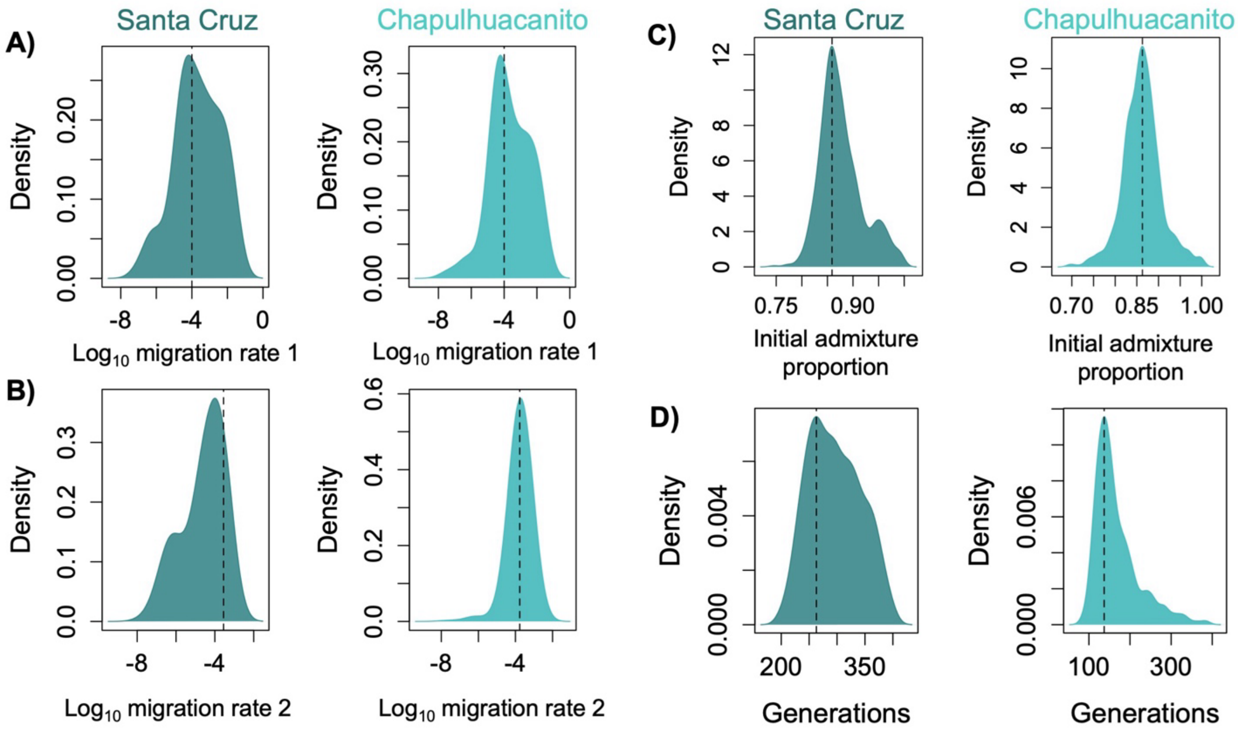

Fig. S1. Posterior distributions for demographic parameters from ABCreg analysis for Santa Cruz and Chapulhuacanito populations. We obtain well-resolved posterior distributions for most parameters, including migration rate from the *X. cortezi* parent (**A**), migration rate from the *X. birchmanni* parent (**B**), initial admixture proportion (**C**), and generations since initial hybridization (**D**). We did not recover well-resolved posterior distributions for hybrid population size where we essentially recover the prior distribution (a uniform distribution from 2-10,000; not plotted - see Dryad repository doi:10.5061/dryad.qnk98sfq1). The dotted lines indicated the maximum a posteriori estimate for that parameter. The data underlying this figure can be found in Dryad repository doi:10.5061/dryad.qnk98sfq1.
