# Supplementary material for: Swordtail fish hybrids reveal that genome evolution is surprisingly predictable after initial hybridization
Source: PLoS Biol. 2024 Aug 26;22(8):e3002742. doi: 10.1371/journal.pbio.3002742 (PMC11379403; doi:10.1371/journal.pbio.3002742)
Supplement: S2 Fig — Analyses in the main text show PCAs based on particular ancestry tracts (i.e., homozygous X. cortezi or homozygous X. birchmanni; Fig 1). This analysis includes all variant sites from all ancestry tracts. X. cortezi and X. birchmanni separate along PC1. Hybrids cluster close to X. cortezi along PC1, as expected given that they derive >70% of their genome from X. cortezi. We include pure X. birchmanni individuals found sympatrically with hybrids from Santa Cruz and Chapulhuacanito (xbirCHPL and xbirSTAC), as well as parental individuals from allopatric populations (xbirCOAC, xcorHUIC, and xcorPTHC). Hybrids from the Santa Cruz and Chapulhuacanito populations separate from each other in this analysis (xbirXcorSTAC and xbirXcorCHPL codes in the plot, respectively). The data underlying this figure can be found in Dryad repository doi:10.5061/dryad.qnk98sfq1. (PDF) [file pbio.3002742.s018.pdf]

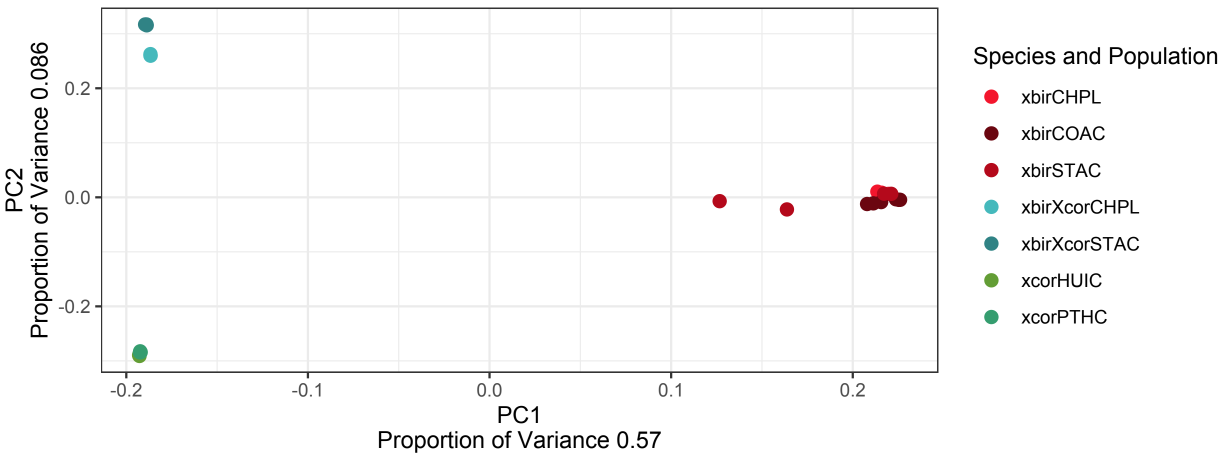

Fig. S2. Results of principal component analysis of individuals sequenced to high coverage from Santa Cruz and Chapulhuacanito as well as from allopatric parental populations including all variable sites across the genome. Analyses in the main text show PCAs based on particular ancestry tracts (i.e. homozygous *X. cortezi* or homozygous *X. birchmanni*; Fig. 1). This analysis includes all variant sites from all ancestry tracts. *X. cortezi* and *X. birchmanni* separate along PC1. Hybrids cluster close to *X. cortezi* along PC1, as expected given that they derive >70% of their genome from *X. cortezi*. We include pure *X. birchmanni* individuals found sympatrically with hybrids from Santa Cruz and Chapulhuacanito (xbirCHPL and xbirSTAC), as well as parental individuals from allopatric populations (xbirCOAC, xcorHUIC and xcorPTHC). Hybrids from the Santa Cruz and Chapulhuacanito populations separate from each other in this analysis (xbirXcorSTAC and xbirXcorCHPL codes in the plot respectively). The data underlying this figure can be found in Dryad repository doi:10.5061/dryad.qnk98sfq1.
