# Supplementary material for: Swordtail fish hybrids reveal that genome evolution is surprisingly predictable after initial hybridization
Source: PLoS Biol. 2024 Aug 26;22(8):e3002742. doi: 10.1371/journal.pbio.3002742 (PMC11379403; doi:10.1371/journal.pbio.3002742)
Supplement: S3 Fig — (A) We identified homozygous X. birchmanni ancestry tracts found in both Santa Cruz (STAC) and Chapulhuacanito (CHPL). We also subsampled these same regions from sympatric and allopatric pure parental individuals (X. birchmanni at COAC, CHPL, and STAC, X. cortezi at HUIC and PTHC). We found that Dxy to the reference X. birchmanni sequence was similar across the sequences analyzed for X. birchmanni tracts from hybrids and for those tracts from pure X. birchmanni individuals. As expected, X. cortezi populations had elevated Dxy to the X. birchmanni reference. (B) Similar patterns are observed for X. cortezi ancestry tracts analyzed with the same approach. X. birchmanni-derived regions had uniformly elevated Dxy compared to the X. cortezi reference (derived from the Puente de Huichihuayán or PTHC populations). Notably, homozygous X. cortezi ancestry tracts in hybrid individuals (X. cor × X. bir STAC and CHPL) have slightly but significantly higher Dxy to the X. cortezi reference sequence, hinting that the source population in both hybrid populations was somewhat diverged from the allopatric X. cortezi populations we sampled (consistent with expectations from geography). For both panels, colored points show the raw data and the black point and whiskers show the mean ± 2 standard errors of the mean. The data underlying this figure can be found in Dryad repository doi:10.5061/dryad.qnk98sfq1. (PDF) [file pbio.3002742.s019.pdf]

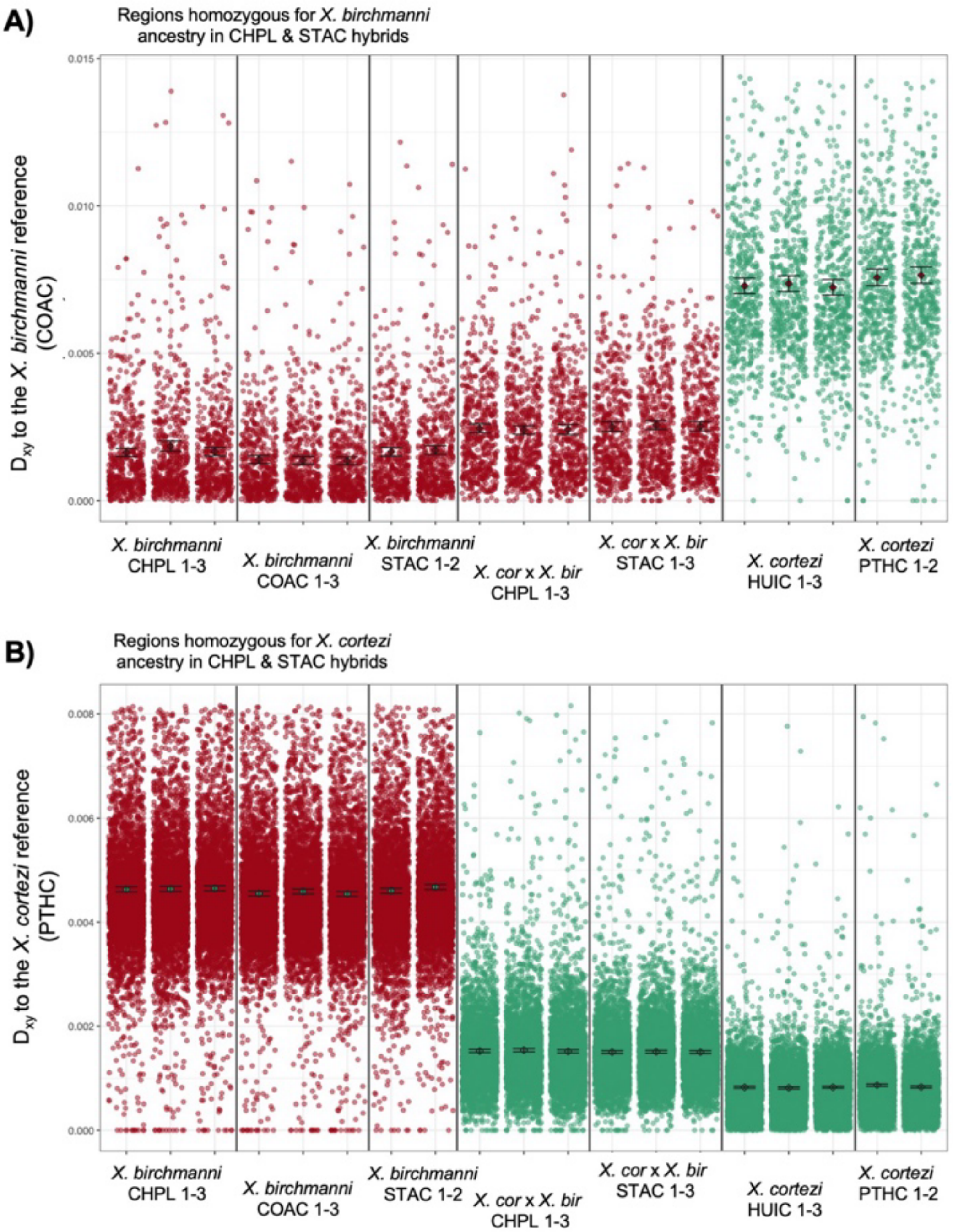

Fig. S3. Additional analyses of population genetic patterns in high coverage whole genome sequence data from Santa Cruz and Chapulhuacanito. **A**) We identified homozygous *X. birchmanni* ancestry tracts found in both Santa Cruz (STAC) and Chapulhuacanito (CHPL). We also subsampled these same regions from sympatric and allopatric pure parental individuals (*X. birchmanni* at COAC, CHPL, and STAC, *X. cortezi* at HUIC and PTHC). We found that Dxy to the reference *X. birchmanni* sequence was similar across the sequences analyzed for *X. birchmanni* tracts from hybrids and for those tracts from pure *X. birchmanni* individuals. As expected, *X. cortezi* populations had elevated Dxy to the *X. birchmanni* reference. **B**) Similar patterns are observed for *X. cortezi* ancestry tracts analyzed with the same approach. *X.*

birchmanni-derived regions had uniformly elevated Dxy compared to the *X. cortezi* reference (derived from the Puente de Huichihuayán or PTHC populations). Notably, homozygous *X. cortezi* ancestry tracts in hybrid individuals (*X. cor* x *X. bir* STAC and CHPL) have slightly but significantly higher Dxy to the *X. cortezi* reference sequence, hinting that the source population in both hybrid populations was somewhat diverged from the allopatric *X. cortezi* populations we sampled (consistent with expectations from geography). For both panels, colored points show the raw data and the black point and whiskers show the mean ± two standard errors of the mean. The data underlying this figure can be found in Dryad repository doi:10.5061/dryad.qnk98sfq1.
