# Supplementary material for: Swordtail fish hybrids reveal that genome evolution is surprisingly predictable after initial hybridization
Source: PLoS Biol. 2024 Aug 26;22(8):e3002742. doi: 10.1371/journal.pbio.3002742 (PMC11379403; doi:10.1371/journal.pbio.3002742)
Supplement: S4 Fig — In (A), we identified regions of the genome that were homozygous for X. birchmanni ancestry in the 3 deep-sequenced hybrids from Chapulhuacanito and the 3 from Santa Cruz. We subset these regions from the hybrids as well as allopatric parental populations and sympatric X. birchmanni individuals (X. birchmanni at COAC, CHPL, and STAC, X. cortezi at HUIC and PTHC). We find that pure X. birchmanni individuals have similar estimated π in these ancestry tracts, whereas Chapulhuacanito and to a lesser extent Santa Cruz have elevated π. In (B), we identified regions of the genome that were homozygous for X. cortezi ancestry in the 3 deep-sequenced hybrids from Chapulhuacanito and the 3 from Santa Cruz. We subset these regions from the hybrids as well as allopatric parental populations and sympatric X. birchmanni individuals. We found that although hybrids at Chapulhuacanito have similar estimated π to the X. cortezi parent in these ancestry tracts, hybrids at Santa Cruz have markedly lower estimated π. This may point to differences in the demographic history of Santa Cruz hybrids since the population formed, or in the X. cortezi parental population that contributed to the hybrids. For both panels, colored points show the raw data and the black point and whiskers shows the mean ± 2 standard errors of the mean. The data underlying this figure can be found in Dryad repository doi:10.5061/dryad.qnk98sfq1. (PDF) [file pbio.3002742.s020.pdf]

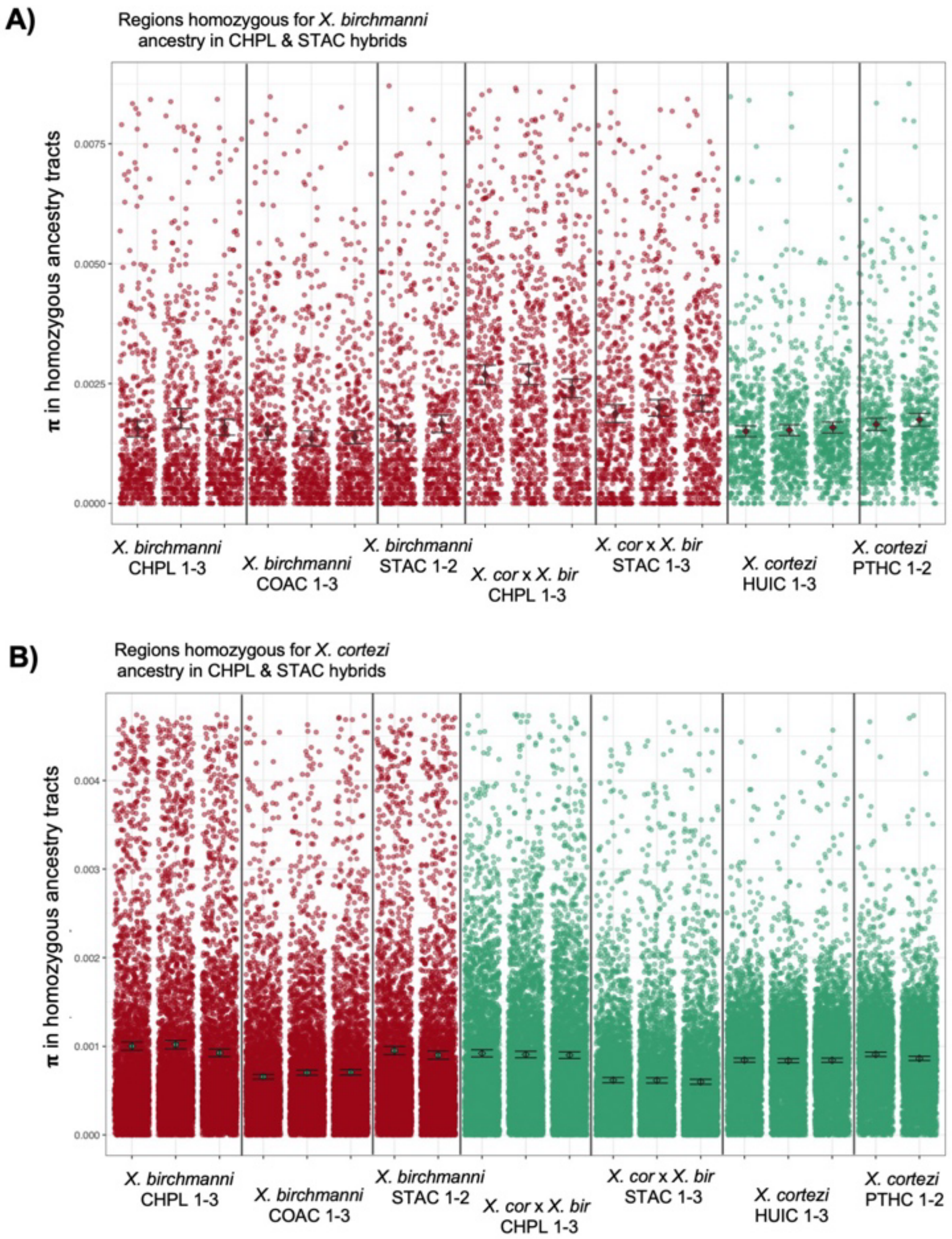

Fig. S4. Additional analyses of population genetic patterns in high coverage whole genome sequence data from Santa Cruz and Chapulhuacanito focusing on patterns of nucleotide diversity. In **A)**, we identified regions of the genome that were homozygous for *X. birchmanni* ancestry in the three deep-sequenced hybrids from Chapulhuacanito and the three from Santa Cruz. We subset these regions from the hybrids as well as allopatric parental populations and sympatric *X. birchmanni* individuals (*X. birchmanni* at COAC, CHPL, and STAC, *X. cortezi* at

HUIC and PTHC). We find that pure *X. birchmanni* individuals have similar estimated π in these ancestry tracts, whereas Chapulhuacanito and to a lesser extent Santa Cruz have elevated π. In **B)**, we identified regions of the genome that were homozygous for *X. cortezi* ancestry in the three deep-sequenced hybrids from Chapulhuacanito and the three from Santa Cruz. We subset these regions from the hybrids as well as allopatric parental populations and sympatric *X. birchmanni* individuals. We found that although hybrids at Chapulhuacanito have similar estimated π to the *X. cortezi* parent in these ancestry tracts, hybrids at Santa Cruz have markedly lower estimated π. This may point to differences in the demographic history of Santa Cruz hybrids since the population formed, or in the *X. cortezi* parental population that contributed to the hybrids. For both panels, colored points show the raw data and the black point and whiskers shows the mean ± two standard errors of the mean. The data underlying this figure can be found in Dryad repository doi:10.5061/dryad.qnk98sfq1.
