# Supplementary material for: Swordtail fish hybrids reveal that genome evolution is surprisingly predictable after initial hybridization
Source: PLoS Biol. 2024 Aug 26;22(8):e3002742. doi: 10.1371/journal.pbio.3002742 (PMC11379403; doi:10.1371/journal.pbio.3002742)
Supplement: S5 Fig — In homozygous X. cortezi ancestry tracts identified in high-coverage individuals, we observe high “mismatch” in homozygous alleles across the 2 hybrid populations relative to the values we observe within populations (Fig 1G; see Methods). We performed simulations to explore whether the level of mismatch observed across the 2 hybrid populations is compatible with a partially shared demographic history in the 2 populations (see Text C in S1 File for details). We modeled a population under demographic parameters inferred from previous simulations for Santa Cruz and Chapulhuacanito except that we modeled 10 (A), 50 (B), or 100 (C) generations of shared evolution. For each scenario, we repeated simulations for a total of 100 replicates (gray distributions) and compared mismatch values in these simulations to the mismatch value we calculated for the empirical data (see also Fig 1G). The simulated scenarios of partially shared evolutionary history overlapped with the empirical mismatch statistics for within-population analysis of Santa Cruz (green line) and Chapulhuacanito (blue line) but not the observed value for the between population mismatch statistics (yellow dashed line). This suggests that scenarios of non-independence in the 2 hybrid populations are not consistent with our data. The data underlying this figure can be found in Dryad repository doi:10.5061/dryad.qnk98sfq1. (PDF) [file pbio.3002742.s021.pdf]

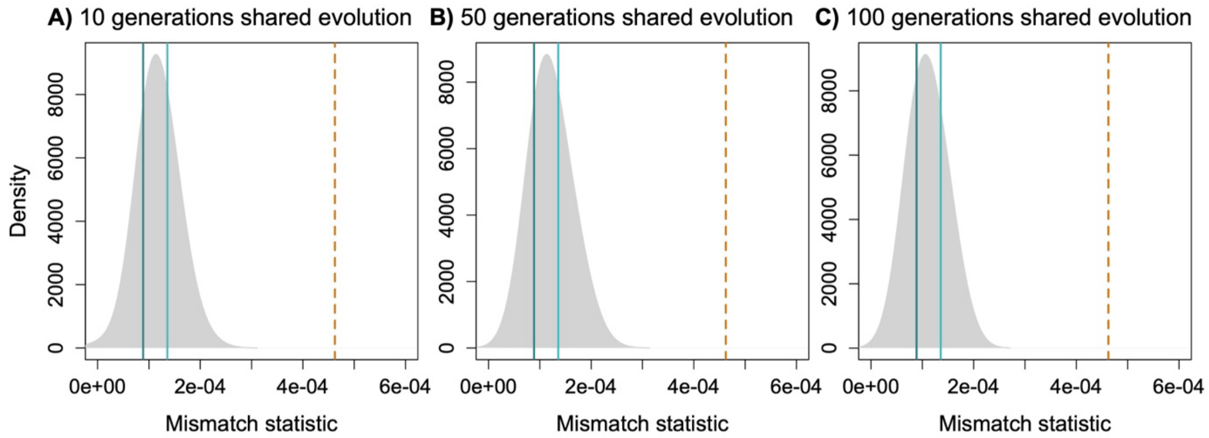

Fig. S5. Comparisons of simulated versus observed mismatch statistics. In homozygous *X. cortezi* ancestry tracts identified in high coverage individuals, we observe high "mismatch" in homozygous alleles across the two hybrid populations relative to the values we observe within populations (Fig. 1G; see Methods). We performed simulations to explore whether the level of mismatch observed across the two hybrid populations is compatible with a partially shared demographic history in the two populations (see Text C in S1 File for details). We modeled a population under demographic parameters inferred from previous simulations for Santa Cruz and Chapulhuacanito except that we modeled 10 (**A**), 50 (**B**), or 100 (**C**) generations of shared evolution. For each scenario, we repeated simulations for a total of 100 replicates (gray distributions) and compared mismatch values in these simulations to the mismatch value we calculated for the empirical data (see also Fig. 1G). The simulated scenarios of partially shared evolutionary history overlapped with the empirical mismatch statistics for within-population analysis of Santa Cruz (green line) and Chapulhuacanito (blue line) but not the observed value for the between population mismatch statistics (yellow dashed line). This suggests that scenarios of non-independence in the two hybrid populations are not consistent with our data. The data underlying this figure can be found in Dryad repository doi:10.5061/dryad.qnk98sfq1.
