# Supplementary material for: Swordtail fish hybrids reveal that genome evolution is surprisingly predictable after initial hybridization
Source: PLoS Biol. 2024 Aug 26;22(8):e3002742. doi: 10.1371/journal.pbio.3002742 (PMC11379403; doi:10.1371/journal.pbio.3002742)
Supplement: S6 Fig — Analysis was performed with GCTA (see Methods). (A) Analysis of X. cortezi ancestry tracts in 3 hybrids from the Santa Cruz (Hyb-STAC) population and 3 hybrids from Chapulhuacanito (Hyb-CHPL) population along with the same regions sampled from individuals from the 2 X. cortezi parental populations, Huichihuayán (HUIC) and Puente de Huichihuayán (PTHC). Cooler colors indicate lower genetic relatedness than the average in the data set and warmer colors indicate higher genetic relatedness than the average in the data set. Overall, we do not see evidence of genetic relatedness across hybrid populations but substantial evidence of relatedness within populations, with higher relatedness in comparisons between Santa Cruz individuals. High relatedness between pure X. cortezi in Huichihuayán and Puente de Huichihuayán is not surprising since these populations occur on the same river. (B) Results of the same analysis, but for ancestry tracts that were homozygous for X. birchmanni in the 3 high-coverage hybrids from Santa Cruz (Hyb-STAC) and Chapulhuacanito (Hyb-CHPL), as well as the same regions sampled from pure X. birchmanni from the Río Santa Cruz (Xbir-STAC, Xbir-HUEX) and Chapulhuacanito (Xbir-CHPL) and from a pure X. birchmanni source population (Xbir-COAC). Overall, the results for hybrid populations are concordant with the analysis in A, with evidence of relatedness within but not between hybrid populations. However, we do observe more relatedness across X. birchmanni individuals from different rivers than we expected a priori. See Text E in S1 File for a discussion of these results. The data underlying this figure can be found in Dryad repository doi:10.5061/dryad.qnk98sfq1. (PDF) [file pbio.3002742.s022.pdf]

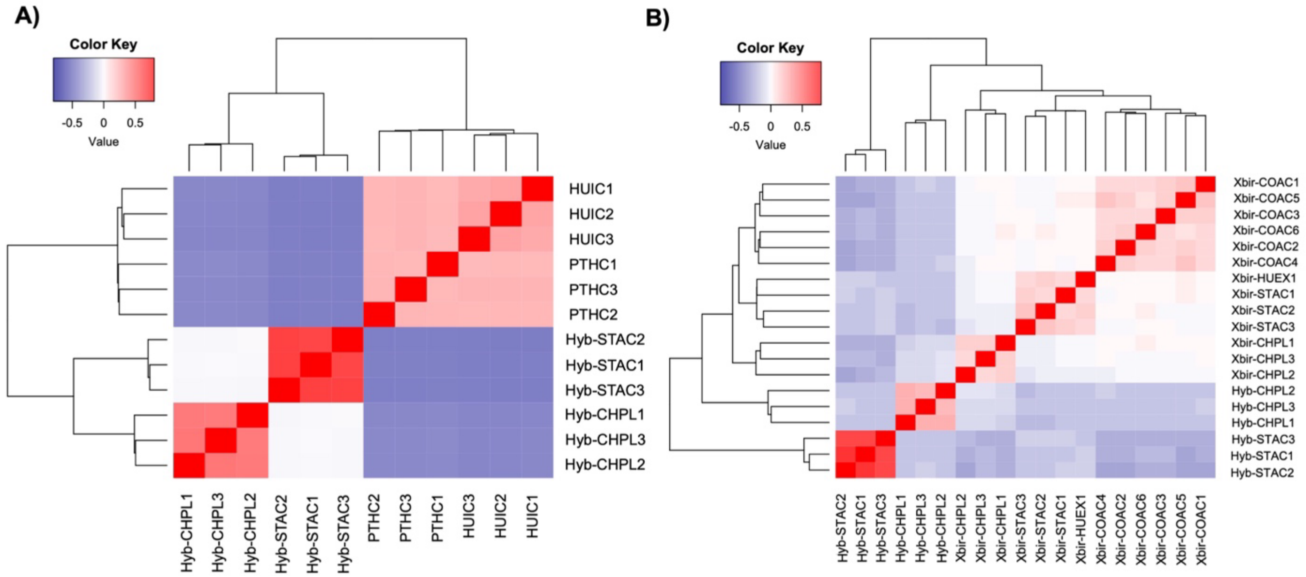

Fig. S6. Heatmap of the genetic relatedness matrices across different subsets of samples and regions of the genome. Analysis was performed with GCTA (see Methods). **A)** Analysis of *X. cortezi* ancestry tracts in three hybrids from the Santa Cruz (Hyb-STAC) population and three hybrids from Chapulhuacanito (Hyb-CHPL) population along with the same regions sampled from individuals from the two *X. cortezi* parental populations, Huichihuayán (HUIC) and Puente de Huichihuayán (PTHC). Cooler colors indicate lower genetic relatedness than the average in the dataset and warmer colors indicate higher genetic relatedness than the average in the dataset. Overall, we do not see evidence of genetic relatedness across hybrid populations but substantial evidence of relatedness within populations, with higher relatedness in comparisons between Santa Cruz individuals. High relatedness between pure *X. cortezi* in Huichihuayán and Puente de Huichihuayán is not surprising since these populations occur on the same river. **B)** Results of the same analysis, but for ancestry tracts that were homozygous for *X. birchmanni* in the three high-coverage hybrids from Santa Cruz (Hyb-STAC) and Chapulhuacanito (Hyb-CHPL), as well as the same regions sampled from pure *X. birchmanni* from the Río Santa Cruz (Xbir-STAC, Xbir-HUEX) and Chapulhuacanito (Xbir-CHPL) and from a pure *X. birchmanni* source population (Xbir-COAC). Overall, the results for hybrid populations are concordant with the analysis in **A**, with evidence of relatedness within but not between hybrid populations. However, we do observe more relatedness across *X. birchmanni* individuals from different rivers than we expected *a priori*. See Text E in S1 File for a discussion of these results. The data underlying this figure can be found in Dryad repository doi:10.5061/dryad.qnk98sfq1.
