# Supplementary material for: Swordtail fish hybrids reveal that genome evolution is surprisingly predictable after initial hybridization
Source: PLoS Biol. 2024 Aug 26;22(8):e3002742. doi: 10.1371/journal.pbio.3002742 (PMC11379403; doi:10.1371/journal.pbio.3002742)
Supplement: S7 Fig — (A) The vast majority of IBD tracts identified by our analysis corresponded to IBD tracts shared between individuals sampled from the same geographical location. These also represented the longest IBD tracts on average. (B) Many fewer IBD tracts were identified between individuals originating from different geographical locations, and these tracts were shorter on average. The majority of these “between-population” tracts were attributable to tracts shared with pure X. birchmanni or pure X. cortezi populations (see Text D in S1 File, S4 Table). (C) Very few IBD tracts were identified between the 2 hybrid populations, and these were on average approximately 20 kb in length. This suggests that there is little detectable genetic exchange or shared demographic history between these populations. Moreover, since this length is smaller than the typical ancestry tract in either population, these tracts likely are identical-by-descent due to events that preceded hybridization between X. birchmanni and X. cortezi in either hybrid population. Red dashed line shows median of each distribution. The data underlying this figure can be found in Dryad repository doi:10.5061/dryad.qnk98sfq1. (PDF) [file pbio.3002742.s023.pdf]

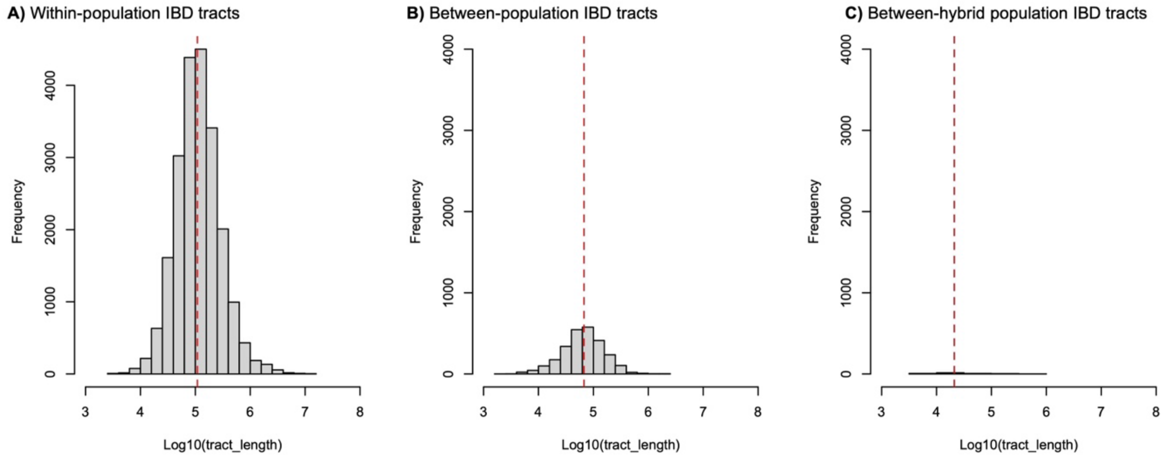

Fig. S7. Frequency and length of IBD tracts inferred via IBDseq analysis for different comparisons of data from high coverage individuals. **A**) The vast majority of IBD tracts identified by our analysis corresponded to IBD tracts shared between individuals sampled from the same geographical location. These also represented the longest IBD tracts on average. **B**) Many fewer IBD tracts were identified between individuals originating from different geographical locations, and these tracts were shorter on average. The majority of these "between-population" tracts were attributable to tracts shared with pure *X. birchmanni* or pure *X. cortezi* populations (see Text D in S1 File, Table S4). **C**) Very few IBD tracts were identified between the two hybrid populations, and these were on average ~20 kb in length. This suggests that there is little detectable genetic exchange or shared demographic history between these populations. Moreover, since this length is smaller than the typical ancestry tract in either population, these tracts likely are identical-by-descent due to events that preceded hybridization between *X. birchmanni* and *X. cortezi* in either hybrid population. Red dashed line shows median of each distribution. The data underlying this figure can be found in Dryad repository doi:10.5061/dryad.qnk98sfq1.
