# Supplementary material for: Swordtail fish hybrids reveal that genome evolution is surprisingly predictable after initial hybridization
Source: PLoS Biol. 2024 Aug 26;22(8):e3002742. doi: 10.1371/journal.pbio.3002742 (PMC11379403; doi:10.1371/journal.pbio.3002742)
Supplement: S8 Fig — Plots show the (A) Chapulhuacanito (CHPL) or (B) Santa Cruz (STAC) hybrid populations. Red dashed line shows median of each distribution. For other comparisons within and between populations, see S4 Table. The data underlying this figure can be found in Dryad repository doi:10.5061/dryad.qnk98sfq1. (PDF) [file pbio.3002742.s024.pdf]

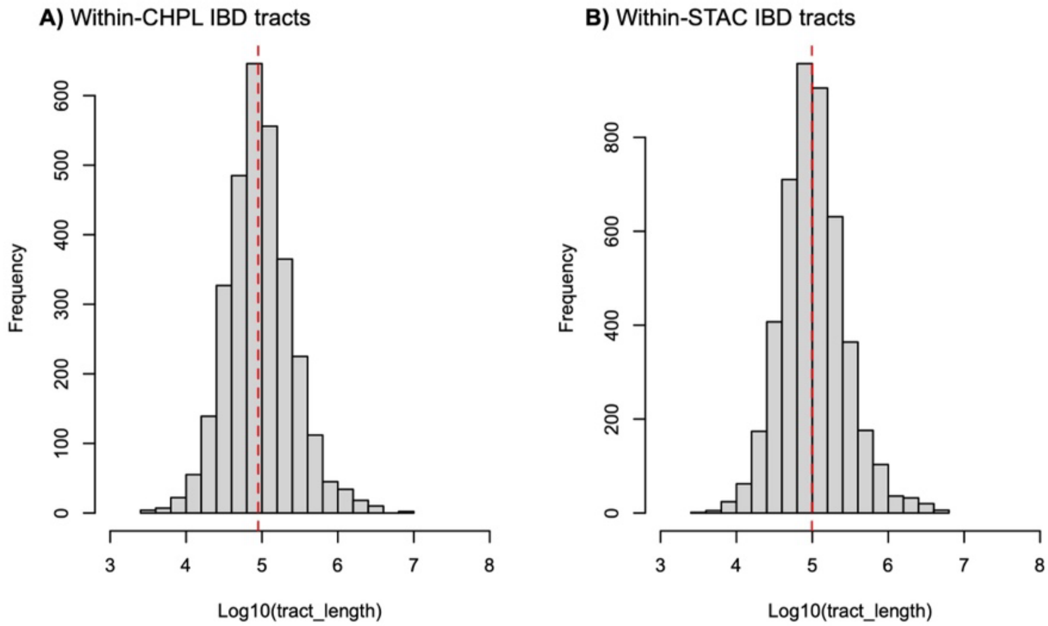

Fig. S8. Frequency and length of IBD tracts inferred via IBDseq analysis within hybrid populations. Plots show the **A)** Chapulhuacanito (CHPL) or **B)** Santa Cruz (STAC) hybrid populations. Red dashed line shows median of each distribution. For other comparisons within and between populations, see Table S4. The data underlying this figure can be found in Dryad repository doi:10.5061/dryad.qnk98sfq1.
