# Supplementary material for: Swordtail fish hybrids reveal that genome evolution is surprisingly predictable after initial hybridization
Source: PLoS Biol. 2024 Aug 26;22(8):e3002742. doi: 10.1371/journal.pbio.3002742 (PMC11379403; doi:10.1371/journal.pbio.3002742)
Supplement: S9 Fig — Since Xiphophorus species lack a PRDM9 ortholog that is active in meiosis and recombine primarily at functional elements that are largely conserved between species (S10 Fig), we expected their recombination maps to be conserved as well. Comparison of local recombination rates across a range of window sizes (A–C) confirms this prediction (A–Spearman’s ρ = 0.55; B–Spearman’s ρ = 0.57; C–Spearman’s ρ = 0.62). Given that previous simulations of the X. birchmanni recombination map indicated that the expected correlations between the true recombination map and the inferred LD-based recombination map were approximately 0.65 in 50 kb windows [1], this suggests that the 2 maps may be nearly identical. See Text F in S1 File for more detail. The data underlying this figure can be found in Dryad repository doi:10.5061/dryad.qnk98sfq1. (PDF) [file pbio.3002742.s025.pdf]

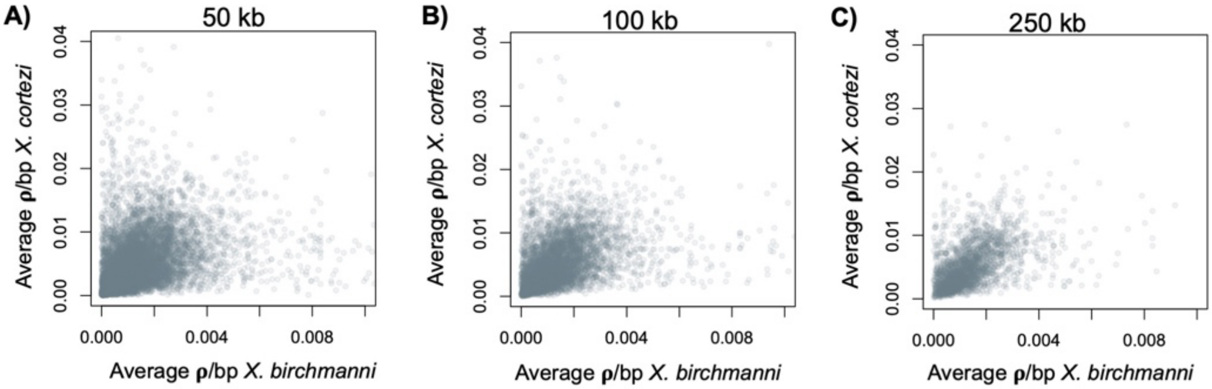

Fig. S9. Comparisons of inferred local recombination rates in *X. birchmanni* and *X. cortezi*. Since *Xiphophorus* species lack a PRDM9 ortholog that is active in meiosis and recombine primarily at functional elements that are largely conserved between species (Fig. S10), we expected their recombination maps to be conserved as well. Comparison of local recombination rates across a range of window sizes (**A-C**) confirms this prediction (**A** – Spearman's $\rho = 0.55$; **B** – Spearman's $\rho = 0.57$; **C** – Spearman's $\rho = 0.62$). Given that previous simulations of the *X. birchmanni* recombination map indicated that the expected correlations between the true recombination map and the inferred LD-based recombination map were approximately 0.65 in 50 kb windows [1], this suggests that the two maps may be nearly identical. See Text F in S1 File for more detail. The data underlying this figure can be found in Dryad repository doi:10.5061/dryad.qnk98sfq1.

References

1. Schumer M, Xu C, Powell DL, Durvasula A, Skov L, Holland C, et al. Natural selection interacts with recombination to shape the evolution of hybrid genomes. Science. 2018;360: 656. doi:10.1126/science.aar3684
