# Supplementary material for: Swordtail fish hybrids reveal that genome evolution is surprisingly predictable after initial hybridization
Source: PLoS Biol. 2024 Aug 26;22(8):e3002742. doi: 10.1371/journal.pbio.3002742 (PMC11379403; doi:10.1371/journal.pbio.3002742)
Supplement: S10 Fig — Average recombination rate (ρ/bp) in sliding 5 kb windows as a function of distance of that window from the nearest transcriptional start site (TSS) or H3K4me3 peak in testis tissue in basepairs in X. birchmanni (A, C) and X. cortezi (B, D). In both species, average recombination rates peak near the TSS (A, B), as frequently observed in species that lack a PRDM9 ortholog active in specifying recombination hotspots. This pattern is thought to be driven by recombination machinery defaulting to the locations of existing H3K4me3 marks in the absence of PRDM9-driven H3K4me3 marks. Indeed, we see that both species have elevated recombination near shared H3K4me3 identified in X. birchmanni testis samples (data reanalyzed from [1]; C, D). Gray lines show individual replicates bootstrap resampling the data, with a total of 500 replicates plotted. Blue line shows the average across simulations. The data underlying this figure can be found in Dryad repository doi:10.5061/dryad.qnk98sfq1. (PDF) [file pbio.3002742.s026.pdf]

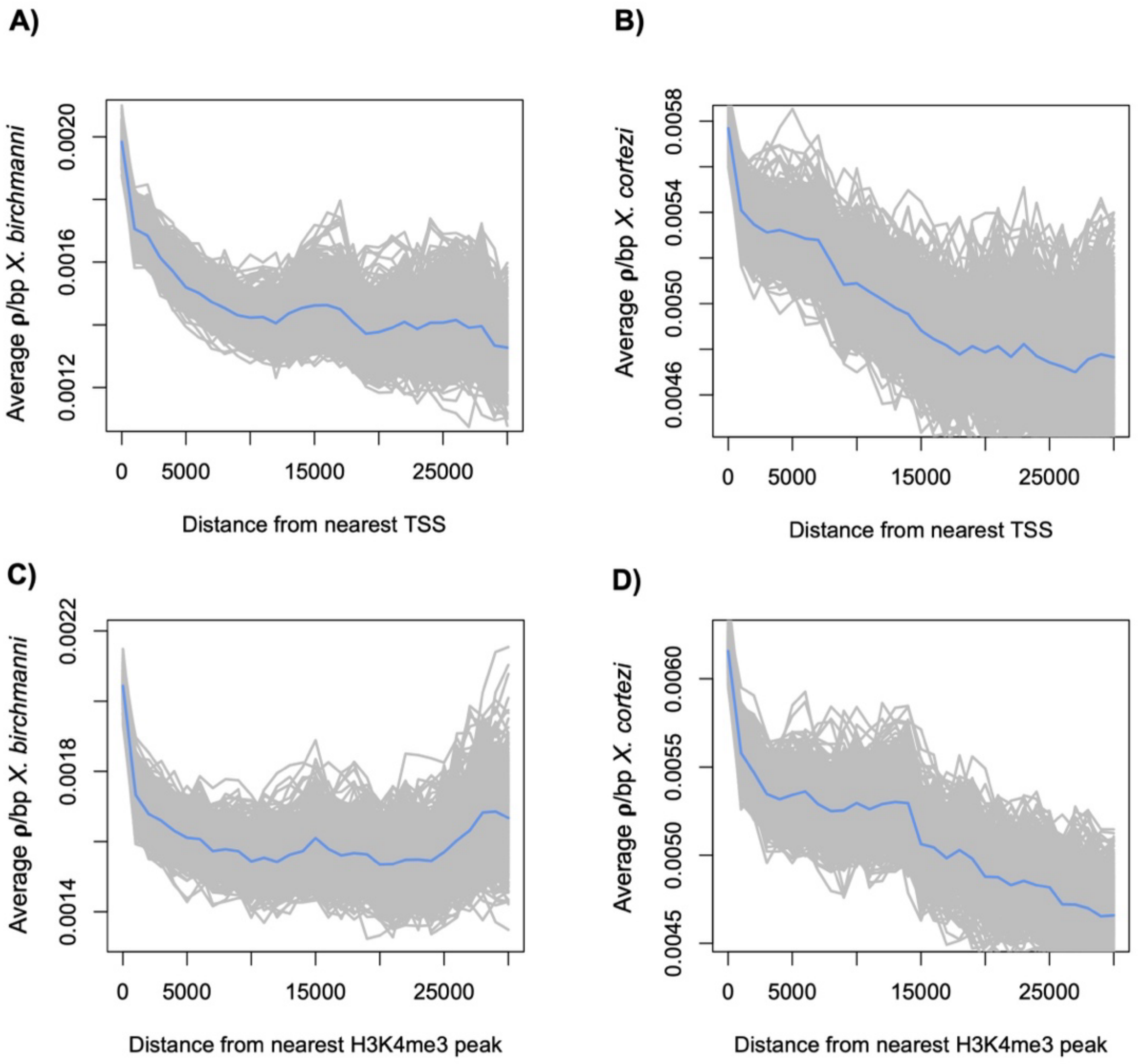

Fig. S10. Recombination rate as a function of distance to certain genomic elements. Average recombination rate (ρ/bp) in sliding 5 kb windows as a function of distance of that window from the nearest transcriptional start site (TSS) or H3K4me3 peak in testis tissue in basepairs in *X. birchmanni* (**A**, **C**) and *X. cortezi* (**B**, **D**). In both species, average recombination rates peak near the TSS (**A**, **B**), as frequently observed in species that lack a PRDM9 ortholog active in specifying recombination hotspots. This pattern is thought to be driven by recombination machinery defaulting to the locations of existing H3K4me3 marks in the absence of PRDM9-driven H3K4me3 marks. Indeed, we see that both species have elevated recombination near shared H3K4me3 identified in *X. birchmanni* testis samples (data reanalyzed from [1]; **C**, **D**). Gray lines show individual replicates bootstrap resampling the data, with a total of 500 replicates plotted. Blue line shows the average across simulations. The data underlying this figure can be found in Dryad repository doi:10.5061/dryad.qnk98sfq1.

References

1. Baker Z, Schumer M, Haba Y, Bashkirova L, Holland C, Rosenthal GG, et al. Repeated losses of PRDM9-directed recombination despite the conservation of PRDM9 across vertebrates. In: eLife [Internet]. 6 Jun 2017 [cited 23 Jul 2019]. doi:10.7554/eLife.24133
