# Supplementary material for: Swordtail fish hybrids reveal that genome evolution is surprisingly predictable after initial hybridization
Source: PLoS Biol. 2024 Aug 26;22(8):e3002742. doi: 10.1371/journal.pbio.3002742 (PMC11379403; doi:10.1371/journal.pbio.3002742)
Supplement: S11 Fig — The y-axis shows the average R2 value in sliding 5 kb windows and the x-axis shows the physical distance between ancestry informative sites in the focal window. Both populations asymptote to background levels of admixture LD by approximately 500 kb, although we thin our data by 1 Mb to be conservative (see Methods). We note also that there is an excess of admixture linkage disequilibrium in both populations where the minimum average R2 greatly exceeds one over the number of sampled individuals, particularly in the Santa Cruz population. This is suggestive of population structure, a recent pulse of migration, or assortative mating in these populations (see [68] for data on assortative mating in Santa Cruz). The data underlying this figure can be found in Dryad repository doi:10.5061/dryad.qnk98sfq1. (PDF) [file pbio.3002742.s027.pdf]

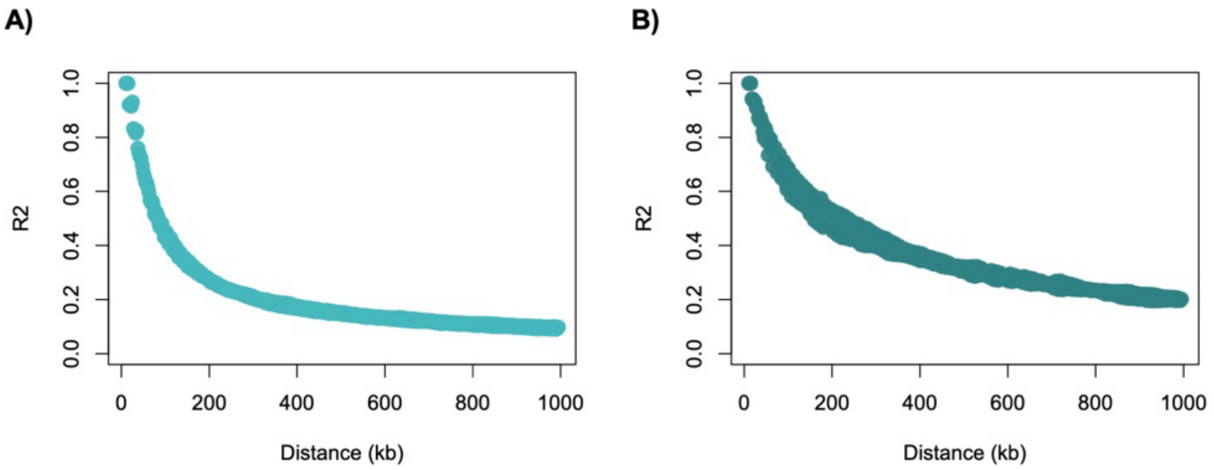

Fig. S11. Average decay of admixture linkage disequilibrium among hybrid individuals from the Chapulhuacanito (left) and Santa Cruz (right) hybrid populations. The y-axis shows the average R^2 value in sliding 5 kb windows and the x-axis shows the physical distance between ancestry informative sites in the focal window. Both populations asymptote to background levels of admixture LD by approximately 500 kb, although we thin our data by 1 Mb to be conservative (see Methods). We note also that there is an excess of admixture linkage disequilibrium in both populations where the minimum average R^2 greatly exceeds one over the number of sampled individuals, particularly in the Santa Cruz population. This is suggestive of population structure, a recent pulse of migration, or assortative mating in these populations (see [1] for data on assortative mating in Santa Cruz). The data underlying this figure can be found in Dryad repository doi:10.5061/dryad.qnk98sfq1.

References

Powell DL, Moran BM, Kim BY, Banerjee SM, Aguillon SM, Fascinetto-Zago P, et al. Two new hybrid populations expand the swordtail hybridization model system. Evolution. 2021;75: 2524–2539. doi:10.1111/evo.14337
