# Supplementary material for: Swordtail fish hybrids reveal that genome evolution is surprisingly predictable after initial hybridization
Source: PLoS Biol. 2024 Aug 26;22(8):e3002742. doi: 10.1371/journal.pbio.3002742 (PMC11379403; doi:10.1371/journal.pbio.3002742)
Supplement: S12 Fig — Average minor parent ancestry for matched regions of the genome is plotted on the x and y axes in 100 kb windows. (A) Average ancestry in Chapulhuacanito (CHPL) population samples from 2021 are plotted against average ancestry in the same windows from the Santa Cruz (STAC) population sampled in 2020. Correlations in local ancestry are high, but windows with high minor parent ancestry in Chapulhuacanito and low minor parent ancestry in Santa Cruz (and vice versa) can be visualized in the plot. By contrast, local ancestry comparisons from the same population but different years (B) or 2 nearby populations in the same river drainage (C) are remarkably concordant. D and E show results from subsampling 20 individuals from the same population and sampling year. Again, correlations between individuals sampled from the same population (D–Chapulhuacanito, E–Santa Cruz) greatly exceed observed correlations between drainages (A). The data underlying this figure can be found in Dryad repository doi:10.5061/dryad.qnk98sfq1. (PDF) [file pbio.3002742.s028.pdf]

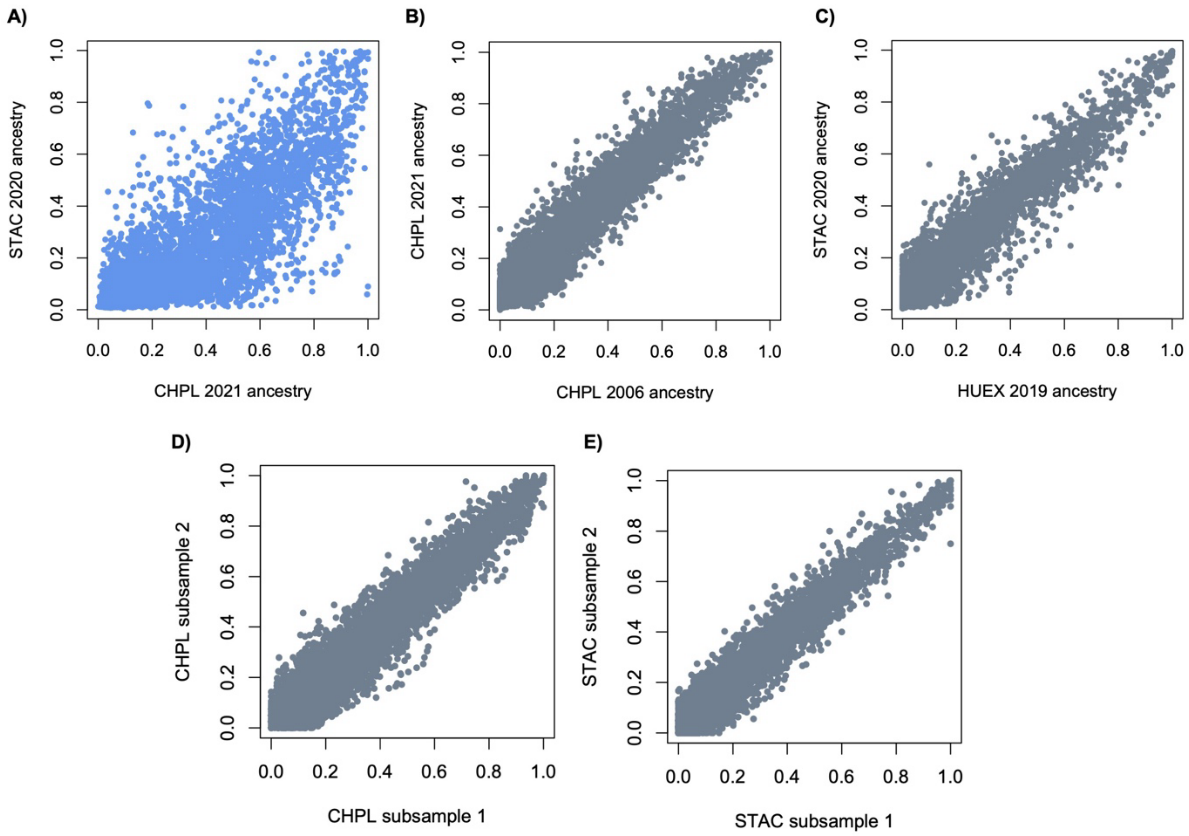

Fig. S12. Visualization of cross-population versus within population (or river) correlations in minor parent ancestry. Average minor parent ancestry for matched regions of the genome is plotted on the x and y axes in 100 kb windows. **A**) Average ancestry in Chapulhuacanito (CHPL) population samples from 2021 are plotted against average ancestry in the same windows from the Santa Cruz (STAC) population sampled in 2020. Correlations in local ancestry are high, but windows with high minor parent ancestry in Chapulhuacanito and low minor parent ancestry in Santa Cruz (and vice versa) can be visualized in the plot. By contrast, local ancestry comparisons from the same population but different years (**B**) or two nearby populations in the same river drainage (**C**) are remarkably concordant. **D** and **E** show results from subsampling twenty individuals from the same population and sampling year. Again, correlations between individuals sampled from the same population (**D** – Chapulhuacanito, **E** – Santa Cruz), greatly exceed observed correlations between drainages (**A**). The data underlying this figure can be found in Dryad repository doi:10.5061/dryad.qnk98sfq1.
