# Supplementary material for: Swordtail fish hybrids reveal that genome evolution is surprisingly predictable after initial hybridization
Source: PLoS Biol. 2024 Aug 26;22(8):e3002742. doi: 10.1371/journal.pbio.3002742 (PMC11379403; doi:10.1371/journal.pbio.3002742)
Supplement: S13 Fig — (A) Analyses of genome-wide ancestry in allopatric X. birchmanni individuals demonstrate that ancestryinfer correctly infers that these individuals are homozygous for X. birchmanni ancestry across the genome. (B) This result can also be observed by examining local ancestry in the same individuals (3 representative individuals plotted here), where entire chromosomes are inferred to have 2 X. birchmanni-derived and no X. cortezi-derived haplotypes. (C) Similarly, ancestry inference in allopatric X. cortezi individuals is accurate, with all individuals inferred to be homozygous X. cortezi throughout the genome. (D) This result can also be observed by examining local ancestry in the same individuals (3 representative individuals plotted here), where entire chromosomes are inferred to have 2 X. cortezi derived and no X. birchmanni-derived haplotypes. The data underlying this figure can be found in Dryad repository doi:10.5061/dryad.qnk98sfq1. (PDF) [file pbio.3002742.s029.pdf]

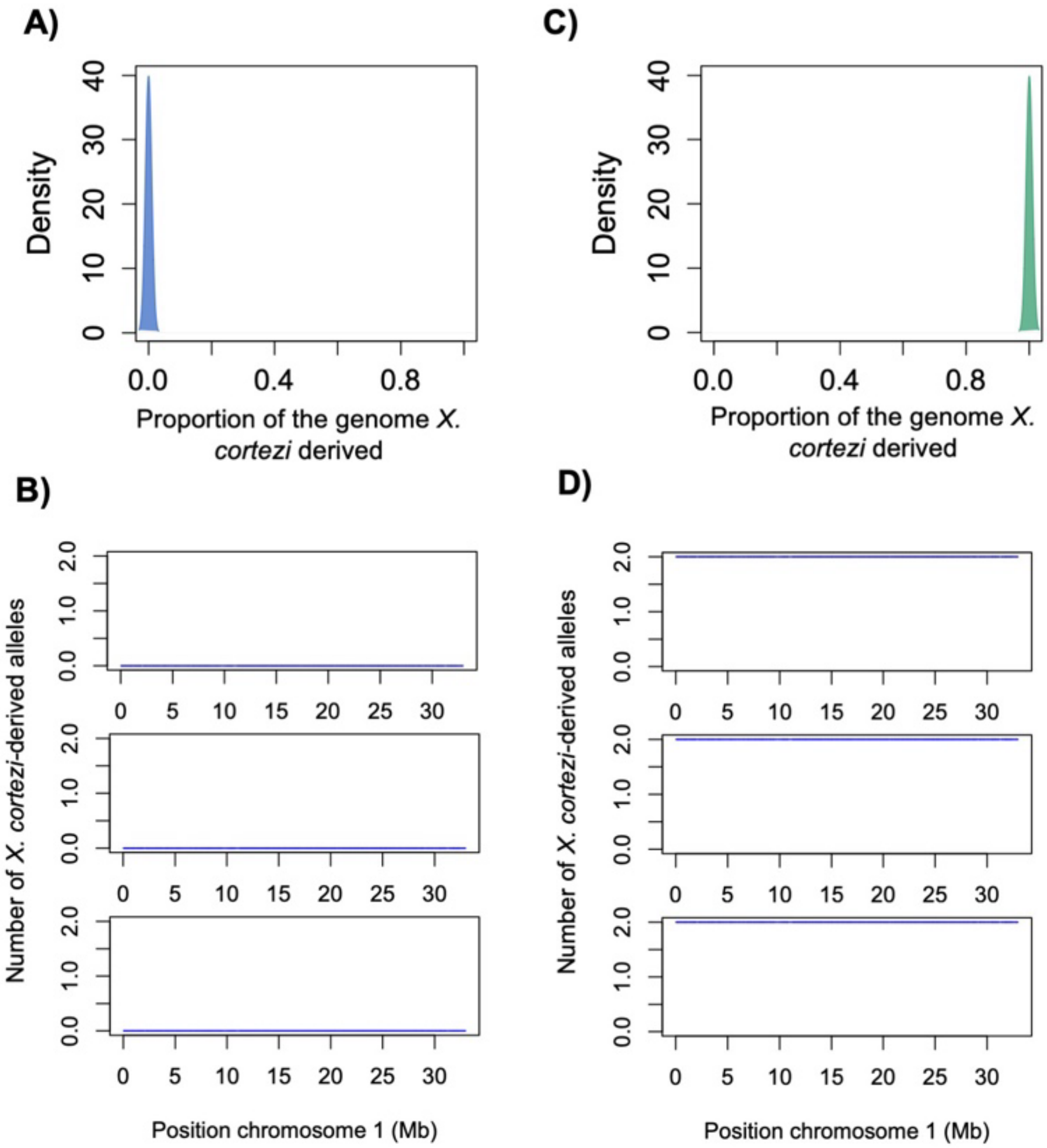

Fig. S13. Performance of local ancestry inference in parental individuals. **A)** Analyses of genome-wide ancestry in allopatric *X. birchmanni* individuals demonstrates that *ancestryinfer* correctly infers that these individuals are homozygous for *X. birchmanni* ancestry across the genome. **B)** This result can also be observed by examining local ancestry in the same individuals (three representative individuals plotted here), where entire chromosomes are inferred to have two *X. birchmanni*-derived and no *X. cortezi*-derived haplotypes. **C**) Similarly, ancestry inference in allopatric *X. cortezi* individuals is accurate, with all individuals inferred to be homozygous *X. cortezi* throughout the genome. **D**) This result can also be observed by examining local ancestry in the same individuals (three representative individuals plotted here), where entire chromosomes are inferred to have two *X. cortezi* derived and no *X. birchmanni*-derived haplotypes. The data underlying this figure can be found in Dryad repository doi:10.5061/dryad.qnk98sfq1.
