# Supplementary material for: Swordtail fish hybrids reveal that genome evolution is surprisingly predictable after initial hybridization
Source: PLoS Biol. 2024 Aug 26;22(8):e3002742. doi: 10.1371/journal.pbio.3002742 (PMC11379403; doi:10.1371/journal.pbio.3002742)
Supplement: S14 Fig — (A) F1 hybrids produced in lab are inferred by ancestryinfer to have precisely 50% of their genome derived from X. birchmanni and 50% from X. cortezi, with essentially no variation in ancestry observed, as expected for this cross. (B) Three representative individuals showing local ancestry across chromosome 1 in F1 hybrids. F1 hybrids are inferred to have 1 X. birchmanni and 1 X. cortezi haplotype across the chromosome, as expected from the cross design. (C) F2 hybrids produced in lab are inferred to have on average 50% of their genome derived from each parental species, but with substantial variation in genome-wide ancestry induced by recombination between the X. birchmanni and X. cortezi haplotypes in their F1 parents, followed by independent assortment. (D) Local ancestry across chromosome 1 for 3 representative F2 individuals. (E) Local ancestry across chromosome 1 for 4 representative BC1 individuals (F1 hybrids crossed to X. cortezi). We produced fewer backcross individuals than other cross types so do not plot genome-wide ancestry for these individuals. The data underlying this figure can be found in Dryad repository doi:10.5061/dryad.qnk98sfq1. (PDF) [file pbio.3002742.s030.pdf]

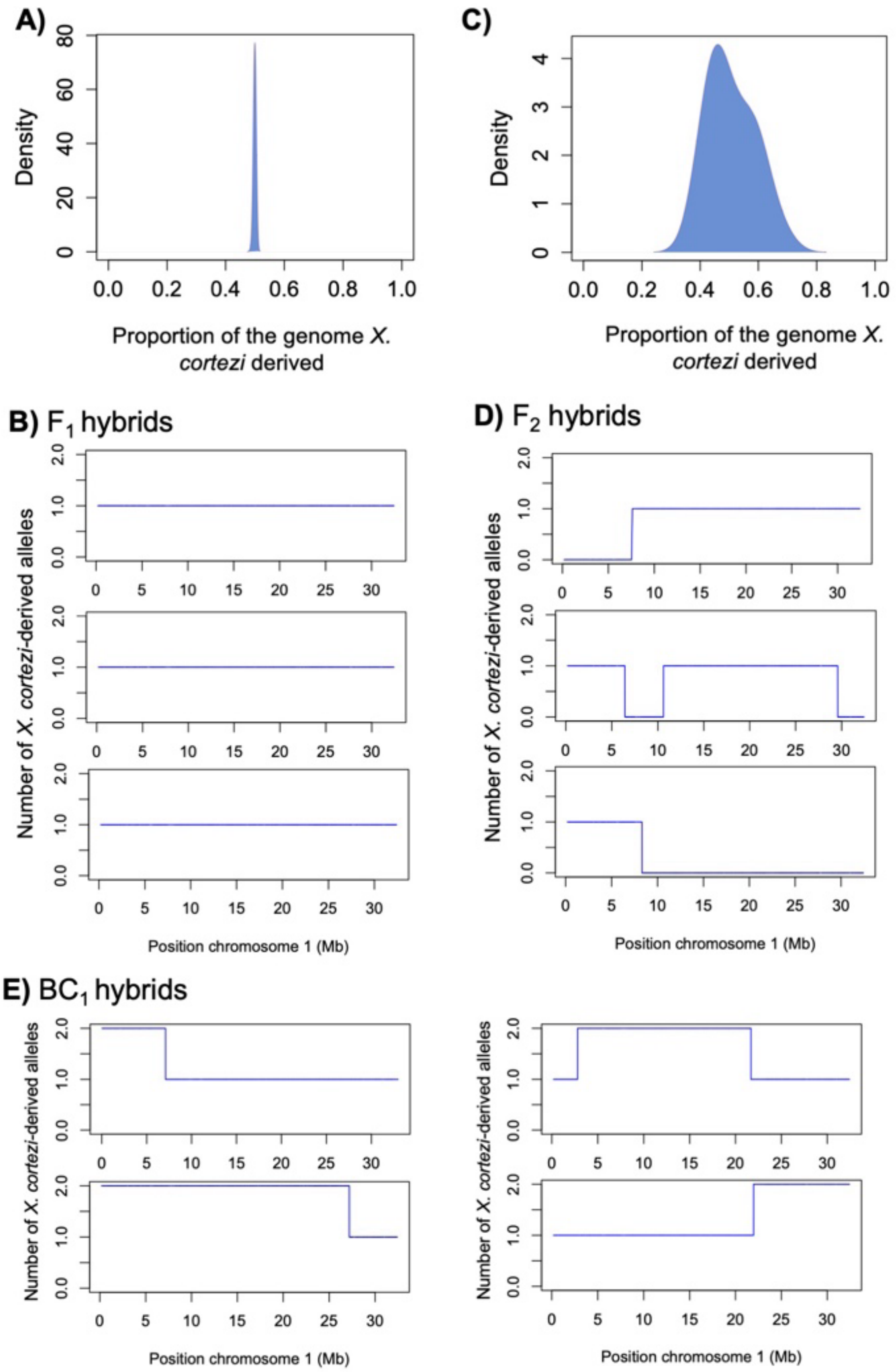

Fig. S14. Analyses of genome-wide ancestry in hybrid offspring of known crosses. **A**) F_1 hybrids produced in lab are inferred by *ancestryinfer* to have precisely 50% of their genome derived from *X. birchmanni* and 50% from *X. cortezi*, with essentially no variation in ancestry observed, as expected for this cross. **B**) Three representative individuals showing local ancestry across chromosome 1 in F_1 hybrids. F_1 hybrids are inferred to have one *X. birchmanni* and one *X. cortezi* haplotype across the chromosome, as expected from the cross design. **C**) F_2 hybrids produced in lab are inferred to have on average 50% of their genome derived from each parental species, but with substantial variation in genome-wide ancestry induced by recombination

between the *X. birchmanni* and *X. cortezi* haplotypes in their F_1 parents, followed by independent assortment. **D**) Local ancestry across chromosome 1 for three representative F_2 individuals. **E**) Local ancestry across chromosome 1 for four representative BC_1 individuals (F_1 hybrids crossed to *X. cortezi*). We produced fewer backcross individuals than other cross types so do not plot genome-wide ancestry for these individuals. The data underlying this figure can be found in Dryad repository doi:10.5061/dryad.qnk98sfq1.
