# Supplementary material for: Swordtail fish hybrids reveal that genome evolution is surprisingly predictable after initial hybridization
Source: PLoS Biol. 2024 Aug 26;22(8):e3002742. doi: 10.1371/journal.pbio.3002742 (PMC11379403; doi:10.1371/journal.pbio.3002742)
Supplement: S15 Fig — As a complementary analysis to ancestry inference in known crosses, we used the program mixnmatch to simulate late generation hybrids with parameters matching the Chapulhuacanito (A) and Santa Cruz (B) populations to estimate our error rate in the case of late generation hybrids between X. cortezi and X. birchmanni. (A) Simulations of Chapulhuacanito hybrids using the MAP estimates of demographic parameters from the posterior distributions generated by ABCreg and with a simulated hybrid population size of 5,000. Accuracy in local ancestry inference for 50 simulated hybrid individuals is shown here. The average error rate in these simulations was 0.3%. (B) Simulations of Santa Cruz using the MAP estimates of demographic parameters from the posterior distributions generated by ABCreg and with a simulated hybrid population size of 5,000. Accuracy in local ancestry inference for 50 simulated hybrid individuals is shown here. The average error rate in these simulations was 0.4%. As expected, the error rate is slightly higher for simulations of Santa Cruz, since this population is likely to be older and smaller ancestry tracts are more difficult to accurately infer. The data underlying this figure can be found in Dryad repository doi:10.5061/dryad.qnk98sfq1. (PDF) [file pbio.3002742.s031.pdf]

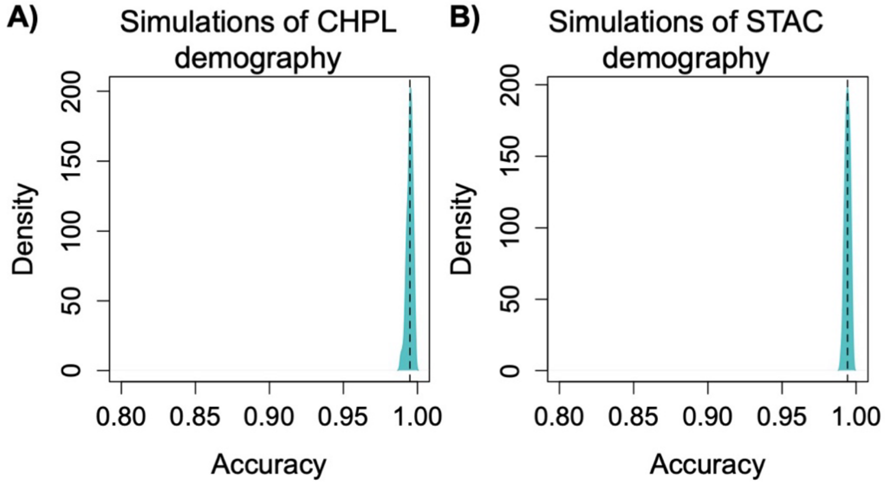

Fig. S15. Estimates of error rate using *mixnmatch* simulations. As a complementary analysis to ancestry inference in known crosses, we used the program *mixnmatch* to simulate late generation hybrids with parameters matching the Chapulhuacanito (**A**) and Santa Cruz (**B**) populations to estimate our error rate in the case of late generation hybrids between *X. cortezi* and *X. birchmanni*. **A)** Simulations of Chapulhuacanito hybrids using the MAP estimates of demographic parameters from the posterior distributions generated by ABCreg and with a simulated hybrid population size of 5,000. Accuracy in local ancestry inference for 50 simulated hybrid individuals is shown here. The average error rate in these simulations was 0.3%. **B**) Simulations of Santa Cruz using the MAP estimates of demographic parameters from the posterior distributions generated by ABCreg and with a simulated hybrid population size of 5,000. Accuracy in local ancestry inference for 50 simulated hybrid individuals is shown here. The average error rate in these simulations was 0.4%. As expected, the error rate is slightly higher for simulations of Santa Cruz, since this population is likely to be older and smaller ancestry tracts are more difficult to accurately infer. The data underlying this figure can be found in Dryad repository doi:10.5061/dryad.qnk98sfq1.
