# Supplementary material for: Swordtail fish hybrids reveal that genome evolution is surprisingly predictable after initial hybridization
Source: PLoS Biol. 2024 Aug 26;22(8):e3002742. doi: 10.1371/journal.pbio.3002742 (PMC11379403; doi:10.1371/journal.pbio.3002742)
Supplement: S16 Fig — (A) Spatial wavelet decomposition of the overall Pearson correlation between inferred minor parent ancestry in Chapulhuacanito (CHPL) and Santa Cruz (STAC) versus the inferred recombination rate, measured at a resolution of 1 kb. (B) The contribution of a given spatial scale to the overall correlation is a weighted correlation of wavelet coefficients for the 2 signals at that scale, weighted by the variances in each signal at that scale, also obtained from the discrete wavelet transform. We show this decomposition for Chapulhuacanito and note that the general pattern is highly similar for Santa Cruz (STAC). The contributions sum to the total Pearson correlation calculated at the finest resolution of measurement (1 kb). For simplicity, we omit “scaling” variances which are leftover variance due irregularity of chromosome lengths. The data underlying this figure can be found in Dryad repository doi:10.5061/dryad.qnk98sfq1. (PDF) [file pbio.3002742.s032.pdf]

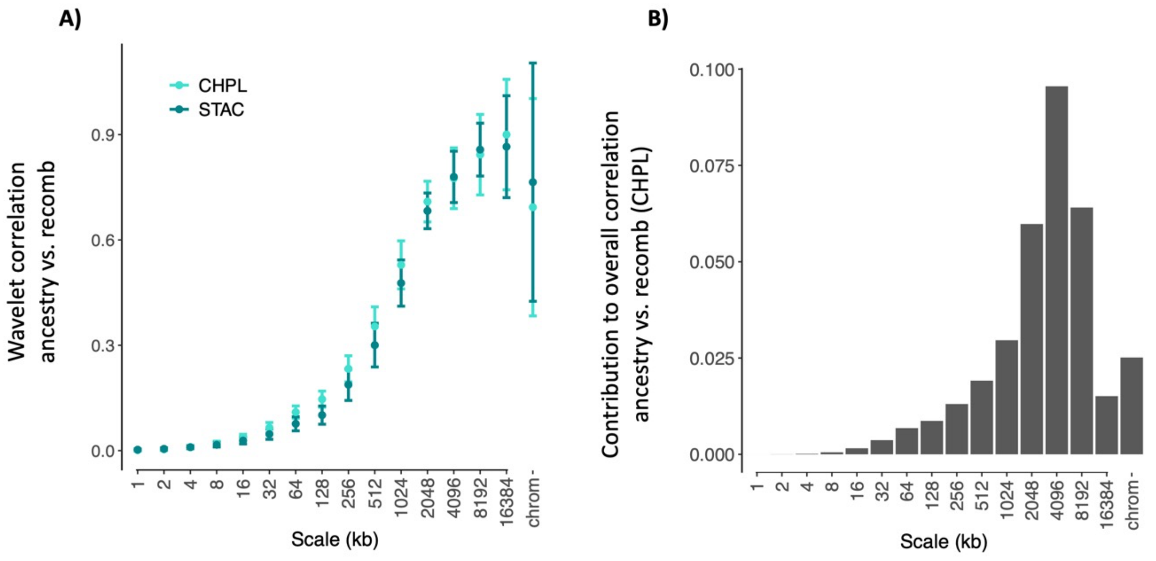

Fig. S16. Additional results of wavelet decomposition analyses focusing on the relationship between minor parent ancestry and recombination rate. **A)** Spatial wavelet decomposition of the overall Pearson correlation between inferred minor parent ancestry in Chapulhuacanito (CHPL) and Santa Cruz (STAC) versus the inferred recombination rate, measured at a resolution of 1 kb. **B)** The contribution of a given spatial scale to the overall correlation is a weighted correlation of wavelet coefficients for the two signals at that scale, weighted by the variances in each signal at that scale, also obtained from the discrete wavelet transform. We show this decomposition for Chapulhuacanito and note that the general pattern is highly similar for Santa Cruz (STAC). The contributions sum to the total Pearson correlation calculated at the finest resolution of measurement (1 kb). For simplicity we omit 'scaling' variances which are leftover variance due irregularity of chromosome lengths. The data underlying this figure can be found in Dryad repository doi:10.5061/dryad.qnk98sfq1.
