# Supplementary material for: Swordtail fish hybrids reveal that genome evolution is surprisingly predictable after initial hybridization
Source: PLoS Biol. 2024 Aug 26;22(8):e3002742. doi: 10.1371/journal.pbio.3002742 (PMC11379403; doi:10.1371/journal.pbio.3002742)
Supplement: S17 Fig — (A) Wavelet correlations between minor parent ancestry proportion in cross population comparisons between hybrids derived from the same hybridizing pair of X. birchmanni × X. cortezi (CHPL vs. STAC), the same hybridizing pair of X. birchmanni × X. malinche populations (ACUA vs. AGZC), and between hybrids from different hybridizing pairs (CHPL vs. ACUA). Points are weighted averages across chromosomes with error bars representing 95% jackknife confidence intervals. For visualization, we omit the confidence interval for the wavelet correlation of ancestry in ACUA vs. AGZC at the largest scale, since it is large and overlaps zero. See discussion in Text J in S1 File regarding fine scale correlations (e.g., 1 kb). (B) Here, the correlations in A are weighted by the variances in ancestry in each population at a given scale to reflect the contribution of that scale to the overall correlation. (C) Partial wavelet correlations of minor parent ancestry between populations after accounting for variation in recombination. At each scale, we compute a wavelet correlation of the residuals for each population from a linear model with recombination as a predictor. Using these values, we find a comparatively larger reduction in comparisons across hybrid population types (i.e., CHPL vs. ACUA). This would be consistent with a larger portion of the ancestry correlation in these comparisons being driven by the shared effects of recombination. The data underlying this figure can be found in Dryad repository doi:10.5061/dryad.qnk98sfq1. (PDF) [file pbio.3002742.s033.pdf]

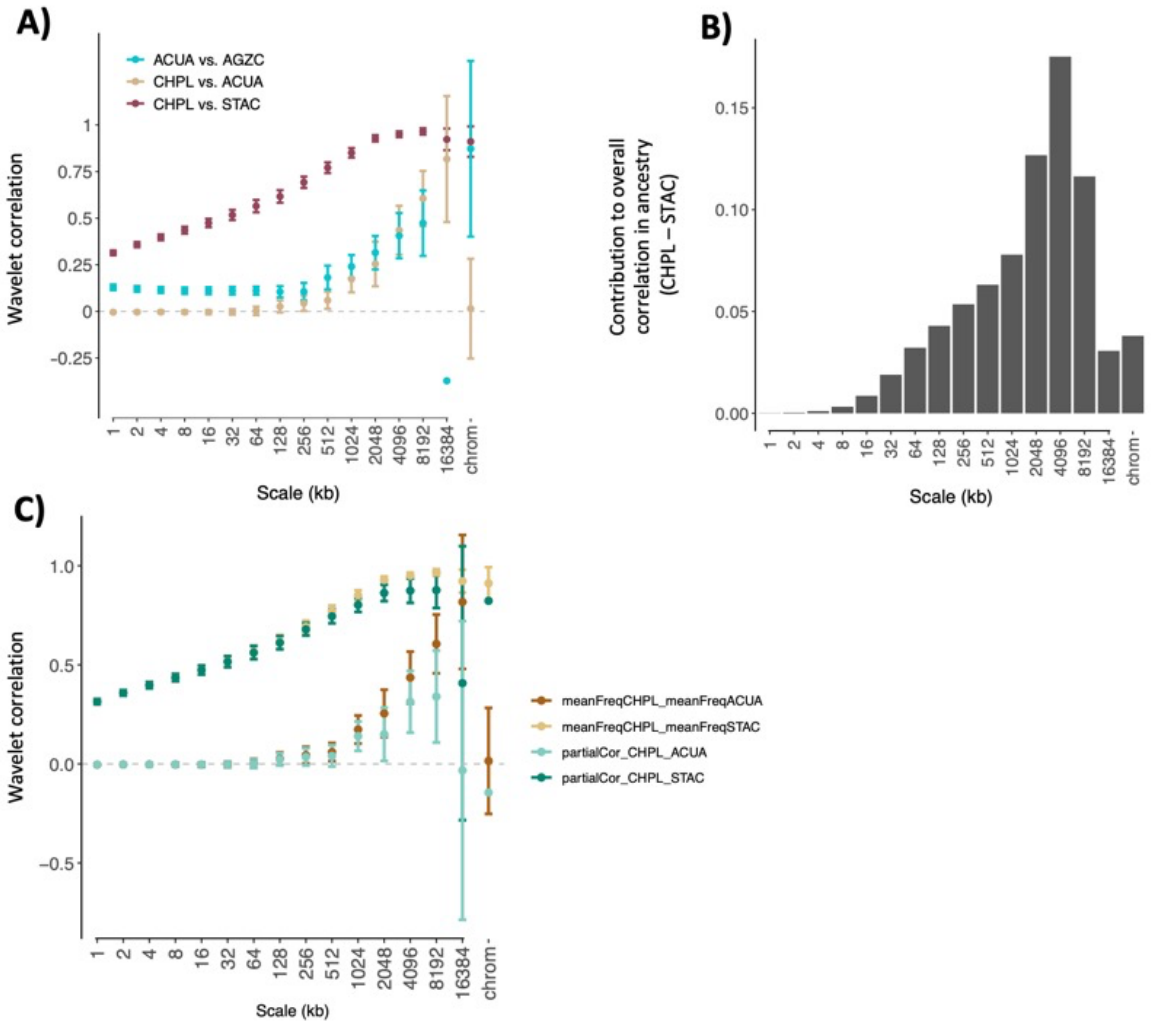

Fig. S17. Additional results of wavelet decomposition analyses focusing on the relationship between minor parent ancestry in different hybrid populations. **A)** Wavelet correlations between minor parent ancestry proportion in cross population comparisons between hybrids derived from the same hybridizing pair of *X. birchmanni* x *X. cortezi* (CHPL vs. STAC), the same hybridizing pair of *X. birchmanni* x *X. malinche* populations (ACUA vs AGZC), and between hybrids from different hybridizing pairs (CHPL vs. ACUA). Points are weighted averages across chromosomes with error bars representing 95% jackknife confidence intervals. For visualization, we omit the confidence interval for the wavelet correlation of ancestry in ACUA vs. AGZC at the largest scale, since it is large and overlaps zero. See discussion in Text J in S1 File regarding fine scale correlations (e.g. 1 kb). **B)** Here, the correlations in **A** are weighted by the variances in ancestry in each population at a given scale to reflect the contribution of that scale to the overall correlation. **C)** Partial wavelet correlations of minor parent ancestry between populations after accounting for variation in recombination. At each scale, we compute a wavelet correlation of the residuals for each population from a linear model with recombination as a predictor. Using these values, we find a comparatively larger reduction in comparisons across hybrid population types (i.e. CHPL vs. ACUA). This would be consistent with a larger portion of the ancestry

correlation in these comparisons being driven by the shared effects of recombination. The data underlying this figure can be found in Dryad repository doi:10.5061/dryad.qnk98sfq1.
