# Supplementary material for: Swordtail fish hybrids reveal that genome evolution is surprisingly predictable after initial hybridization
Source: PLoS Biol. 2024 Aug 26;22(8):e3002742. doi: 10.1371/journal.pbio.3002742 (PMC11379403; doi:10.1371/journal.pbio.3002742)
Supplement: S18 Fig — Minor parent deserts and islands were identified in the X. birchmanni × X. cortezi hybrid populations Chapulhuacanito (CHPL) and Santa Cruz (STAC). We then asked whether these regions overlapped with minor parent deserts or islands found in X. birchmanni × X. malinche hybrid populations (Acuapa–ACUA, Aguazarca–AGZC, and Tlatemaco–TLMC). Black diamonds indicate the observed number of overlaps between minor parent deserts and islands in any X. birchmanni × X. malinche population and the X. birchmanni × X. cortezi hybrid populations (with the number shared between Chapulhuacanito and Santa Cruz shown in blue for comparison). Colored points show the number of shared deserts or islands from replicates jack-knife bootstrapping the genome in 10 cM windows. Gray points show null expectations for each comparison (see Methods). As expected, we observe many more shared deserts and islands among X. birchmanni × X. cortezi hybrid populations than between X. birchmanni × X. cortezi and X. birchmanni × X. malinche hybrid populations. The data underlying this figure can be found in Dryad repository doi:10.5061/dryad.qnk98sfq1. (PDF) [file pbio.3002742.s034.pdf]

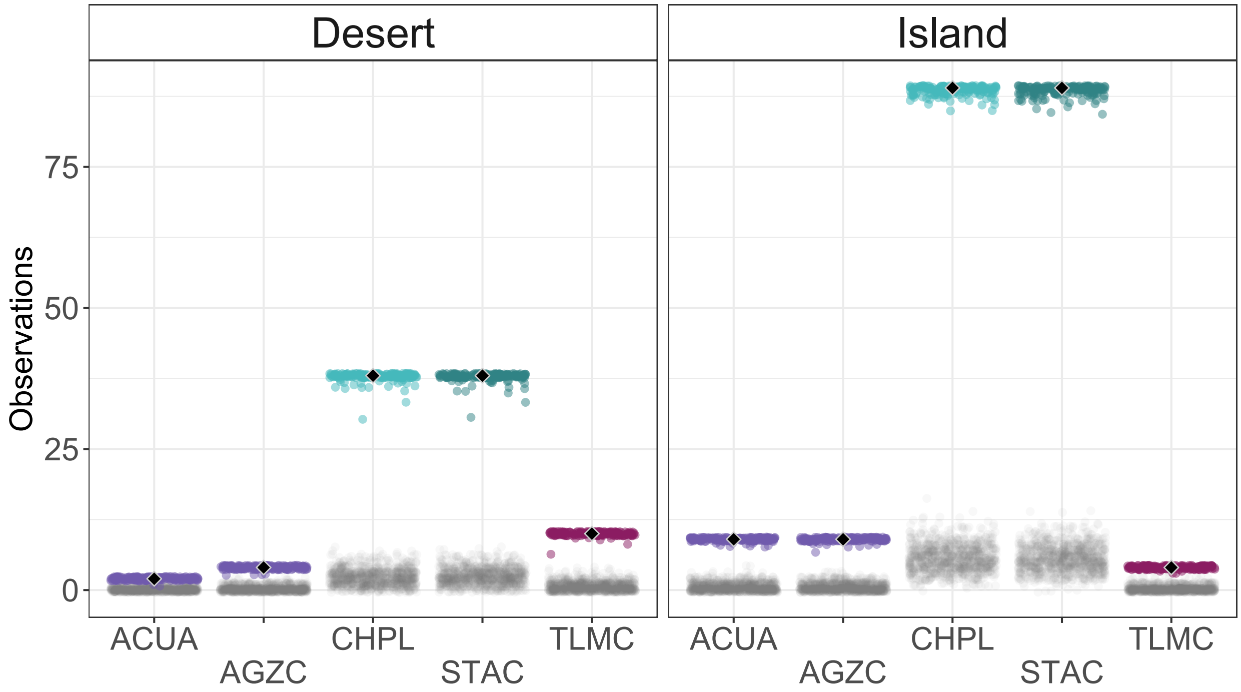

Fig. S18. Observed shared minor parent deserts and islands across hybrid population types. Minor parent deserts and islands were identified in the *X. birchmanni* x *X. cortezi* hybrid populations Chapulhuacanito (CHPL) and Santa Cruz (STAC). We then asked whether these regions overlapped with minor parent deserts or islands found in *X. birchmanni* x *X. malinche* hybrid populations (Acuapa – ACUA, Aguazarca – AGZC, and Tlatemaco – TLMC). Black diamonds indicate the observed number of overlaps between minor parent deserts and islands in any *X. birchmanni* x *X. malinche* population and the *X. birchmanni* x *X. cortezi* hybrid populations (with the number shared between Chapulhuacanito and Santa Cruz shown in blue for comparison). Colored points show the number of shared deserts or islands from replicates jack-knife bootstrapping the genome in 10 cM windows. Gray points show null expectations for each comparison (see Methods). As expected, we observe many more shared deserts and islands among *X. birchmanni* x *X. cortezi* hybrid populations than between *X. birchmanni* x *X. cortezi* and *X. birchmanni* x *X. malinche* hybrid populations. The data underlying this figure can be found in Dryad repository doi:10.5061/dryad.qnk98sfq1.
