# Supplementary material for: Swordtail fish hybrids reveal that genome evolution is surprisingly predictable after initial hybridization
Source: PLoS Biol. 2024 Aug 26;22(8):e3002742. doi: 10.1371/journal.pbio.3002742 (PMC11379403; doi:10.1371/journal.pbio.3002742)
Supplement: S19 Fig — birchmanni (red). Inversions between the 2 species are highlighted by light red lines connecting chromosomes, while co-linear regions are connected by light green lines (based on minimap2 alignments). Gray boxes show schematics of each chromosome. Black circles on the chromosome schematic indicate locations on the chromosomes where telomeric sequences were detected (using seqtk telo). The Circos plot was generated by the R package circlize. Note that several putative translocations are identified, which we treat with caution in the absence of Hi-C data for X. cortezi. The data underlying this figure can be found in Dryad repository doi:10.5061/dryad.qnk98sfq1. (PDF) [file pbio.3002742.s035.pdf]

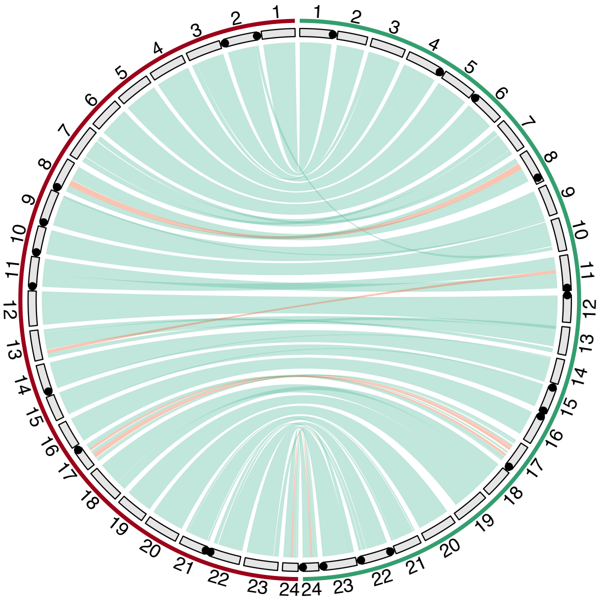

Fig. S19. Circos plot of the new *X. cortezi* genome assembly (green) relative to *X. birchmanni* (red). Inversions between the two species are highlighted by light red lines connecting chromosomes, while co-linear regions are connected by light green lines (based on minimap2 alignments). Gray boxes show schematics of each chromosome. Black circles on the chromosome schematic indicate locations on the chromosomes where telomeric sequences were detected (using seqtk telo). The Circos plot was generated by the R package circulize. Note that several putative translocations are identified, which we treat with caution in the absence of Hi-C data for *X. cortezi*. The data underlying this figure can be found in Dryad repository doi:10.5061/dryad.qnk98sfq1.
