# Supplementary material for: Swordtail fish hybrids reveal that genome evolution is surprisingly predictable after initial hybridization
Source: PLoS Biol. 2024 Aug 26;22(8):e3002742. doi: 10.1371/journal.pbio.3002742 (PMC11379403; doi:10.1371/journal.pbio.3002742)
Supplement: S20 Fig — ndufs5 (red line) is involved in a known mitonuclear incompatibility (see main text) but the region surrounding it was not identified as a shared desert (found as a desert in Chapulhuacanito but not Santa Cruz). However, examination of local ancestry indicates that X. birchmanni ancestry at ndufs5 (red line) is rare in both populations, but was slightly higher than the lowest 5% quantile for Santa Cruz (lowest 5% quantile for X. birchmanni ancestry is 2.2% in Santa Cruz, versus 2.3% X. birchmanni ancestry observed at ndufs5). The data underlying this figure can be found in Dryad repository doi:10.5061/dryad.qnk98sfq1. (PDF) [file pbio.3002742.s036.pdf]

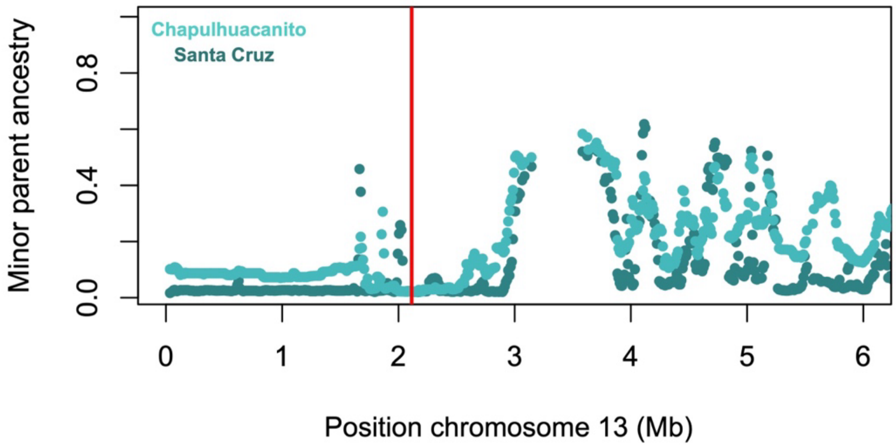

Fig. S20. Plot of average minor parent ancestry across the first six Mb of chromosome 13 in both Santa Cruz and Chapulhuacanito with the location of *ndufs5* highlighted. *ndufs5* (red line) is involved in a known mitonuclear incompatibility (see main text) but the region surrounding it was not identified as a shared desert (found as a desert in Chapulhuacanito but not Santa Cruz). However, examination of local ancestry indicates that *X. birchmanni* ancestry at *ndufs5* (red line) is rare in both populations, but was slightly higher than the lowest 5% quantile for Santa Cruz (Lowest 5% quantile for *X. birchmanni* ancestry is 2.2% in Santa Cruz, versus 2.3% *X. birchmanni* ancestry observed at *ndufs5*). The data underlying this figure can be found in Dryad repository doi:10.5061/dryad.qnk98sfq1.
