# Supplementary material for: Swordtail fish hybrids reveal that genome evolution is surprisingly predictable after initial hybridization
Source: PLoS Biol. 2024 Aug 26;22(8):e3002742. doi: 10.1371/journal.pbio.3002742 (PMC11379403; doi:10.1371/journal.pbio.3002742)
Supplement: S21 Fig — In the “Complexes vs. all proteins” comparison, we calculated average minor parent ancestry in genes involved in protein complexes (see Text M in S1 File) in both Chapulhuacanito and Santa Cruz (colored points and whiskers), compared to null data sets generated from randomly sampling matched sets of protein coding genes (gray points). Observed minor parent ancestry in these proteins is slightly but significantly lower than the null distribution. We next considered the possibility that this signal was driven by genes involved in protein complexes being highly conserved. We identified all genes that had a single reciprocal best blast hit with human proteins (“1:1 orthologs”), excluded genes involved in protein complexes, and repeated the analysis (see Text M in S1 File). We found that these genes were similarly depleted in minor parent ancestry relative to the null, suggesting that the signal we observe with genes in protein complexes is driven by conservation, not directly by their role in protein–protein interactions. The data underlying this figure can be found in Dryad repository doi:10.5061/dryad.qnk98sfq1. (PDF) [file pbio.3002742.s037.pdf]

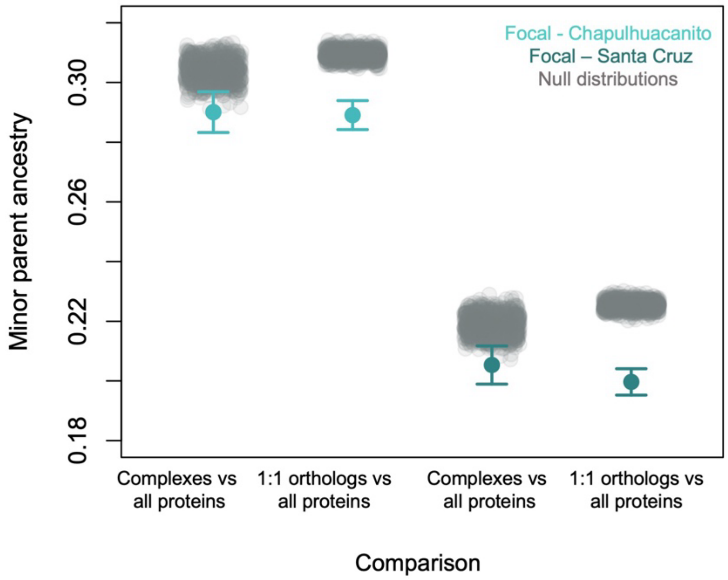


**Fig. S21.** Comparisons of minor parent ancestry across genes of different annotations in the two hybrid populations. In the “Complexes vs all proteins” comparison, we calculated average minor parent ancestry in genes involved in protein complexes (see Text M in S1 File) in both Chapulhuacanito and Santa Cruz (colored points and whiskers), compared to null datasets generated from randomly sampling matched sets of protein coding genes (gray points). Observed minor parent ancestry in these proteins is slightly but significantly lower than the null distribution. We next considered the possibility that this signal was driven by genes involved in protein complexes being highly conserved. We identified all genes that had a single reciprocal best blast hit with human proteins (“1:1 orthologs”), excluded genes involved in protein complexes, and repeated the analysis (see Text M in S1 File). We found that these genes were similarly depleted in minor parent ancestry relative to the null, suggesting that the signal we observe with genes in protein complexes is driven by conservation, not directly by their role in protein-protein interactions. The data underlying this figure can be found in Dryad repository doi:10.5061/dryad.qnk98sfq1.
