# Supplementary material for: Swordtail fish hybrids reveal that genome evolution is surprisingly predictable after initial hybridization
Source: PLoS Biol. 2024 Aug 26;22(8):e3002742. doi: 10.1371/journal.pbio.3002742 (PMC11379403; doi:10.1371/journal.pbio.3002742)
Supplement: S22 Fig — cortezi. Results in A show identified region on chromosome 7 and results in B show identified region on chromosome 14. Each point indicates average minor parent ancestry in a 0.05 cM window in Santa Cruz or Chapulhuacanito. Red line indicates the region identified as under selection in F2 hybrids between X. birchmanni and X. cortezi and black line in B indicates the location of a shared minor parent ancestry desert between Santa Cruz and Chapulhuacanito on chromosome 14. While the region on chromosome 7 does not overlap with a shared minor parent ancestry desert, it does overlap with an ancestry desert in Santa Cruz at approximately 16.5 Mb, and X. birchmanni ancestry across the larger region from 16–22 Mb is relatively low in both Santa Cruz and Chapulhuacanito (in the 15% and 10% quantile of minor parent ancestry genome wide, respectively). These regions are exciting candidates for identifying additional hybrid incompatibilities between X. birchmanni and X. cortezi. The data underlying this figure can be found in Dryad repository doi:10.5061/dryad.qnk98sfq1. (PDF) [file pbio.3002742.s038.pdf]

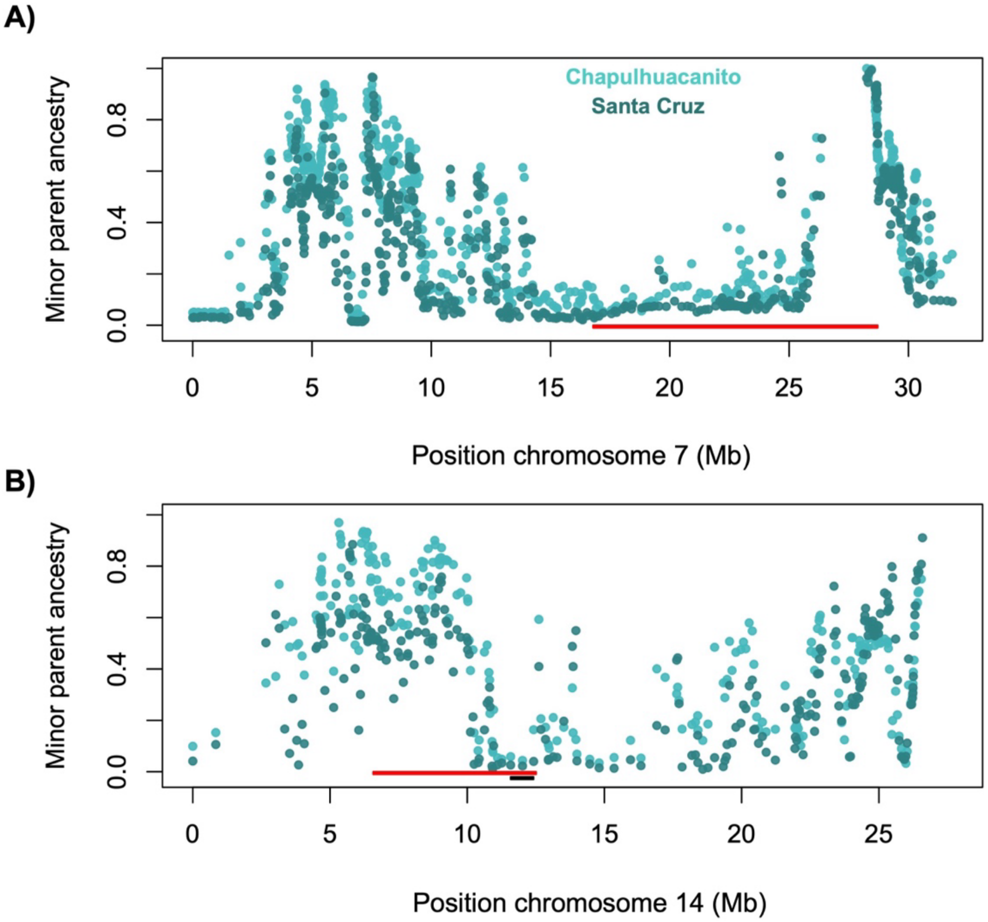

Fig. S22. Average *X. birchmanni* ancestry in Santa Cruz and Chapulhuacanito surrounding two new regions identified as under strong selection in F_2 hybrids between *X. birchmanni* and *X. cortezi.* Results in **A** show identified region on chromosome 7 and results in **B** show identified region on chromosome 14. Each point indicates average minor parent ancestry in a 0.05 cM window in Santa Cruz or Chapulhuacanito. Red line indicates the region identified as under selection in F_2 hybrids between *X. birchmanni* and *X. cortezi* and black line in **B** indicates the location of a shared minor parent ancestry desert between Santa Cruz and Chapulhuacanito on chromosome 14. While the region on chromosome 7 does not overlap with a shared minor parent ancestry desert, it does overlap with an ancestry desert in Santa Cruz at ~16.5 Mb, and *X. birchmanni* ancestry across the larger region from 16-22 Mb is relatively low in both Santa Cruz and Chapulhuacanito (in the 15% and 10% quantile of minor parent ancestry genome-wide, respectively). These regions are exciting candidates for identifying additional hybrid incompatibilities between *X. birchmanni* and *X. cortezi*. The data underlying this figure can be found in Dryad repository doi:10.5061/dryad.qnk98sfq1.
