# Supplementary material for: Swordtail fish hybrids reveal that genome evolution is surprisingly predictable after initial hybridization
Source: PLoS Biol. 2024 Aug 26;22(8):e3002742. doi: 10.1371/journal.pbio.3002742 (PMC11379403; doi:10.1371/journal.pbio.3002742)
Supplement: S23 Fig — Plots show genome-wide admixture proportions at the end of simulations described in Text G in S1 File. A and C show simulations using demographic parameters inferred for the Santa Cruz population from ABCreg, with selection on 40 recessive hybrid incompatibilities (A) or on 40 hybrid incompatibilities with a range of dominance coefficients (C). The dotted gray line shows the observed admixture proportion in Santa Cruz. B and D show simulations using demographic parameters inferred for the Chapulhuacanito hybrid population from ABCreg, with selection on 40 recessive hybrid incompatibilities (B) or on 40 hybrid incompatibilities with a range of dominance coefficients (D). The dotted gray line shows the observed admixture proportion in Chapulhuacanito. For all sets of admix’em simulations, we had to modify the initial admixture proportion from the posterior distributions inferred by ABCreg because simulation selection dramatically shifts the initial admixture proportion (see Text G in S1 File). As a result, we wanted to confirm that after simulations of selection with admix’em, the final admixture proportions roughly matched those observed in the Santa Cruz and Chapulhuacanito hybrid populations. The data underlying this figure can be found in Dryad repository doi:10.5061/dryad.qnk98sfq1. (PDF) [file pbio.3002742.s039.pdf]

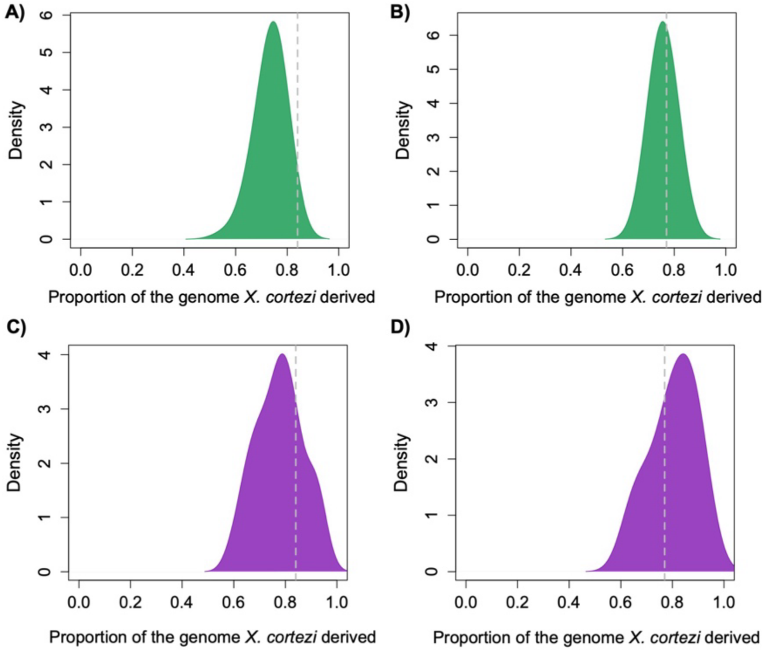

Fig. S23. Results of admix'em simulations with selection. Plots show genome-wide admixture proportions at the end of simulations described in Text G in S1 File. **A** and **C** show simulations using demographic parameters inferred for the Santa Cruz population from ABCreg, with selection on 40 recessive hybrid incompatibilities (**A**) or on 40 hybrid incompatibilities with a range of dominance coefficients (**C**). The dotted gray line shows the observed admixture proportion in Santa Cruz. **B** and **D** show simulations using demographic parameters inferred for the Chapulhuacanito hybrid population from ABCreg, with selection on 40 recessive hybrid incompatibilities (**B**) or on 40 hybrid incompatibilities with a range of dominance coefficients (**D**). The dotted gray line shows the observed admixture proportion in Chapulhuacanito. For all sets of admix'em simulations, we had to modify the initial admixture proportion from the posterior distributions inferred by ABCreg because simulation selection dramatically shifts the initial admixture proportion (see Text G in S1 File). As a result, we wanted to confirm that after simulations of selection with admix'em, the final admixture proportions roughly matched those observed in the Santa Cruz and Chapulhuacanito hybrid populations. The data underlying this figure can be found in Dryad repository doi:10.5061/dryad.qnk98sfq1.
