# Supplementary material for: Swordtail fish hybrids reveal that genome evolution is surprisingly predictable after initial hybridization
Source: PLoS Biol. 2024 Aug 26;22(8):e3002742. doi: 10.1371/journal.pbio.3002742 (PMC11379403; doi:10.1371/journal.pbio.3002742)
Supplement: S24 Fig — For each simulation, we drew demographic parameters from the posterior distributions inferred by ABCreg for Santa Cruz and Chapulhuacanito. We simulated 2 hybrid populations using these demographic parameters as described in Text G in S1 File. In one set of simulations, we implemented selection on 40 pairs of recessive hybrid incompatibilities, with selection coefficients drawn from an exponential distribution with a mean of 0.6. The observed cross-population correlations in ancestry in 250 kb windows for 50 replicates of this simulation scenario are shown in (A). In another set of simulations, we implemented selection on 40 pairs of hybrid incompatibilities as before, except that we drew dominance coefficients from a uniform distribution of 0–0.5 and selection coefficients from an exponential distribution with a mean of 0.4. The observed cross-population correlations in ancestry in 250 kb windows for 50 replicates of this simulation scenario are shown in (B). The inferred cross-population correlation coefficient in 250 kb windows between Santa Cruz and Chapulhuacanito in shown by the dotted gray line. Although the observed value falls in the upper range of the distribution inferred from simulations, these results indicate that selection, combined with the inferred demographic history of these 2 populations, can drive the high correlations in local ancestry we observe. (C) Neutral simulations with no selection implemented are plotted for comparison (see Text G in S1 File). The data underlying this figure can be found in Dryad repository doi:10.5061/dryad.qnk98sfq1. (PDF) [file pbio.3002742.s040.pdf]

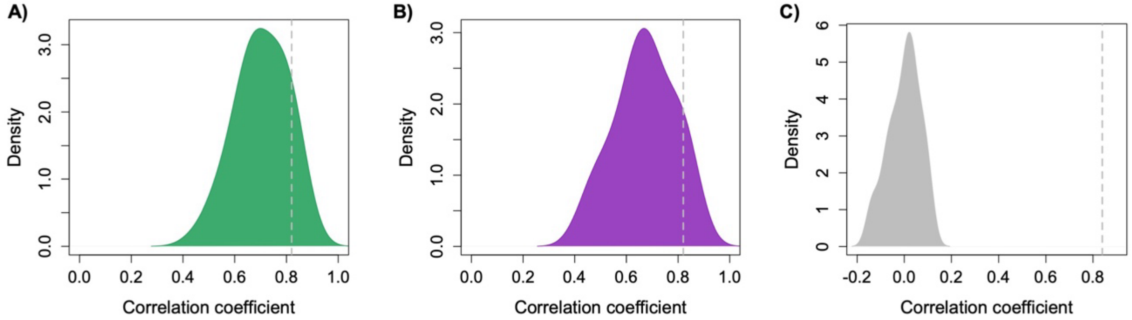

Fig. S24. Results of admix'em simulations modeling shared selection in two independently formed hybrid populations. For each simulation, we drew demographic parameters from the posterior distributions inferred by ABCreg for Santa Cruz and Chapulhuacanito. We simulated two hybrid populations using these demographic parameters as described in Text G in S1 File. In one set of simulations, we implemented selection on 40 pairs of recessive hybrid incompatibilities, with selection coefficients drawn from an exponential distribution with a mean of 0.6. The observed cross-population correlations in ancestry in 250 kb windows for 50 replicates of this simulation scenario are shown in (**A**). In another set of simulations, we implemented selection on 40 pairs of hybrid incompatibilities as before, except that we drew dominance coefficients from a uniform distribution of 0-0.5 and selection coefficients from an exponential distribution with a mean of 0.4. The observed cross-population correlations in ancestry in 250 kb windows for 50 replicates of this simulation scenario are shown in (**B**). The inferred cross-population correlation coefficient in 250 kb windows between Santa Cruz and Chapulhuacanito in shown by the dotted gray line. Although the observed value falls in the upper range of the distribution inferred from simulations, these results indicate that selection, combined with the inferred demographic history of these two populations, can drive the high correlations in local ancestry we observe. **C)** Neutral simulations with no selection implemented are plotted for comparison (see Text G in S1 File). The data underlying this figure can be found in Dryad repository doi:10.5061/dryad.qnk98sfq1.
