# Supplementary material for: Swordtail fish hybrids reveal that genome evolution is surprisingly predictable after initial hybridization
Source: PLoS Biol. 2024 Aug 26;22(8):e3002742. doi: 10.1371/journal.pbio.3002742 (PMC11379403; doi:10.1371/journal.pbio.3002742)
Supplement: S25 Fig — To evaluate the expected performance of ABCreg, we randomly sampled 100 simulations generated for ABC demographic inference. We treated the summary statistics from each simulation as if it were the real data and ran ABCreg. We asked for a given simulation, whether the true value for the focal parameter fell within the 50% quantile of the posterior distribution generated by ABCreg (top) or the 95% quantile (bottom). Plotted are the proportion of simulations where the true value for each parameter fell within the 50% or 95% quantile of the posterior distribution generated by ABCreg. For all parameters, the true value is very likely to fall in the 95% quantile of the posterior distribution generated by ABCreg. The data underlying this figure can be found in Dryad repository doi:10.5061/dryad.qnk98sfq1. (PDF) [file pbio.3002742.s041.pdf]

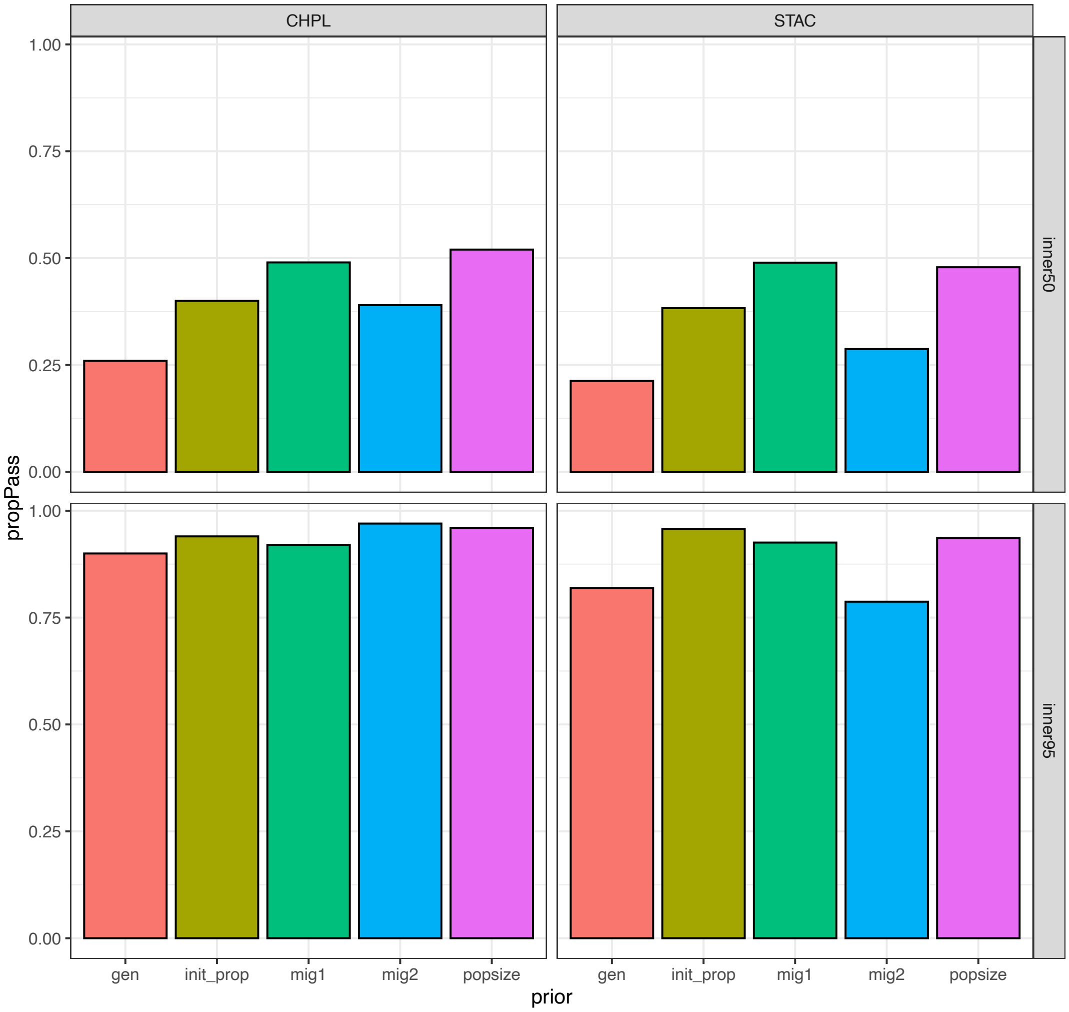

Fig. S25. Analysis of expected performance of ABCreg. To evaluate the expected performance of ABCreg, we randomly sampled 100 simulations generated for ABC demographic inference. We treated the summary statistics from each simulation as if it were the real data and ran ABCreg. We asked for a given simulation, whether the true value for the focal parameter fell within the 50% quantile of the posterior distribution generated by ABCreg (top) or the 95% quantile (bottom). Plotted are the proportion of simulations where the true value for each parameter fell within the 50% or 95% quantile of the posterior distribution generated by ABCreg. For all parameters, the true value is very likely to fall in the 95% quantile of the posterior distribution generated by ABCreg. The data underlying this figure can be found in Dryad repository doi:10.5061/dryad.qnk98sfq1.
