# Supplementary material for: Swordtail fish hybrids reveal that genome evolution is surprisingly predictable after initial hybridization
Source: PLoS Biol. 2024 Aug 26;22(8):e3002742. doi: 10.1371/journal.pbio.3002742 (PMC11379403; doi:10.1371/journal.pbio.3002742)
Supplement: S26 Fig — The number of shared deserts (left) and islands (right) between the Santa Cruz (STAC) and Chapulhuacanito (CHPL) populations compared to expectations by chance, using a different permutation approach that preserves the structure of local ancestry variation along the genome (see Methods). Gray points and boxplots indicate expectations from permutations; black points indicate the observed data. The data underlying this figure can be found in Dryad repository doi:10.5061/dryad.qnk98sfq1. (PDF) [file pbio.3002742.s042.pdf]

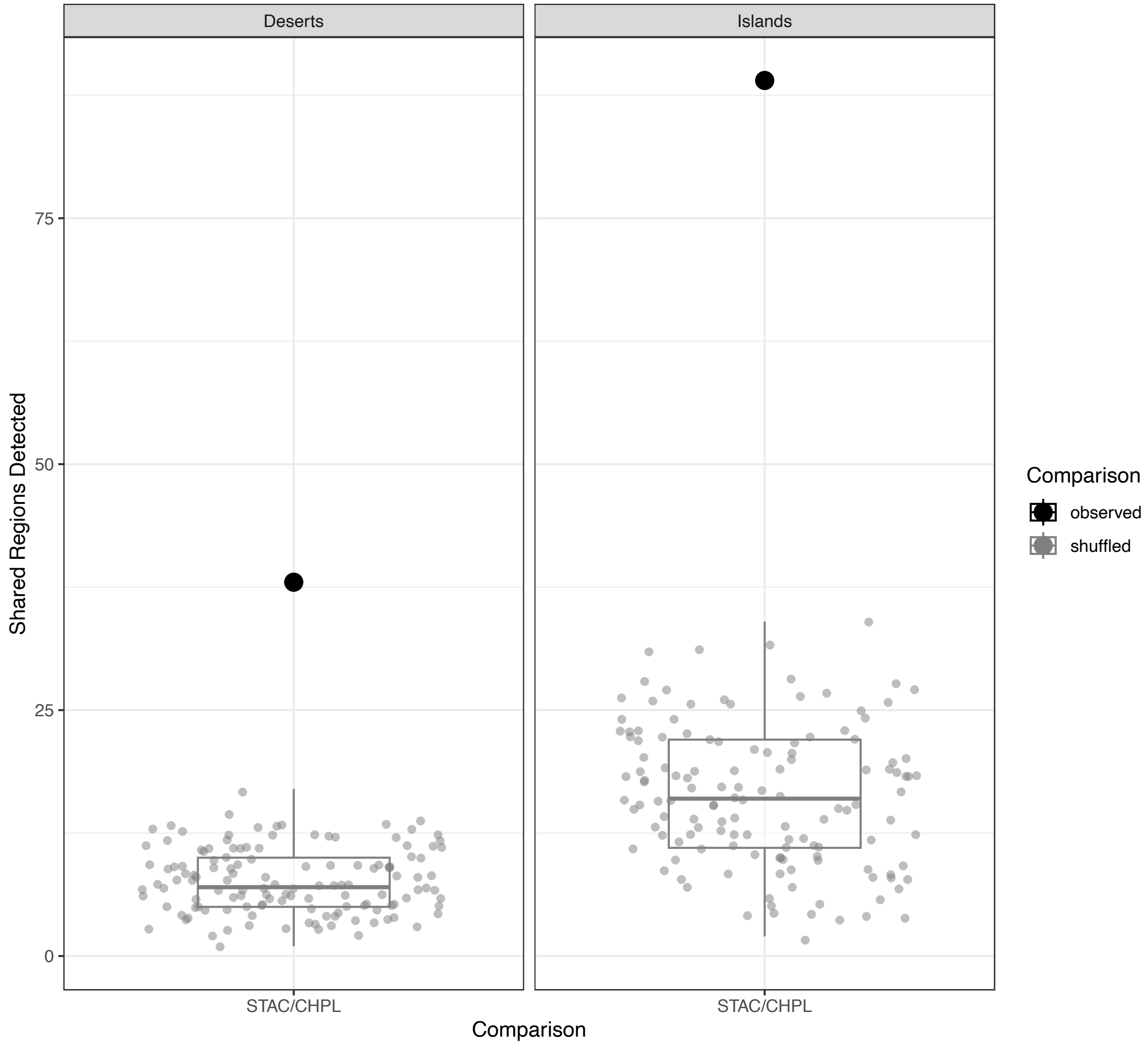

Fig. S26. Alternative analysis of enrichment of shared deserts and islands across hybrid populations. The number of shared deserts (left) and islands (right) between the Santa Cruz (STAC) and Chapulhuacanito (CHPL) populations compared to expectations by chance, using a different permutation approach that preserves the structure of local ancestry variation along the genome (see Methods). Gray points and boxplots indicate expectations from permutations, black points indicate the observed data. The data underlying this figure can be found in Dryad repository doi:10.5061/dryad.qnk98sfq1.
