# Supplementary material for: Swordtail fish hybrids reveal that genome evolution is surprisingly predictable after initial hybridization
Source: PLoS Biol. 2024 Aug 26;22(8):e3002742. doi: 10.1371/journal.pbio.3002742 (PMC11379403; doi:10.1371/journal.pbio.3002742)
Supplement: S27 Fig — Simulations of shared hybrid population history are not consistent with population genetic statistics calculated from our data (Text C in S1 File). We used additional simulations to explore what source population demographic history might be consistent with our data in and ABC framework. Shown here are the results of those simulations; see Text C in S1 File for simulation details. (A) Posterior distribution of generations of genetic drift between the source X. cortezi populations contributing to Santa Cruz and Chapulhuacanito based on 500 accepted simulations. Red line indicates the MAP estimate of 1,315 generations (95% credible intervals: 329–3,099). (B) Posterior distribution of post-split population size 1 based on 500 accepted simulations. Red line indicates the MAP estimate of 2,187 individuals (95% credible intervals: 233–4,851). (C) Posterior distribution of post-split population size 2 based on 500 accepted simulations. Red line indicates the MAP estimate of 979 individuals (95% credible intervals: 193–4,816). The data underlying this figure can be found in Dryad repository doi:10.5061/dryad.qnk98sfq1. (PDF) [file pbio.3002742.s043.pdf]

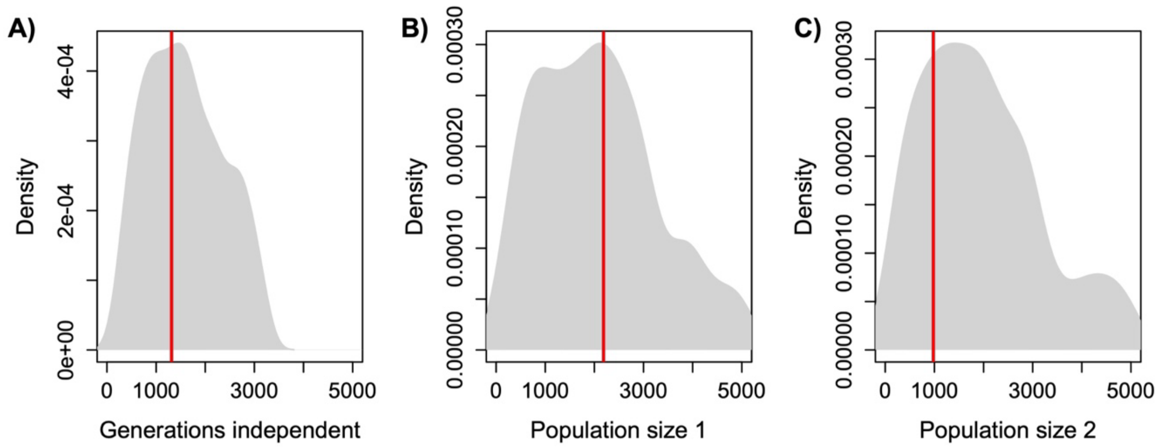

Fig. S27. Inferred separation between source populations of the two hybrid populations at Santa Cruz and Chapulhuacanito based on ABC simulations implemented in SLiM. Simulations of shared hybrid population history are not consistent with population genetic statistics calculated from our data (Text C in S1 File). We used additional simulations to explore what source population demographic history might be consistent with our data in and ABC framework. Shown here are the results of those simulations; see Text C in S1 File for simulation details. **A**) Posterior distribution of generations of genetic drift between the source *X. cortezi* populations contributing to Santa Cruz and Chapulhuacanito based on 500 accepted simulations. Red line indicates the MAP estimate of 1315 generations (95% credible intervals: 329-3099). **B**) Posterior distribution of post-split population size 1 based on 500 accepted simulations. Red line indicates the MAP estimate of 2,187 individuals (95% credible intervals: 233-4851). **C**) Posterior distribution of post-split population size 2 based on 500 accepted simulations. Red line indicates the MAP estimate of 979 individuals (95% credible intervals: 193-4816). The data underlying this figure can be found in Dryad repository doi:10.5061/dryad.qnk98sfq1.
