# Supplementary material for: Swordtail fish hybrids reveal that genome evolution is surprisingly predictable after initial hybridization
Source: PLoS Biol. 2024 Aug 26;22(8):e3002742. doi: 10.1371/journal.pbio.3002742 (PMC11379403; doi:10.1371/journal.pbio.3002742)
Supplement: S28 Fig — In this individual, the local ancestry call switches from heterozygous for ancestry to homozygous X. birchmanni for 11 kb. While errors like these are occasionally detected in our data sets of parental and F1 hybrid individuals, we estimate our overall error rates for these individuals to be approximately 0.1% per ancestry informative site. The data underlying this figure can be found in Dryad repository doi:10.5061/dryad.qnk98sfq1. (PDF) [file pbio.3002742.s044.pdf]

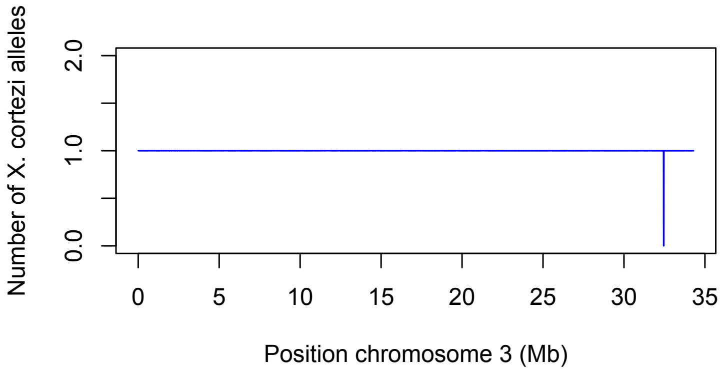

Fig. S28. Example of a likely error in local ancestry inference detected in an F_1 hybrid between *X. birchmanni* and *X. cortezi* on chromosome 3. In this individual, the local ancestry call switches from heterozygous for ancestry to homozygous *X. birchmanni* for 11 kb. While errors like these are occasionally detected in our datasets of parental and F_1 hybrid individuals, we estimate our overall error rates for these individuals to be ~0.1% per ancestry informative site. The data underlying this figure can be found in Dryad repository doi:10.5061/dryad.qnk98sfq1.
