# Supplementary material for: Swordtail fish hybrids reveal that genome evolution is surprisingly predictable after initial hybridization
Source: PLoS Biol. 2024 Aug 26;22(8):e3002742. doi: 10.1371/journal.pbio.3002742 (PMC11379403; doi:10.1371/journal.pbio.3002742)
Supplement: S29 Fig — For each population and selection coefficient (s = 0.01–0.1), we performed 100 pairs of simulations of selection using admix’em. For each pair of simulations, we randomly identified the location of the site under selection and then performed simulations with demographic parameters drawn from the ABCreg posterior distributions for Santa Cruz and Chapulhuacanito, respectively. We then identified minor parent deserts as we had for the real data (see Methods) and determined the proportion of time over 100 simulations that we correctly identified the ancestry desert in each population. The data underlying this figure can be found in Dryad repository doi:10.5061/dryad.qnk98sfq1. (PDF) [file pbio.3002742.s045.pdf]

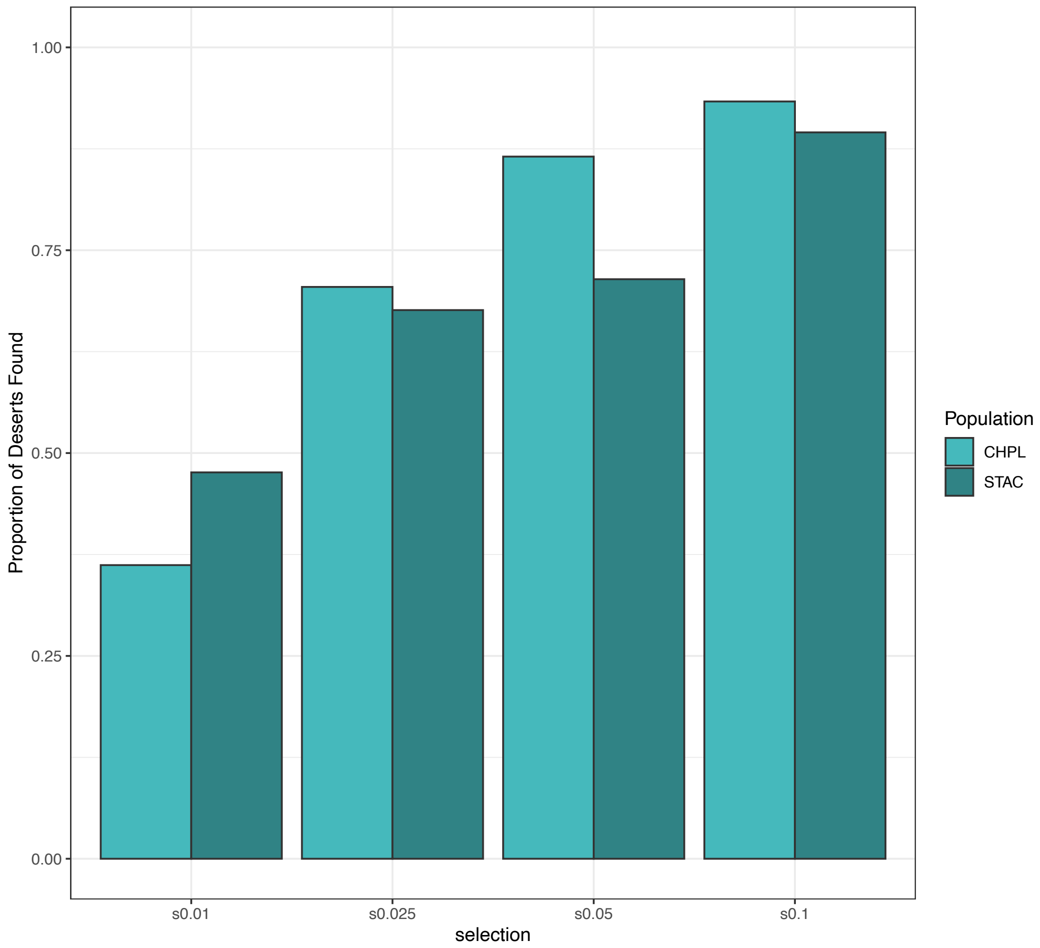

Fig. S29. Simulations of power to detect selected sites in populations matching the demographic history of Santa Cruz (STAC) and Chapulhuacanito (CHPL) populations. For each population and selection coefficient (s=0.01-0.1), we performed 100 pairs of simulations of selection using admix'em. For each pair of simulations, we randomly identified the location of the site under selection and then performed simulations with demographic parameters drawn from the ABCreg posterior distributions for Santa Cruz and Chapulhuacanito, respectively. We then identified minor parent deserts as we had for the real data (see Methods), and determined the proportion of time over 100 simulations that we correctly identified the ancestry desert in each population. The data underlying this figure can be found in Dryad repository doi:10.5061/dryad.qnk98sfq1.
