# Supplementary material for: Swordtail fish hybrids reveal that genome evolution is surprisingly predictable after initial hybridization
Source: PLoS Biol. 2024 Aug 26;22(8):e3002742. doi: 10.1371/journal.pbio.3002742 (PMC11379403; doi:10.1371/journal.pbio.3002742)
Supplement: Attachment — Submitted filename: Langdon_etal_revision_response_to_reviewers_forsubmission.docx [file pbio.3002742.s046.docx]

**Response to reviewers**

**Response to reviewer 1:**

This manuscript utilizes independent Xiphophorus hybrid populations to understand if evolution after hybridization is predictable at the genomic level. The interest of the study is motivated by the growing understanding that hybridization and introgression have contributed to the evolution of many taxa across the tree of life. Yet we do not understand fully the consequences of hybridization and gene flow, because traditionally studies have focused on the consequences of hybridization for speciation and divergence. Now the focus is shifting, and the field is considering hybrids themselves as interesting evolutionary units. This is not the first study to investigate predictability of hybrid genome evolution, and few similar studies have been conducted in other taxa and also in Xiphophorus by the authors themselves. In fact, the current study builds on a wealth of investigations on the Xiphophorus hybrid system. It is this foundation and background knowledge that makes the findings of this study interesting, adding to our understanding of hybrid genome evolution and how hybridization can contribute to evolution in general. [..] This is a very interesting study with wealth of background information on the system, that significantly adds to our understanding of evolution after hybridization. Although some analyses require further elaboration, the study is mainly rigorously executed. The analysis pipelines are diverse and state-of-the-art, partly developed by the authors themselves.

We thank reviewer 1 for their recognition of the importance of this study and for their constructive feedback. We agree that this study adds substantially to our understanding of the genetic and evolutionary outcomes of hybridization. We hope that the added analyses will address reviewer 1’s concerns about the manuscript and further strengthen the conclusions of this work.

1. Did the authors rule out gene flow from parental species into the hybrid populations as one explanation of the observed patterns? For example, say the hybrid populations did not form independently, but from the same event. After separation each of them (or one) received gene flow from one of the parental species. This would create independent recombination breakpoints in the two hybrid populations even when they have the same origin. Then one might end up with repeatable ancestry tracts across hybrid populations but different recombination breakpoints (or ancestry transitions as the authors call them). Did the authors explicitly rule this out? Did they use demographic modelling to test which of the alternative scenarios best fits the data? How have the drainage systems changes in the past >100 generations? Even if they are separated now, is that stable since the formation of hybrids? How far can the fish disperse? Rigorously ruling out alternative demographic scenarios is needed.

Reviewer 1 raises the possibility that the two hybrid populations could have shared some evolutionary history in the early generations after admixture, and later become separated, resulting in partially independent histories. This would be particularly problematic for our analyses because the early generations following hybridization play a disproportionate role in the ultimate patterning of ancestry in the genome. We pursue several lines of inquiry to rigorously address this concern raised by reviewer 1.

First, we have added a supplementary section on the river systems and dispersal capabilities of these fish (Supporting Information 2). Swordtails are small fish (approximately zebrafish sized) and have limited dispersal capabilities in a single generation; previous mark recapture experiments have documented a maximum dispersal distance of <0.5 km. The river distances separating these populations are very large (~130 km). Though dispersal across these distances could be possible over several hundred generations, we would not expect to detect dispersal since the hybrid populations formed in the last ~250 generations. Moreover, these river systems are not likely to have experienced headwater capture over the timescale during which hybridization has occurred.

To bolster these expectations from geography and address the reviewer’s concerns, we have added several new analyses. First, we use IBDseq to analyze patterns of identity-by-descent (IBD) tract sharing within and between geographical locations in individuals for which we have access to high coverage data. We find clear evidence for IBD tracts within hybrid populations, as well as to a lesser extent between hybrid populations and pure *X. birchmanni* and *X. cortezi* populations. In contrast, we find minimal evidence for sharing of IBD tracts between the two hybrid populations. The ~30 tracts we do identify likely originated before the hybridization event given their length distribution. These results are described in Supporting Information 4, Fig. S7-S8, and in the main text (lines 274-280).

Second, we used a series of simulations to investigate the scenario the reviewer described from a genetic perspective. We specifically investigated simulated scenarios where a single hybrid population formed and then split into two populations after 10, 50 or 100 generations of shared evolutionary history. Following the reviewer’s suggestion, we first looked at whether these scenarios could be clearly distinguished in terms of the proportion of shared ancestry transitions. We found that the average number of shared ancestry transitions increased with the number of generations of shared hybrid population evolution, as expected. However, there was high variance in the number of shared junctions between replicate simulations, making it ineffective for addressing this question.

Instead, we decided to evaluate evidence of shared population history based on the “mismatch” statistic we previously presented in the main text. Specifically, we previously identified ancestry tracts where pairs of high coverage individuals were homozygous *X. cortezi* and within these ancestry tracts, calculated the proportion of sites where that pair of individuals were homozygous for a different allele. We reported this ratio for within and between populations comparisons (Fig. 1G in the revised manuscript), and found that mismatch was much higher in comparisons across the two hybrid populations than in any within-population comparison.

This empirical observation presented in the previous version of the manuscript hinted at substantial genetic drift between *X. cortezi* ancestry tracts in the two hybrid populations, suggesting that they formed from divergent source populations. We reasoned that we could use this statistic to investigate whether the degree of mismatch was compatible with shared evolutionary history in the two hybrid populations. To do so, we implemented simulations in SLiM (described in Supporting Information 3), again modeling the formation of a single hybrid population, followed by a split into two hybrid populations at different time points. We found that the degree of mismatch generated by these simulations (which otherwise mimicked inferred population history for the hybrid populations; see Supporting Information 3), was dramatically lower than that observed for cross-population comparisons in the real data (Fig. S5). This suggests that a shared hybrid population history is not compatible with the observed data. Reassuringly, however, these simulations produced mismatch statistics similar to those observed for within-population comparisons in the real data (Fig. S5).

To further investigate this point, we used an approximate Bayesian computation (ABC) approach to jointly estimate the number of generations of genetic drift and source population sizes that could generate the within and between population values for the mismatch statistics observed in the real data (Supporting Information 3). We found that between ~330-3100 generations of drift between the source populations preceding hybridization provided the best fit to our data (Fig. S27).

Together with analyses already presented in the paper, we believe these results clearly indicate that the hybrid populations at Santa Cruz and Chapulhuacanito originated independently and have evolved independently since their formation.

2. The SLiM simulations are the basis of the observation "more repeatable than expected by chance". Did the SLiM simulations incorporate the biological aspects of the model system (pop size, generation time, possible overlapping generations etc.)? If not, is it surprising that the observed parameters do not correspond the simulated ones? Complementing the analyses with simulations that account the demographic history and biological features of the system would make it clear the observed patterns are not expected under neutrality.

We apologize if this was unclear in the initial manuscript. We performed ABC simulations in SLiM to infer the most likely demographic history of the hybrid populations. This included generation time, population size, admixture proportion, and migration rates with both of the parental species. We also implemented system specific parameters such as the observed recombination map, though we did not implement overlapping generations in our simulations. This analysis is described in the methods under section “Demographic inference in the Chapulhuacanito and Santa Cruz populations,” on lines 938-987 of the main text, and in Fig. S1. These analyses resulted in sets of posterior values for different demographic parameters that fit the observed data for each of the two hybrid populations.

To infer the correlations in ancestry across independently formed hybrid populations under neutrality, we then used these posterior distributions to simulate admixture and evolution in hybrid populations using the hybrid population simulator admix’em. For each simulation, we drew a set of demographic parameters for the two populations from their respective posterior distributions, and modeled admixture and evolution under those parameters. After each simulation, we subsampled individuals to match our sample size, summarized ancestry, and compared ancestry correlations across the two simulated populations, as we had for the real data. These analyses are described in Supporting Information 7.

We believe our simulations address exactly the concern that the reviewer raises – that the patterns we observe are not expected from demographic processes under neutrality. We have added additional details to the main text that we hope will make this clearer to the reader (lines 227-228; lines 324-325).

3. There are several passages in the main text where the authors state something is significantly different from random and refer to Figure and methods. It would be more convincing to refer to test statistic or give a short justification why something is different from random, rather than just stating it. e.g. lines 444-446, 453-455.

We have added information to the text addressing these concerns (e.g. lines 456, 466, 491).

4. One of the interesting ideas in this study is that hybrid genome evolution is more repeatable the more distantly related the hybridizing species are. This is what one would expect if the number or strength of selection targets, e.g. incompatibilities, increases with increasing divergence. Results from the study are consistent with this idea. To be able to make a more general statement, one would need more data points on the divergence continuum, since the current study has only two data points (hybrids between closely related Xiphophorus species and hybrids between more distantly related Xiphophorus species). In this sense the results are tentative, but very interesting.

We agree that this is an especially exciting implication of our results and we look forward to continuing this line of investigation in future work. For example, recent work in the lab has identified rare hybridization between *X. variatus* and *X. birchmanni/X. malinche* (~3 million generations diverged; Banerjee & Powell et al. *Evolution* 2023). However, current sample sizes for these hybrids are not sufficient to perform the type of analyses explored here.

5. The authors have also used various techniques (e.g. Li et al. 2022) to infer the location of candidate DMIs. Does the location of these correlate with minor parent ancestry deserts?

One of the challenges of studying DMIs is the availability of appropriate methods to identify them, especially in natural hybrid populations. The methods we have contributed to in past work are appropriate for identifying hybrid incompatibilities in recently formed hybrid populations. For example, the method developed by Li et al. 2022 is effective at identifying unlinked DMIs for ~50 generations following initial admixture, if the hybrid populations formed at relative equal admixture proportions. Since the hybrid populations we study here are substantially older (and skewed in mixture proportion), we do not expect these methods to be effective here.

Our approach for identifying shared ancestry deserts, which we describe in the manuscript, is an alternative method for zeroing in on loci under selection in hybrids that has improved power compared to previous methods. However, a drawback of this approach is that it is not specific in its sensitivity to hybrid incompatibilities and will identify any region where minor parent ancestry is consistently removed.

Nevertheless, to complement our previous results, we have added analysis of two additional regions likely involved in hybrid incompatibilities between *X. birchmanni* x *X. cortezi* hybrids. These two regions were identified in a recent pre-print from our lab (Aguillon et al. *bioRxiv* 2024) based on segregation distortion in F_2_ crosses between *X. birchmanni* and *X. cortezi*. We find that one of these regions overlaps with a shared minor parent ancestry desert, while the other overlaps with a desert in the Santa Cruz population (and has relatively low minor parent ancestry in the Chapulhuacanito population). These new analyses are described on lines 522-532 and Fig. S22.

**Response to reviewer 2**

This manuscript compares two independent populations of hybrids between Xiphophorus birchmanni and X. cortezi, and shows that patterns of local ancestry are strongly correlated between the two populations. The authors then apply a recently developed analytical technique to infer that the correlation results from strong selection in the first few generations after hybridization, and report follow-up analyses to identify the possible targets of selection.

The manuscript is exceptionally thorough and careful in its methodology, and includes well-designed tests to support key premises, such as the independent origin of the two hybrid populations and their respective ages. It's one of the first applications of wavelet analysis of ancestry blocks to study the history of genomic sorting after a pulse of hybridization. The analysis of minor parent ancestry "islands" and "deserts" appears to be highly effective at identifying genomic regions that have experienced selection in hybrids, based on simulations, temporal comparisons, and detection of a previously identified mitonuclear incompatibility between the parent species.

We thank the reviewer for their comments and their recognition of the rigor and novelty of our approach, which we believe is further strengthened in the revised manuscript.

The overall conclusion of the paper extends previous research by the same group, particularly a study (Langdon et al 2022 PLOS Genetics) comparing two birchmanni x cortezi hybrid populations in the same river drainage to multiple birchmanni x malinche hybrid populations (birchmanii is more closely related to malinche than to cortezi). The 2022 study, and previous work by the same group focused on the birchmanni x malinche hybrid populations, found some degree of repeatability in patterns of local ancestry across multiple hybrid populations, and correlations between minor parent ancestry and genomic features such as recombination rate and gene density. The current study finds a higher level of repeatability, which is attributed to strong intrinsic incompatibilities between the genomes of X. birchmanni and X. cortezi. This study is likely to be of great interest to speciation genomics researchers, perhaps more for the comprehensive and innovative set of methods the authors employ than for the major conclusions. However, it may not have as much relevance to scientists outside this subfield, given the similarity to previous research on repeatability of ancestry patterns in replicated swordtail hybrid populations.

We agree that there are methodological similarities between the approaches used in this manuscript and Langdon et al. 2022. However, we believe the results presented here are strikingly different and notable for precisely the reason the reviewer identifies: that increased genetic divergence between the source species is linked to remarkably repeatable genome evolution after hybridization. The only study we are aware of to document such strikingly high levels of repeatable genome evolution in natural populations is Nouhaud et al. *PLoS Biology* 2022. We are able to make further advances here applying novel wavelet-based approaches and time series data that demonstrate how rapidly after initial admixture this repeatability in ancestry along the genome emerged.

While we understand the reviewer’s critique that this work may be of particular interest to researchers studying hybridization and speciation, we believe that it tackles fundamental questions in genetics and evolutionary biology, and thus will be of broad interest to the readership of PLoS Biology. Importantly, the ubiquity of genetic exchange across species makes questions about the predictability of genomic outcomes after hybridization of very general interest (e.g. Racimo et al. *MBE* 2017; Taylor & Larson *Nature Ecology & Evolution* 2019; Benton et al. *Nature Reviews Genetics* 2021). Moreover, our work tackles one of the most classic questions in evolutionary biology – whether evolution is predictable – in a novel context. We have revised the text in the introduction and discussion and hope it now better reflects these important points relating to the novelty of our work.

Minor specific comments:

L83-84: Punctuation error? The phrase "([15]; and the sites under selection are shared)" is hard to parse whether the condition applies to ref 15, the text preceding the citation, or both.

This has been corrected.

L146: Change ref [32] to [33]

This has been corrected.

L181: Change "manuscript refer" to "manuscript we refer"

This change has been made.
